# The complete genome assembly of *Astragalus membranaceus*: enabling more accurate genetic research

**DOI:** 10.1093/gigascience/giaf117

**Published:** 2025-10-01

**Authors:** Huibin Qin, Aohui Li, Shuyu Zhong, Huazhi Wang, Hongling Tian

**Affiliations:** Center for Agricultural Genetic Resources Research, Shanxi Agricultural University/Institute of Crop Germplasm Resources, Shanxi Academy of Agricultural Sciences, Key Laboratory of Crop Gene Resources and Germplasm Enhancement on Loess Plateau, Ministry of Agriculture, Shanxi Key Laboratory of Genetic Resources and Genetic Improvement of Minor Crops, Taiyuan 030031, China; College of Agronomy, Shanxi Agricultural University, Taigu 030801, China; College of Agronomy, Shanxi Agricultural University, Taigu 030801, China; College of Agronomy, Shanxi Agricultural University, Taigu 030801, China; Shanxi Agricultural University, Shanxi Academy of Agricultural Science, The Industrial Crop Institute, Taiyuan 030031, China

**Keywords:** *Astragalus membranaceus*, telomere-to-telomere genome, genome annotation, previously unassembled regions, structural variations

## Abstract

**Background:**

*Astragalus membranaceus* (Fisch.) Bunge is a globally significant medicinal plant renowned for its potent immunomodulatory and antioxidant properties. However, the existing reference genome for this species remains incomplete, characterized by fragmented assemblies and the absence of centromeric and telomeric regions, thereby limiting comprehensive exploration of the genetic mechanisms underlying its key traits.

**Findings:**

We hereby present the first complete genome assembly for *A. membranaceus* (Fisch.) Bge “AM-T2T,” achieved through the integration of PacBio HiFi, ultra-long Oxford Nanopore Technologies, and Hi-C sequencing. The assembly achieved a total size of 1.39 Gb with an N50 of 180.45 Mb. The genome exhibits remarkable completeness (99.63% BUSCO completeness; long terminal repeat assembly index of 22.67) and high accuracy (quality value of 57.51; Genome Continuity Inspector score of 36.23). It features annotations of 64.22% repetitive sequences, 16 telomeres, 8 centromeres, 32,600 high-confident genes, 248 cytochrome P450 monooxygenases (CYP450s), and 163 uridine diphosphate glycosyltransferases. Notably, 158.58 Mb of previously unassembled regions were resolved, harboring 4 CYP450s. Additionally, 2,267 unique genes and 20,652 conserved genes were identified within the AM-T2T genome. Comparative analysis with *Astragalus mongholicus* assembly revealed 1,413 structural variations.

**Conclusions:**

This complete genome assembly of *A. membranaceus* represents a significant advancement in the genomic characterization of *A. membranaceus*, providing a robust resource that will bolster genetic research, breeding programs, and medicinal applications.

## Data Description

### Context

*Astragalus membranaceus* (Fisch.) Bunge (NCBI:txid649199), a key species in the Fabaceae family, has served as a fundamental component of traditional pharmacopeias across Asian civilizations for millennia. Its dried roots, known as “Huangqi” or *Astragali Radix*, have been utilized for over thousands of years in traditional Chinese medicine [1]. This medicinal plant accumulates diverse bioactive compounds, including flavonoids, triterpenoids, polysaccharides, and amino acids. Flavonoids not only participate in plant defense against biotic and abiotic stresses but also exhibit significant antioxidant and anti-inflammatory activities beneficial to human health [2–4]. Triterpenoids such as astragalosides have demonstrated pharmacological effects like enhancing immunity, protecting the cardiovascular system, and exhibiting antitumor properties [5, 6]. Recent studies have further expanded our understanding of its therapeutic potential. Yu et al. [7] reported that *A. membranaceus* attenuates peritoneal fibrosis in both *in vivo* and *in vitro* models by suppressing DNA methyltransferase activity. This effect correlated with improved histopathology, reduced mesothelial–mesenchymal transition biomarkers, and downregulated DNMT1/DNMT3a expression. Mechanistically, it alleviates fibrosis via Dnmt3a-mediated epigenetic regulation of ID2 promoter methylation through the PI3K/Akt pathway, establishing DNA methylation as a critical target for suppressing fibrogenesis. Additionally, Shenshuaikang enema, a formulation derived from *A. membranaceus*, has shown significant efficacy and safety in treating chronic kidney disease by restoring intestinal barrier function via regulation of the microbiota–gut–kidney axis [8]. Astragenol, another bioactive component, alleviates neuroinflammation and ameliorates Parkinson’s disease symptoms through modulation of amino acid metabolism and inhibition of ferroptosis [9]. Such multifaceted biological activities and therapeutic properties have established *A. membranaceus* as a valuable resource for drug discovery and functional food development, driving the need for deeper insights into its genetic and metabolic mechanisms.

Recent advances in genomics have deepened our understanding of *A. membranaceus* biology. Zhang et al. [10] decoded the first complete mitochondrial genome, revealing a multichromosome structure and providing insights into the evolutionary mechanisms of this medicinal plant. Li et al. [11] reconstructed the full-length transcriptome using PacBio Iso-seq technology, identifying numerous transcript variants involved in the biosynthesis of bioactive compounds such as astragalosides and calycosin. Furthermore, Wang et al. [12] identified 76 WRKY transcription factors (TFs) in *A. membranaceus*, with ​AmWRKY8​ conferring drought resistance through hormonal signaling and nuclear-localized transcriptional activation. Concurrently, ​AmMYB35​ was shown to upregulate flavonol biosynthesis under drought stress [13]. At the metabolic engineering level, direct injection of Rhizobium rhizogenes carrying an AmUGT15 overexpression cassette into *A. membranaceus* stem explants induced hairy roots with significantly enhanced astragaloside biosynthetic capacity [14].

*De novo* genome assembly is a fundamental and powerful tool in genomics research. Recently, 2 *A. membranaceus* genome assemblies have been developed using different sequence platforms: one 1.43-Gb assembly (AM-CLR) ​was generated​ using Pacific Biosciences (PacBio) continuous long reads (CLRs) and chromatin conformation capture (Hi-C) technology, achieving a contig N50 of 1.67 Mb [15]. Another​ 1.47-Gb chromosome-level genome assembly (AM-ONT) employed MGI-SEQ short-read, Oxford Nanopore (ONT) long-read, and Hi-C technologies (AM-ONT) [16]. ​Additionally,​​ the genome of *Astragalus mongholicus* (AMM), another authorized plant source of Astragali Radix, has been decoded [17]. However, these assemblies remain incomplete in repetitive sequence regions, centromeres, and telomeres, limiting comprehensive understanding of genetic mechanisms governing bioactive compound biosynthesis and key agronomic traits.

Telomere-to-telomere (T2T) assembly represents a critical advancement for deciphering complex genomes.​​ Recent technological breakthroughs have made T2T genome assembly feasible, enabling comprehensive genome identification. PacBio high-fidelity (HiFi) sequencing generates highly accurate long-read datasets with mean read lengths of 10–25 kb and >99.9% base accuracy. The primary determinant of assembly complexity is not the size of the genome but the repetitive sequence. ONT has addressed this challenge through ultra-long-read methods producing reads averaging ∼50 kb (with lengths exceeding 100 kb), overcoming limitations posed by repetitive regions refractory to HiFi assembly. Leveraging these methodologies, T2T genomes have been achieved for staple crops such as rice [18], maize [19], and sorghum [20]. Methodological frameworks for T2T assembly have also been systematically reviewed, providing robust guidance for genomic studies of medicinal plants [21, 22].

Therefore, to address the genomic gaps in *A. membranaceus*, **​**we integrated PacBio HiFi sequencing, ONT ultra-long sequencing, and Hi-C technology to construct its T2T genome assembly. This enabled, for the first time, comprehensive characterization of telomeric and centromeric regions. Leveraging this T2T genome, we identified PUR regions and annotated genes within these regions. Furthermore, genome-wide investigations were performed for CYP450s and uridine diphosphate glycosyltransferases (UGTs), while unique and conserved gene sets were systematically analyzed. This T2T genome assembly marks a significant advancement in *A. membranaceus* genomics, providing a solid foundation for diverse downstream comparative genomic analyses and pan-genome studies.

## Methods

### Sample collection

**Figure 1:**
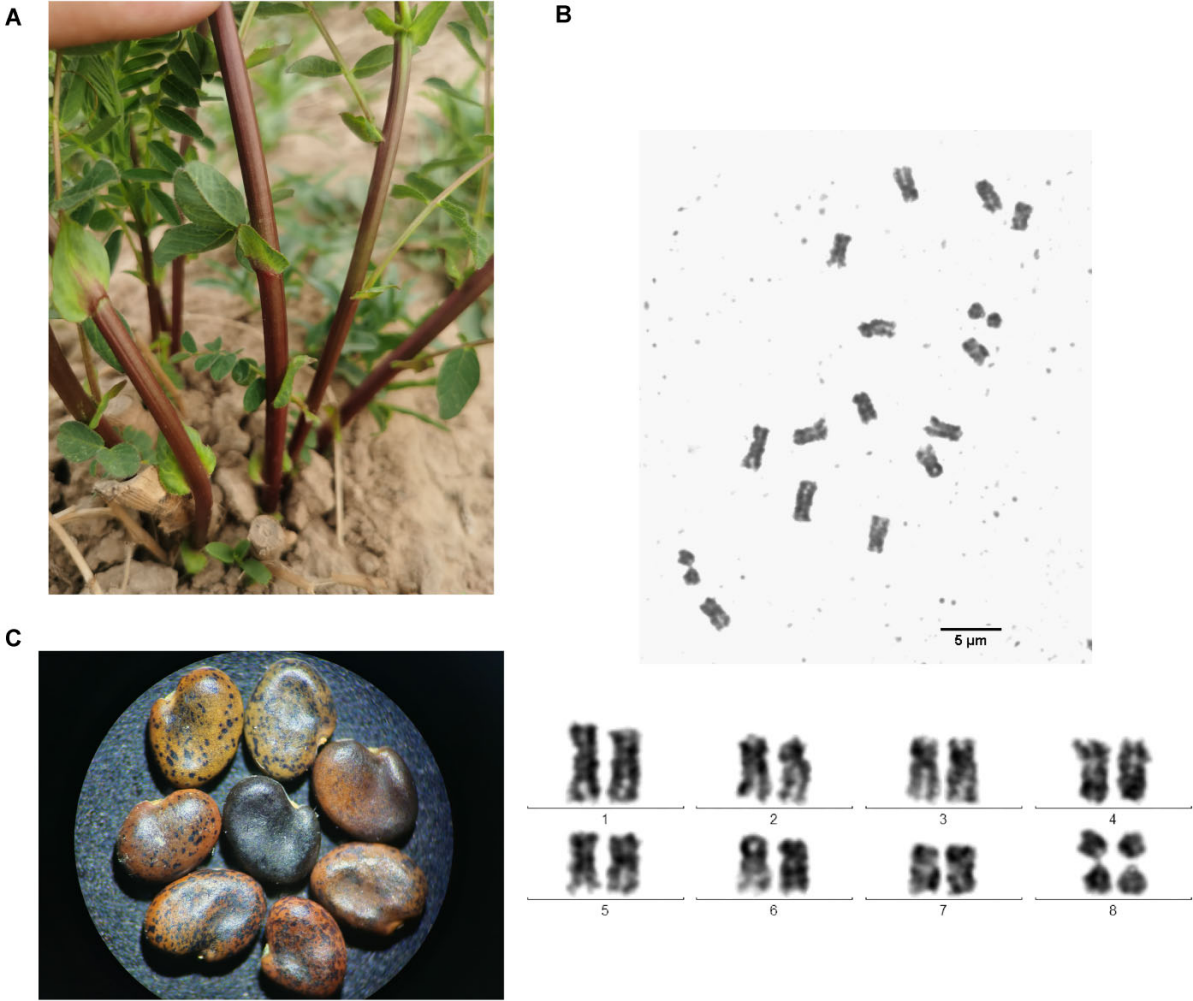
The morphology and karyotype of a *Astragalus membranaceus* (Fisch.) Bunge. (A) The stems of *A. membranaceus*. (B) The karyotype of *A. membranaceus* via karyotype analysis. (C) The seeds of *A. membranaceus*.

The study materials (SXHQ0000254) used in this study was collected from the Zhengyao Garden of Gansu University of Traditional Chinese Medicine (Fig. 1A, C). The voucher specimen is currently deposited in the medicinal plant experimental field of the Fenyang Economic Research Institute, located in Lvliang City, Shanxi Province. High-quality genomic DNA was extracted from healthy young leaves. All samples were frozen in liquid nitrogen and stored at −80°C for preservation and subsequent analysis.

### Karyotype analysis

Root tips were excised from germinated seeds and pretreated in a solution containing 0.001 mol/L 8-hydroxyquinoline and 0.02% colchicine (1:1, v/v) at 4°C for 4 hours to synchronize mitotic cells at metaphase. After thorough rinsing, samples were fixed in freshly prepared Carnoy’s fixative (methanol/acetic acid, 3:1, v/v) for 4–24 hours and hydrolyzed in 1 mol/L HCl at 60°C for 10 minutes. To improve cell wall digestion, enzymatic treatment with a cellulase–pectinase mixture (6%:4%, 2:1, v/v) was performed at 37°C for 5–6 hours, followed by a low-osmotic treatment in distilled water at 37°C for 30 minutes. Chromosomes were stained with carbol fuchsin, and slides were prepared using a standardized squash protocol. Chromosome images were captured using a Nikon 80i microscope equipped with a cold CCD camera, and karyotype parameters were analyzed using Zeiss Karyotype software.

### Sequencing and filtering

For HiFi sequencing, SMRTbell target size libraries were constructed according to PacBio’s standard protocol (Pacific Biosciences) using the approximately 16-kb preparation solutions with the SMRTbell Express Template Prep Kit 2.0. The sequencing was conducted in HiFi mode on the PacBio Revio platform (RRID:SCR_017990) at BGI Genomics. The PacBio HiFi reads, initially generated in BAM format, were converted to FASTQ format using the bam2fastq tool (version 1.0.0) [17]. For ONT sequencing, ONT ultra-long insert libraries were obtained using the Oxford Nanopore SQK-LSK109 kit and sequenced on the PromethION (RRID:SCR_017987) platform. The ONT data underwent processing using NanoFilt version 2.8.020 (RRID:SCR_016966) [23] with a quality threshold of 7. Previous studies of AM-CLR provided RNA sequencing (RNA-seq) and Hi-C reads for supplementary analysis.

### Genome assembly and Hi-C scaffolding

We used a comprehensive strategy for T2T assembly (Supplementary Fig. S1). Four assembly tools were employed to generate contigs using diverse datasets: Hifiasm version 0.19.9 (RRID:SCR_021069) [24], Wtdgb2 version 2.5 (RRID:SCR_017225), Flye version 2.9.4 (RRID:SCR_017016) [25], and NextDenovo version 2.5.2 (RRID:SCR_025033) [26]. Specifically, ultra-long ONT reads were processed using NextDenovo version 2.5.2 (RRID:SCR_025033) [26], while HiFi reads were assembled into contigs using Flye version 2.9.4 (RRID:SCR_017016) [25] and Wtdbg2 version 2.5 (RRID:SCR_017225) [27]. The backbone contigs were generated with Hifiasm version 0.19.9 (RRID:SCR_021069) [24] using the command “hifiasm -o AM.asm -t 50 –ul-cut 1000 –n-hap 2 –telo-m TTTAGGG –h1$HiC_fq1 –h2$HiC_fq2 –ul $ont $hifi.” Hi-C reads (accession number: SRR27790545) were utilized to anchor contigs into chromosomes via Haphic version 1.0.6 [28]. An additional error correction step was carried out with Juicebox version 2.13.07 (RRID:SCR_021172) [29] according to the interaction signal. Contigs obtained by NextDenovo version 2.5.2 (RRID:SCR_025033) [26], Flye version 2.9.4 [25], and Wtdbg2 version 2.5 (RRID:SCR_017225) [27] were used for filling gaps with quartet_gapfiller.py script from quarTeT version 1.1.1 [30]. As recommended, the specific parameters used were “-f 5000 -l 1000 -i 40 -m 1000000 -t 20.” Remaining gaps in assembled chromosomes were closed using the LR_Gapcloser (RRID:SCR_017021) [31] program with HiFi reads, following the T2T assembly protocol for sorghum [20]. To enhance genome quality, Winnowmap version 2.03 (RRID:SCR_025349) [32] was used to align HiFi reads to the chromosomes, followed by filtering to exclude secondary alignments and excessive clipping with the “falconc bam-filter-clipped” tool. Finally, Racon version 1.5.0 (RRID:SCR_017642) [33] was performed for further polishing with the filtered alignments.

The completeness of the genome assembly was assessed utilizing BUSCO version 5.4.3 (RRID:SCR_015008) [34] with the embryophyta_odb10 database, which included 1,614 orthologs. The quality value (QV) was evaluated by Merqury program version 1.3 (RRID:SCR_022964) [35] with 17-mer. Long reads from ONT and HiFi were aligned to the assembly with Minimap2 version 2.24-r1122 (RRID: SCR_018550) [36]. After identifying long terminal repeat (LTR) structures and using complete LTR elements to calculate the LTR assembly index (LAI) value, we performed calculations to determine the genome assembly integrity, which was quantified using the LAI score [37]. In addition, the Genome Continuity Inspector (GCI) was assessed using GCI version 1.0 [38]. Last, a new reference-free tool, Clipping information for Revealing Assembly Quality (CRAQ), was employed to scan the regions of low quality in the genome assembly [39].

### Identification of PUR regions

The AM-CLR and AM-ONT genomes were aligned to the AM-T2T assembly using Winnowmap version 2.03 (RRID:SCR_025349) [32] and Minimap2 version 2.24-r1122 (RRID:SCR_018550) [36], respectively. The parameters employed for Winnowmap and Minimap2 were as follows: -ax asm20 -t 20 -H –MD $ref $qurey > out.sam. The alignment SAM file was converted to PAF format using paftools.js with the following command: paftools.js sam2paf -p out.sam > out.paf. Sequence regions in the AM-T2T assembly that remained uncovered (with mapping quality [MAPQ] > 0) were extracted using the following commands: cat out.paf |awk “{if ($12 > 0) print $6“\t”$8“\t”$9}” | bedtools sort -i—|bedtools merge -i—|bedtools complement -i—-g chr.len > out.pur.region.

### Genome annotations

The content of repetitive sequences in the AM-T2T was predicted using homology searching and the *ab initio* prediction method. For homology-based prediction, RepeatMasker version 4.0.7 [40] and RepeatProteinMask version 4.0.7 were used to search against RepBase. For *ab initio* prediction, LTR_FINDER version 1.07 (RRID:SCR_015247) [41] and RepeatModeler version 1.0.8 were carried out. Tandem Repeats Finder version 4.10 (RRID:SCR_022193) [42] was used to identify the tandem repeat elements.

The gene prediction process employed a comprehensive strategy that integrated transcriptome-based and homology-based methods. Initially, RNA-seq clean reads were assembled using Trinity version 2.8.5 (RRID:SCR_013048) [43], with the parameters “–max_memory 200 G –CPU 40 –min_contig_length 200 –genome_guided_bam merged_sorted.bam –full_cleanup –min_kmer_cov 3 –min_glue 3 –bfly_opts ‘-V 5 –edge-thr=0.1 –stderr’ –genome_guided_max_intron 10000,” yielded 221,161 transcripts with an N50 size of 1,636 bp. The assembled transcripts were then aligned to the assembly using Program to Assemble Spliced Alignment (PASA) version 2.4.1 (RRID:SCR_014656) [44], generating gene structures from valid transcript alignments (PASA-set). Additionally, RNA-seq clean reads were mapped to the assembly via Hisat2 version 2.0.1 (RRID:SCR_015530) [45]. Subsequently, Stringtie version 1.2.2 (RRID:SCR_016323) [46] and TransDecoder version 5.7.1 (RRID:SCR_017647) were employed to assemble the transcripts and identify candidate coding regions, resulting in the creation of gene models (Stringtie-set). Homologous genomes from 7 assemblies, including AM-CLR, AMM [17], *Arabidopsis thaliana* Col-PEK [47], *Glycine max* (ZH13-T2T) [48], *Trifolium pratense* (ensembl release-59), *Phaseolus vulgaris* (ensembl release-59), and *Medicago truncatula* (ensembl release-59), were downloaded and used as queries to search against the assembly using GeMoMa version 1.9 (RRID:SCR_017646) [49]. These homology predictions were referred to as “Homology-set.” The gene models from these 3 sources were subsequently merged using EvidenceModeler version 2.1.0 (RRID:SCR_014659) [50], with different weight parameters assigned to evidence from different sources (10 for Homology-set, 5 for Stringtie-set, and 5 for PASA-set). Finally, the generated gene models underwent further refinement with PASA version 2.4.1 [44] to obtain untranslated regions and alternative splicing variation information. The integrated gene set was translated into amino acid sequences and annotated using the method described the telomere-to-telomere genome assembly of sorghum [20]. Diamond version 0.9.30 (RRID:SCR_009457) [51] with an E-value cutoff of 1e-05 was used to compare the protein against 4 public databases, including NCBI nonredundant protein sequence database, SwissProt [52], Kyoto Encyclopedia of Genes and Genomes (KEGG) [53], and Translation of European Molecular Biology Laboratory. Gene Ontology (GO) terms of these genes were identified using InterProScan version 5.59–91.0 (RRID:SCR_005829) [54].

Gene expression analysis was conducted using the fragments per kilobase of transcript per million mapped reads (FPKM) method, following the approach applied in the blister beetle transcriptome study [55]. Briefly, RNA-seq clean reads were mapped to the reference genome using Bowtie2 version 2.5.4 (RRID:SCR_016368) with the following parameters: -q –phred33 –sensitive –dpad 0 –gbar 99999999 –mp 1,1 –np 1 –score-min L,0,-0.1 -I 1 -X 1000 –no-mixed –no-discordant -p 8 -k 200 [56]. Gene expression levels were subsequently quantified using RSEM version 1.3.3 (RRID:SCR_000262) with default parameters [57]. Differential gene expression (DGE) analysis was performed using the DESeq2 version 1.46.0 (RRID:SCR_015687) [58]. TF prediction was implemented following the method described in the eggplant genome study [59]. Transfer RNAs (tRNAs) and ribosomal RNAs (rRNAs) were predicted using tRNAscan-SE version 1.3.1 (RRID:SCR_008637) [60] and BLASTN (RRID:SCR_001598; *E*-value ≤ 1e−05) against the rRNA sequences of both *A. thaliana* and *Oryza sativa*, respectively. Both microRNAs (miRNAs) and small nuclear RNAs (snRNAs) were identified by searching against the Rfam database (RRID:SCR_010835, release 12.0) using Infernal version 1.1.1 (RRID:SCR_010835).

### Telomere and centromere identification

Following a method similar to that described in the study of the complete broomcorn millet assembly [61], we used quarTeT (RRID:SCR_025258) version 1.1.5 with TeloExplorer to identify telomeres and CentroMiner to identify centromeres [30]. Given the complex structure of centromeres, we further employed Centromics (RRID:SCR_025253) to identify centromeres by detecting high-copy tandem repeats from HiFi sequencing data.

### Genome-wide identification of CYP450s and UGTs

To identify full-length CYP450 candidates in the AM-T2T genome and AMM genome, we employed the PF00067 hidden Markov model (HMM) profile from InterPro. The HMMER version 3.4 was utilized for candidate extraction with the following parameters: -E 1e-5, followed by length filtration​ (exclusion of sequences with <400 or >600 amino acids) [62]. *A. thaliana* CYP450 reference sequences were acquired from the CYP450 database [63]. Multiple sequence alignments were generated with MAFFT version 7.526, and nonconserved regions were filtered using trimAl version 1.4.1 [64, 65]. Phylogenetic trees of CYP450 genes were constructed using IQ-TREE2 version 2.3.6 with the following parameters: -nt AUTO -bb 1000 -pre iqtree [66].

For UGT identification, ​we applied the same pipeline​ used for CYP450s. The UGT HMM profile (PF00201) was retrieved from InterPro, and *A. thaliana* UGT reference sequences were obtained from the Plant UGTs database [67]. Only genes with amino acid lengths between 350 and 600 were retained.

### Gene families analysis

Protein sets of 9 species (*A. mongholicus, M. truncatula, A. thaliana, Cicer arietinum, Cajanus cajan, G. max, M. truncatula, Lupinus angustifolius, Vigna angularis*, and AM-T2T) were employed in the orthology identification with *A. thaliana* as the out-group. The OrthoMCL version 2.0.9 (RRID:SCR_007839) was applied to determine and cluster gene families among these 9 plant species. A total of 1,153 single-copy orthologs among these species were multiply aligned with Muscle version 3.8.1551 (RRID:SCR_011812). The alignments were concatenated and converted into a super-gene alignment in Phylip format and then used for constructing a phylogenomic tree using IQtree2 version 2.3.6 with parameters of “-B 1000 -m MFP.” GO enrichment was conducted using ClusterProfiler version 4.2.2 (RRID:SCR_016884) to explore the functional characteristics of the unique gene families in the AM-T2T genome.

### Identification of structural variants between AM-T2T and AMM

Genome alignment between the AM-T2T and AMM genomes was performed using the NUCmer program of MUMmer4 version 4.0.0rc1 (RRID: SCR_018171) [68] with the following parameter settings: –mum -g 1000 -c 90 -l 40. Subsequently, the delta-filter program was employed to identify alignment blocks using the parameters -r -q -l 1000. Structural variants (SVs) larger than 50 bp were detected using Assemblytics based on the filtered results. A gene was designated as an “SV gene” if at least 30% of its regulatory regions (defined as ±2-kb flanking sequences in this study) or coding sequence (CDS) overlapped with an SV. KEGG pathway analysis was performed using KOBAS version 2.0.12 [69].

## Results

### Complete genome assembly and completeness evaluation

The somatic chromosome complement of *A. membranaceus* displayed a diploid constitution of 2n = 2x = 16, consistent with previous cytogenetic investigations on this species and its variants [70, 71] (Fig. 1B).

We newly sequenced the genome of *A. membranaceus*, generating 92.74 Gb (∼66.72× coverage) of PacBio HiFi reads and 38.53 Gb (∼ 27.72× coverage) of ONT reads (Supplementary Table S1). Four distinct software packages were employed for genome assembly based on different data types. Among these, Hifiasm produced the most contiguous genome assembly using the mixed dataset, with a contig N50 of ∼120.48 Mb and the fewest contig sequences (Supplementary Table S2). Thus, this assembly served as the backbone for scaffolding contigs, while contigs from the other assemblies were used for downstream gap-filling analysis. As a result, a total of 1.41 Gb of Hifiasm assembly sequences were anchored to 8 pseudochromosomes, with 8 gaps distributed across 6 of the pseudochromosomes (Supplementary Table S3). One gap was closed using preassembled contigs, and the remaining 7 gaps were filled using PacBio HiFi reads. After further polishing, a gap-free reference genome designated as AM-T2T was generated, containing a total length of 1.39 Gb (Table 1).

**Table 1:** Assembly statistics of *A. membranaceus* genome assembly

Genomic feature	AM-T2T	AM-ONT (16)	AM-CLR (15)
Number of contigs (gaps)	8 (0)	1,060 (1,032)	1,773 (1,432)
Chromosome number	8	9	9
Assembly length (Mb)	1,386.22	1,439.71	1,431.18
Contig N50 (Mb)	180.45	2.82	1.67
Scaffold N50 (Mb)	180.45	184.69	184.46
HiFi reads mapping rate (%)	100.00	—	—
HiFi reads coverage (%)	99.97	—	—
Number of telomeres	16	1	6
Protein-coding genes number	32,600	38,398	29,914
Repeat content (%)	64.22	68.20	67.98
Genome BUSCOs (%)	99.63	93.37	97.27
GCI score	36.23	—	-
LTR assembly index	22.67	—	16.22
Quality value	57.51	—	48.58

**Figure 2:**
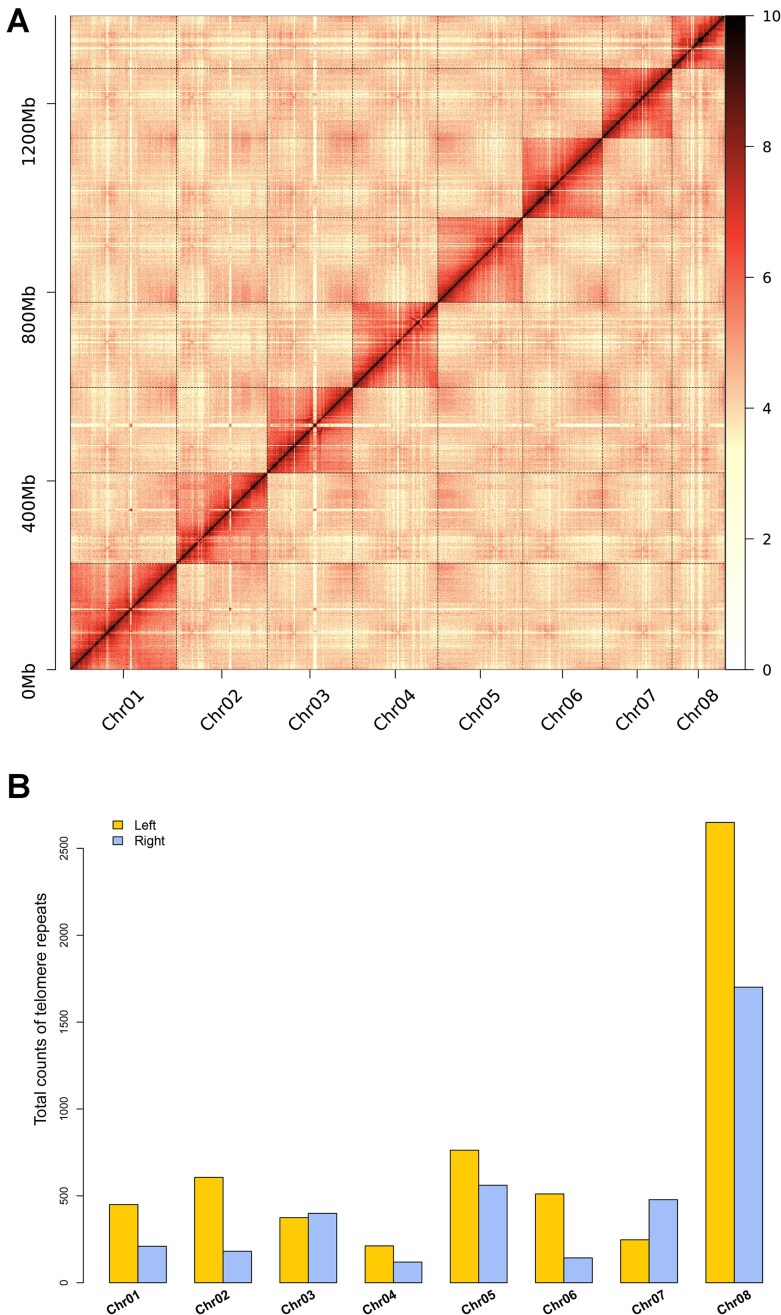
Genomic characteristics of AM-T2T assembly. (A) Intensity signal heatmap of the Hi-C chromosome interaction. The color block illuminates the intensity of interaction from yellow (low) to red (high). (B) The statistics of telomere repeats.

To assess the accuracy and completeness of the AM-T2T assembly, various methods were employed. First, PacBio HiFi reads were mapped onto the genome, yielding a 100% mapping rate and 99.97% genome coverage (Table 1). In particular, the CRAQ analysis revealed that only 0.02% of the genome was classified as low-confidence (Supplementary Table S4). Within these low-confidence regions, repetitive sequences accounted for 72.60%, significantly higher than the 64.22% of repetitive sequences in the entire genome, which may be the primary reason for their low-confidence classification. Second, the BUSCO analysis demonstrated that the completeness of the AM-T2T genome reached 99.63%, exceeding those of the AM-ONT and AM-CLR assemblies (Table 1). Notably, the RNA-seq mapping analysis revealed that AM-T2T was more suitable for analyzing RNA-seq data, with higher mapping rates (average mapping rate: 88.58%) compared to AM-CLR (average mapping rate: 86.15%) and AM-ONT (average mapping rate: 81.17%) (Supplementary Table S5). Third, the LAI value of the AM-T2T assembly was 22.67, meeting the gold standard for genome assemblies [37]. Fourth, the calculated QV of the AM-T2T assembly was 57.51, indicating a base call accuracy higher than 99.999% [35]. Fifth, the Hi-C heatmap demonstrated a high degree of consistency across all pseudochromosomes, confirming the precision in sequencing, ordering, and orientation of contigs (Fig. 2A). The GCI score of the AM-T2T genome was 36.23 (Table 1), significantly surpassing the GCI score of the chicken complete genome (29.37) [38]. Using the 7-base telomeric repeat as a sequence query, we identified all the 16 telomeres for the genome (Fig. 2B). Finally, comparative analysis between the AM-T2T genome and 2 publicly available *A. membranaceus* genomes (AM-ONT and AM-CLR) identified 158.58 Mb of previously unassembled regions (PURs; Supplementary Table S6). Taken together, these comprehensive validation results collectively demonstrate the exceptional quality and reliability of the AM-T2T genome assembly.

### Genome annotation

Repetitive sequences in the AM-T2T genome were annotated using a combination of *de novo* and homology-based approaches, identifying 890.27 Mb of repeats, comprising 64.22% of the genome (Supplementary Table S7). This repetitive fraction was slightly smaller than those observed in the AM-CLR and AM-ONT (Table 1). The predominant repeat classes in AM-T2T were LTR retrotransposons and DNA transposons, which constituted 55.60% and 5.54% of the genome, respectively (Supplementary Table S8). Notably, 123.25 Mb (77.72%) of the PURs consisted of repetitive sequences. A total of 16,417 noncoding RNAs were identified, including 109 miRNAs, 1,544 tRNAs, 4,690 snRNAs, and 10,074 rRNAs (Supplementary Table S9).

**Figure 3:**
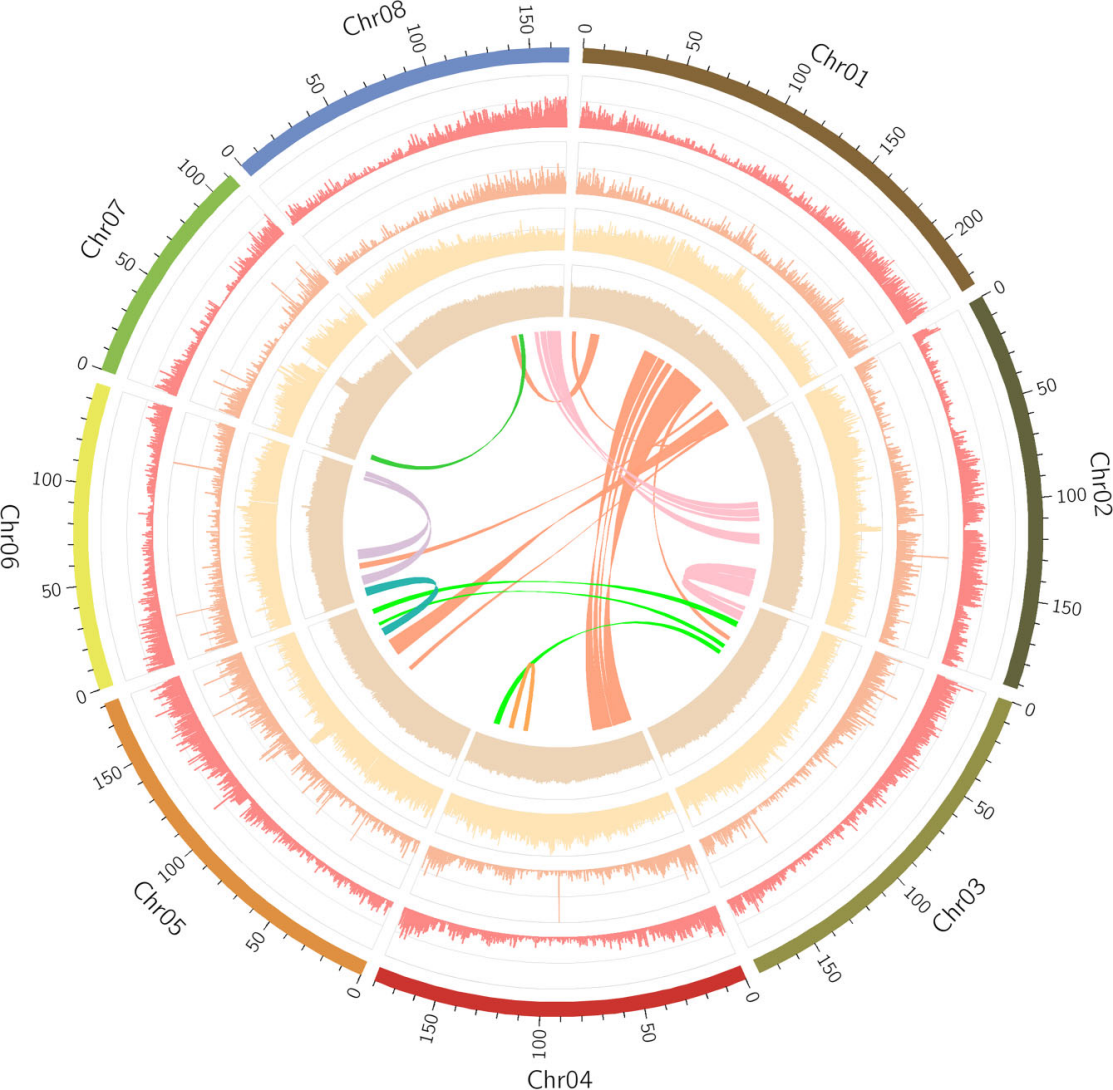
Circos plot of the assembled AM-T2T genome. Circular tracks from outside to inside indicate the pseudomolecules; gene density; gene length; repetitive density; GC content. The links in the center show syntenic region found in each chromosome.

The genome harbored 32,600 coding genes with an average CDS length of 1,169.09 bp (Fig. 3; Table 1; Supplementary Table S10). BUSCO analysis indicated that 99.07% of core conserved plant orthologs were fully detected in the AM-T2T genome (Supplementary Table S11). This completeness level surpassed the metrics observed in the AM-CLR (96.59%) and AM-ONT (97.27%) assemblies. The length distributions of messenger RNA, CDS, exons, and introns among related species supported the reliability of the annotation results (Supplementary Fig. S2). Functional annotation assigned 98.34% of coding genes to public databases, validating prediction accuracy (Supplementary Table S12). Furthermore, 24,181 (74.17%) genes showed detectable transcriptional activity (FPKM ≥ 1) (Supplementary Fig. S3). Notably, 898 genes were annotated within PURs. GO enrichment analysis revealed that these PUR-associated genes were enriched in essential biological processes, such as “zinc ion binding,” “nucleic acid binding,” “ADP binding,” and more (Supplementary Fig. S4). Among these, 452 PUR genes showed expression (FPKM ≥ 1) in at least 1 sample (Supplementary Table S13). Genome-wide prediction identified 2,187 TFs across 58 types, exceeding the count in the AM-CLR genome (2,048) [72] (Supplementary Fig. S5). Significantly, 4 CYP450 genes (*CYP71B38, KAO2, CYP71A13*, and *CYP86A1*) localized within the PURs. These findings affirmed the completeness and accuracy of gene prediction in the AM-T2T assembly.

### The characteristics of centromeric regions

The centromeric region of the genome presents a notable assembly challenge due to its high content of repetitive sequences [73]. So far, the centromeric sequence of the *A. membranaceus* genome has not been fully characterized, and our new T2T genome allows deeper exploration of the repeats in these regions. In the AM-T2T genome, centromeric sequences of all 8 pseudochromosomes were assembled, with an average length of 2,949,226 bp (Table 2). The longest centromeric region, located on pseudochromosome 1, spanned 6,931,092 bp, while the shortest, on pseudochromosome 6, measured 351,503 bp. On average, centromere sequences contained 88.63% repeat sequences, with the primary transposable elements (TEs) being DNA transposons and LTRs. Notably, TE distribution within centromeric regions varied across chromosomes: LTRs predominated in pseudochromosome 1, whereas DNA transposons were prevalent in pseudochromosomes 3, 4, 5, and 6. In addition, the average content of tandem repeats is 54.49%, which is much higher than that in the whole genome (8.43%). These results are consistent with previous reports highlighting centromeric enrichment of retrotransposons and tandem repeats [74]. Within the chromosome centromeres of AM-T2T, a total of 169 genes were identified. Function enrichment analysis showed that these genes were significantly enrichment in multiple GO terms, such as “nucleic acid binding,” “RNA-DNA hybrid ribonuclease activity,” and “chitinase activity,” suggesting their potential functions in the segregation of homologous chromosomes (Supplementary Fig. S6). Among these centromeric genes, 99 (58.58%) were expressed with an FPKM value greater than 1, a proportion slightly lower than that of all annotated genes (74.17%).

**Table 2:** The characteristic of centromeric regions of the AM-T2T assembly

Chromosome	Start	End	GC content	Gene number	Repetitive sequence content (%)	Tandem repeats content (%)	DNA transposons content (%)	LTR content (%)
Chr01	12,32,45,059	13,01,76,150	37.0%	57	84.92	34.07	1.57	77.50
Chr02	3,04,56,263	3,17,81,639	39.7%	11	84.75	51.33	47.81	36.66
Chr03	12,44,00,000	12,51,80,000	39.3%	0	97.94	85.02	72.89	23.65
Chr04	9,37,80,950	9,62,86,216	38.7%	18	92.76	57.10	53.21	34.99
Chr05	5,43,22,074	5,56,22,641	39.3%	10	84.19	49.08	50.17	35.48
Chr06	8,62,61,760	8,66,13,262	39.2%	1	96.62	77.58	65.91	26.60
Chr07	4,38,29,593	4,79,89,591	39.8%	38	84.12	43.91	39.80	44.46
Chr08	5,18,40,000	5,80,80,000	40.1%	34	83.78	37.84	31.06	51.25

### The identification of CYP450s and UGTs

**Figure 4:**
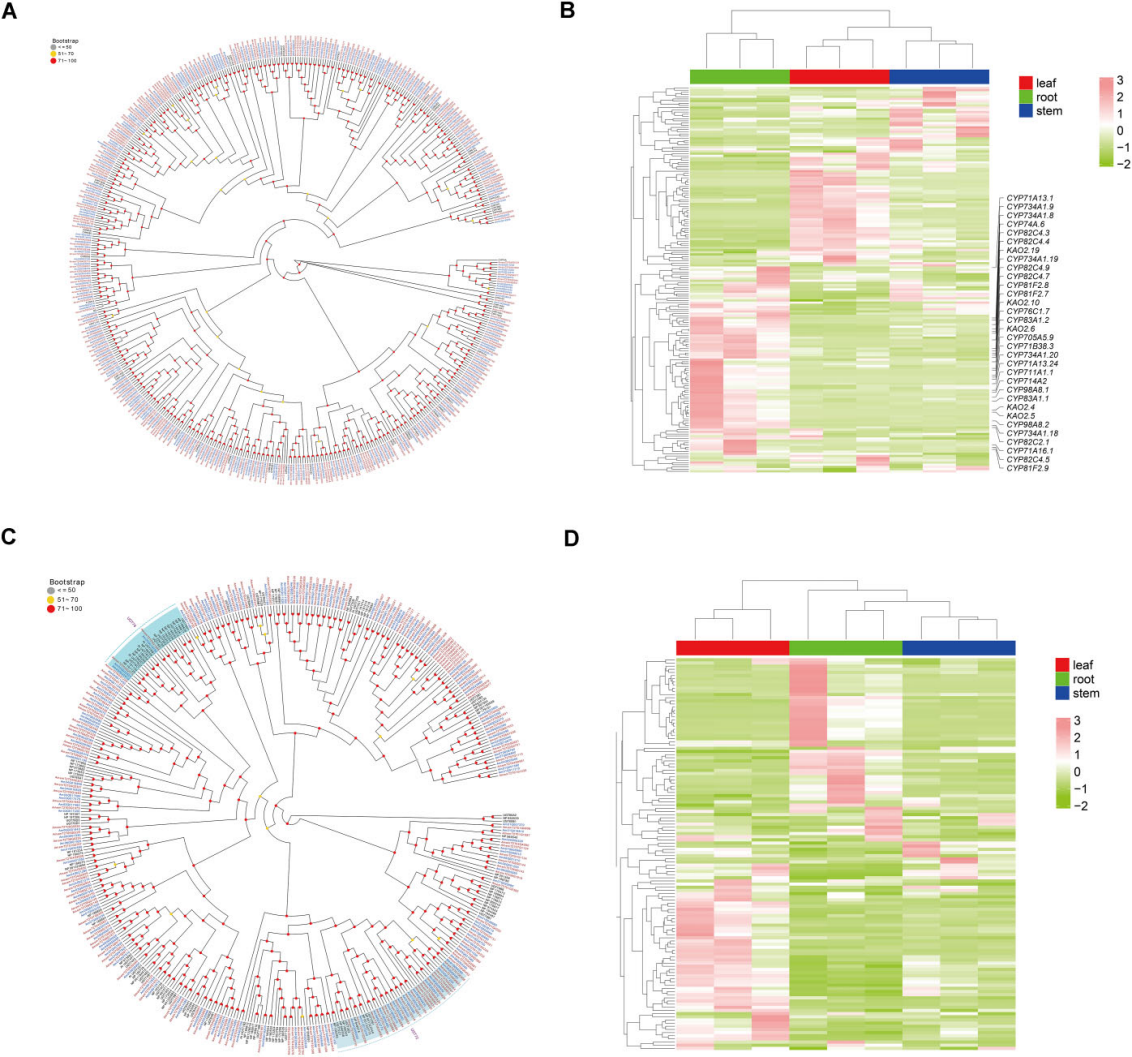
The identification of CYP450s and UGTs. (A) The phylogenetic tree of CYP450s. (B) The heatmap of CYP450s. (C) The phylogenetic tree of UGTs. (D) The heatmap of UGTs. Genes with IDs highlighted in brown represent those originating from AM-T2T; genes with IDs highlighted in blue denote genes from AMM, and those in black denote genes from *A. thaliana*.

A total of 248 and 236 CYP450 genes were identified in the AM-T2T and AMM genomes, respectively (Fig. 4A). Transcriptomic analysis revealed that 176 (70.97%) of these genes in AM-T2T were transcriptionally active (FPKM ≥ 1) in root, stem, or leaf tissues (Fig. 4B). Further, 32 CYP450 genes exhibited high expression (FPKM ≥ 10) specifically in roots and were identified as Differential gene expression (DEGs) in both root vs. stem and root vs. leaf comparisons. Six of these 32 DEGs belonged to the CYP71 subfamily.​​ Notably, 2 CYP71 genes (*CYP71B38* and *CYP71A13*) were localized to the PUR region with *CYP71B38* in centromeric regions. Previous studies suggested that CYP71 subfamily genes in *Panax ginseng* participate in the biosynthesis of secondary metabolites, aldehydes, and flavonoids [75]. Moreover, *CYP71D756* may participate in the biosynthesis of astragaloside IV in *Astragalus* genus plants [16].

For UGT genes, 163 and 149 members were detected in the AM-T2T and AMM genomes, respectively (Fig. 4C). Phylogenetic analysis revealed a significant contraction of the *UGT76* subfamily in both AM (2 genes) and AMM (2 genes) relative to *A. thaliana* (22 genes; Fig. 4C). By contrast, the *UGT73* subfamily showed significant expansion in AM (21 genes) and AMM (15 genes) compared to *A. thaliana* (9 genes). Moreover, 124 UGT genes (76.07%) in AM-T2T showed expression (FPKM ≥ 1) in root, stem, or leaf tissues (Fig. 4D). This expression ratio (76.07%) was higher than that of CYP450 genes (70.97%) and exceeded the genome-wide expression rate (74.17%). Furthermore, the average expression level of UGT genes was highest in root tissues compared to stem and leaf tissues, suggesting a potentially critical functional role for UGT genes in *A. membranaceus* development, with particularly prominent activity in roots.

### Unique genes and conserved genes

**Figure 5:**
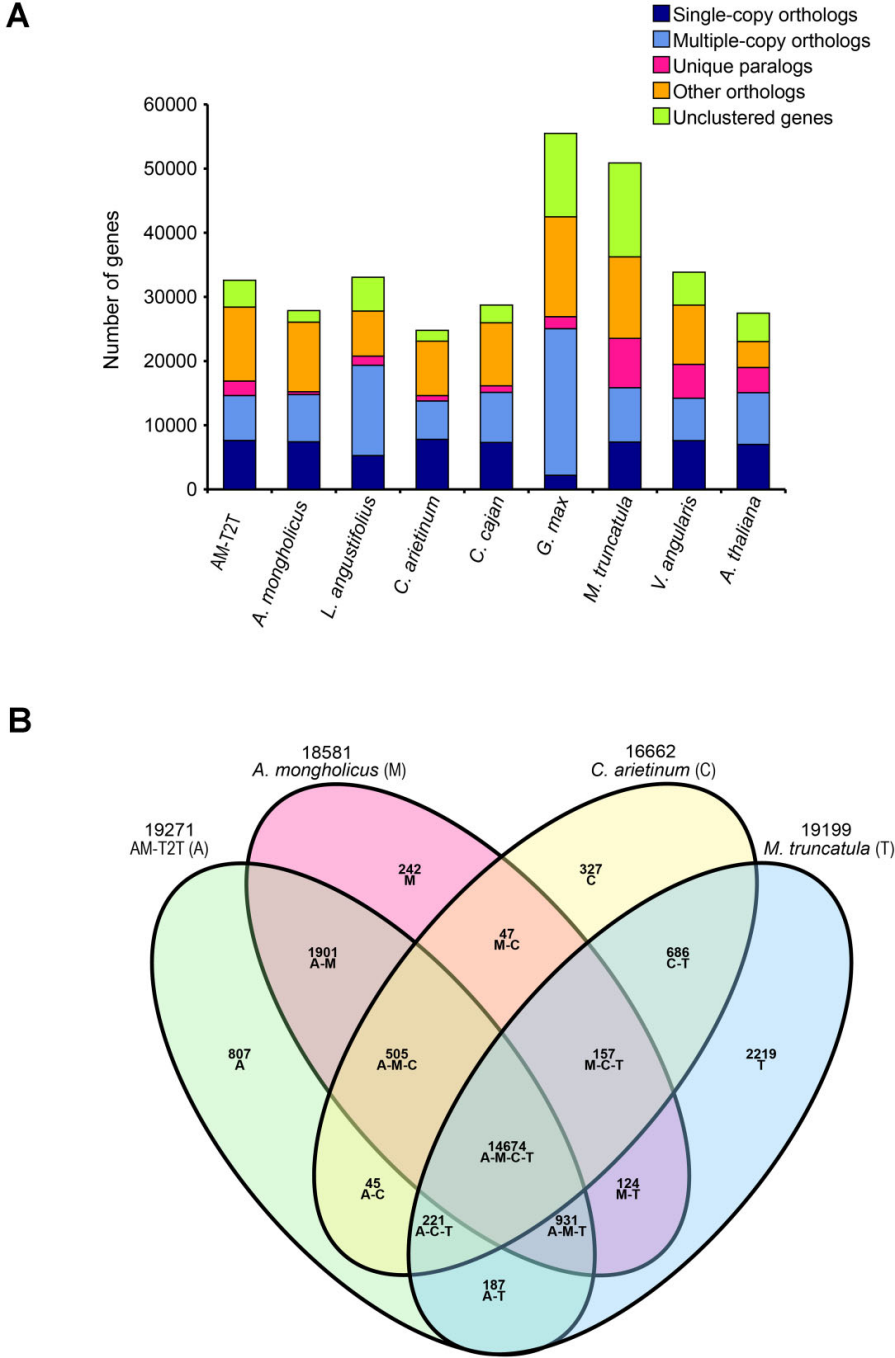
Evolution of AM-T2T genome. (A) Number of orthologous genes in 9 species. (B) Venn diagram of orthologous gene families in 4 genomes. The numbers represent quantities of gene families.

To investigate unique genes and conserved genes between the AM-T2T genome and other embryophyta, we selected 9 species for gene family construction and phylogenomic tree inference. The statistical analysis of gene family identification results showed that a total of 28,023 gene families were identified, including 9,991 shared across all 9 species. The AM-T2T genome harbored 169 unique gene families comprising 2,267 genes (Fig. 5A; Supplementary Table S14). Among these unique genes, 2,045 (90.21%) genes were supported by functional annotation, and 1,128 (49.76%) showed detectable expression (FPKM ≥ 1) in at least 1 sample (Supplementary Table S15). Expressed unique genes were significantly enriched in 34 GO terms, such as “nucleic acid binding,” “DNA binding,” “RNA-DNA hybrid ribonuclease activity,” “zinc ion binding,” and “translation,” among others (Supplementary Fig. S7). Notably, 47 TFs were identified among these expressed unique genes, implying a potential role in transcriptional regulation influencing the physiological traits of *A. membranaceus*. ​Future studies of these unique genes might dissect the regulatory genes and transcriptional networks underlying saponin/flavonoid biosynthesis via targeted mechanistic approaches.

A phylogenomic tree constructed from 1,153 single-copy genes showed that *A. membranaceus, A. mongholicus, C. arietinum*, and *M. truncatula* clustered within a major branch (Supplementary Fig. S8). Among the identified 19,271 gene families in the AM-T2T assembly, 14,674 (76.14%) resided in this clade, encompassing 20,652 genes (Fig. 5B). These genes exhibited enrichment in 44 GO terms, with “ATP binding” (GO:0005524) being the most significant (Supplementary Fig. S9).

### Whole-genome comparative analysis between AM-T2T and AMM

**Figure 6:**
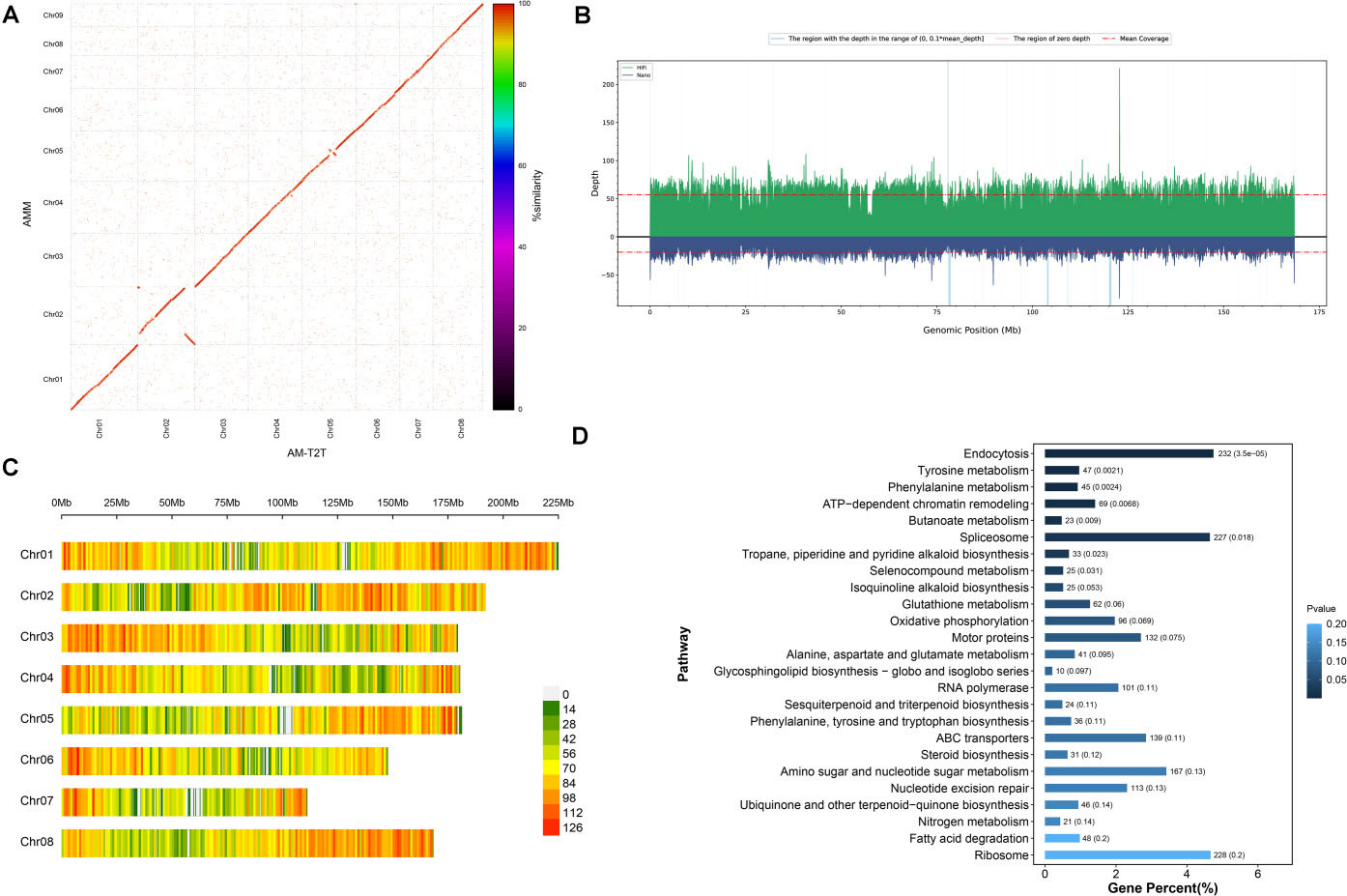
Whole-genome comparative analysis of between AM-T2T and AMM. (A) Comparison of sequence synteny between AM-T2T and AMM. (B) The filtered depth of PacBio HiFi and ONT reads across pseudochromosome 8. (C) The density plot of SVs between AM-T2T and AMM. (D) KEGG enrichment analysis of the SV genes.

Genome-wide comparative analysis of the AM-T2T and AMM genomes revealed that AM-T2T has a haploid pseudochromosome 8 (Fig. 6A), supported by the following lines of evidence: (i) Strong collinearity of pseudochromosome 8 across the AM-T2T, AM-CLR, and AM-ONT genomes indicates a low likelihood of assembly artifacts for this chromosome (Supplementary Fig. S10; Supplementary Fig. S11). (ii) Hi-C signal confirms the accuracy of the pseudochromosome 8 assembly (Fig. 2A). (iii) Comprehensive coverage of this region by both HiFi and ONT reads was observed (Fig. 6B). Additionally, this region has not been classified as low-confidence by CRAQ. The fusion region on pseudochromosome 8 was Chr08:91633450–91664996, which is composed of 91.51% repetitive sequences. Such a high proportion of repetitive sequences presents significant challenges for genome assembly. Future studies should explore the underlying mechanisms of these fusion events, their evolutionary timing, and their impacts on phenotypic traits, which represent important avenues for further investigation.

High-quality genome assemblies enabled comprehensive SV analysis. A total of 95,458 SVs were identified, including 40,764 insertions and 54,694 deletions (Fig. 6C). Among these, 42,397 (44.41%) resided within potential expression regulatory domains or CDSs of reference genes, herein defined as “SV genes.” Functional enrichment analysis revealed that these SV genes were significantly associated with the following top 10 pathways: endocytosis; tyrosine metabolism; phenylalanine metabolism; ATP-dependent chromatin remodeling; butanoate metabolism; spliceosome; tropane, piperidine, and pyridine alkaloid biosynthesis; selenocompound metabolism; isoquinoline alkaloid biosynthesis; and glutathione metabolism (Fig. 6D). The tyrosine metabolism pathway serves as a pivotal starting point for the biosynthesis of structurally diverse natural products in plants, while phenylalanine metabolism in soybean has been linked to phenylalanine ammonia-lyase, an enzyme critical for plant development and environmental stress responses [76, 77]. Notably, the SV genes harbored 97 CYP450s and 51 UGTs. Collectively, these genomic variations provide a comprehensive resource for future fundamental and applied research on *A. membranaceus*.

## Conclusions

This study presents the first high-quality telomere-to-telomere genome assembly of *A. membranaceus*, generated using PacBio HiFi reads, ONT sequencing, and Hi-C technologies. The assembled genome spans 1.39 Gb, encompassing 16 telomeres and 8 centromeres distributed across 8 chromosomes. The high quality of the assembly was verified by a 100% mapping rate of PacBio HiFi reads, 99.63% BUSCO completeness, higher RNA-seq mapping rates, an LAI of 22.67, a QV of 57.51, and a GCI score of 36.23. Genome annotation revealed 64.22% repetitive sequences, 32,600 protein-coding genes, 248 CYP450s, 163 UGTs, and 169 centromeric genes. Additionally, 158.58 Mb of PURs, 2,267 unique genes, and 20,652 conserved genes were identified. Genome-wide comparative analysis of the AM-T2T and AMM genomes revealed that AM-T2T has a haploid pseudochromosome 8. This study demonstrates the utility of advanced sequencing technologies in resolving complex genomic regions and provides a more accurate foundation for genetic research of *A. membranaceus*.

## Abbreviations

BLAST: Basic Local Alignment Search Tool; BUSCO: Benchmarking Universal Single-Copy Orthologs; CLR: continuous long read; CRAQ: Clipping information for Revealing Assembly Quality; CYP450s: cytochrome P450 monooxygenases; FPKM: fragments per kilobase of transcript per million mapped reads; Gb: gigabase pairs; GCI: Genome Continuity Inspector; GO: Gene Ontology; Hi-C: High-Throughput Chromosome Conformation Capture; HiFi: High-Fidelity; IGV: Integrative Genomics Viewer; LAI: LTR assembly index; LTR: long terminal repeat; Mb: megabase pairs; miRNAs: microRNAs; NCBI: National Center for Biotechnology Information; ncRNA: noncoding RNA; NR: NCBI’s nonredundant database; PASA: Program to Assemble Spliced Alignments; PUR: previously unassembled region; QV: quality value; RNA-seq: RNA sequencing; rRNA: ribosomal RNA; snRNA: small nuclear RNA; T2T: telomere-to-telomere; tRNA: transfer RNAs; UGT: uridine diphosphate glycosyltransferase.

## Additional Files

**Supplementary Fig. S1**. The pipelines overview of AM-T2T assembly.

**Supplementary Fig. S2**. Distribution of the gene components in the AM-T2T assembly. Window refers to the length of every point. No obvious unexpected differences exist among these 3 organisms, indicating the high quality of gene structure annotation. AMM, *Astragalus membranaceus* var. mongholicus.

**Supplementary Fig. S3**. Statistical histogram of gene expression numbers.

**Supplementary Fig. S4**. GO enrichment analysis of 898 PUR-associated genes. Gene ratio (x-axis) is the percentage of the number of genes present in this GO term over the total number of genes in this category. Larger size of a GO term represents a higher gene number.

**Supplementary Fig. S5**. Statistical chart of transcription factor family distribution in AM-T2T assembly.

**Supplementary Fig. S6**. GO enrichment analysis of 169 genes within centromeres. Gene ratio (x-axis) is the percentage of the number of genes present in this GO term over the total number of genes in this category. Larger size of a GO term represents a higher gene number.

**Supplementary Fig. S7**. GO enrichment analysis of 1128 AM-T2T specific genes with FPKM ≥1. Gene ratio (x-axis) is the percentage of the number of genes present in this GO term over the total number of genes in this category. Larger size of a GO term represents a higher gene number.

**Supplementary Fig. S8**. Phylogenetic tree of the 9 species.

**Supplementary Fig. S9**. GO enrichment analysis of 20,652 AM-T2T core genes. Gene ratio (x-axis) is the percentage of the number of genes present in this GO term over the total number of genes in this category. Larger size of a GO term represents a higher gene number.

**Supplementary Fig. S10**. Comparison of sequence synteny between AM-T2T and AM-CLR.

**Supplementary Fig. S11**. Comparison of sequence synteny between AM-T2T and AM-ONT.

**Supplementary Table S1**. Summary of newly generated whole-genome sequencing data used in this study.

**Supplementary Table S2**. The statistics of the contig assembly.

**Supplementary Table S3**. The statistics of the anchored chromosome length.

**Supplementary Table S4**. The low confidence region within the AM-T2T assembly.

**Supplementary Table S5**. Summary of the RNA-seq reads genome mapping rate.

**Supplementary Table S6**. The identification of PUR region in the AM-T2T assembly.

**Supplementary Table S7**. General statistics of repeats in the AM-T2T assembly.

**Supplementary Table S8**. The summary of interspersed repeat contents in the AM-T2T assembly.

**Supplementary Table S9**. Annotation of ncRNA in the AM-T2T assembly.

**Supplementary Table S10**. The length statistics of genes in the AM-T2T assembly.

**Supplementary Table S11**. BUSCO analysis of AM-T2T gene set completeness.

**Supplementary Table S12**. Number of functional annotations for predicted genes in the AM-T2T assembly.

**Supplementary Table S13**. The gene list in the PUR region of the AM-T2T assembly.

**Supplementary Table S14**. Gene families in AM-T2T and other species.

**Supplementary Table S15**. The list of AM-T2T unique genes.

giaf117_Supplemental_Files

giaf117_Authors_Response_To_Reviewer_Comments_Original_Submission

giaf117_GIGA-D-25-00123_Original_Submission

giaf117_GIGA-D-25-00123_Revision_1

giaf117_Reviewer_1_Report_Original_Submission Li Wang -- 5/7/2025

giaf117_Reviewer_1_Report_Revision_1 Li Wang -- 8/4/2025

giaf117_Reviewer_2_Report_Original_Submission Wei Sun -- 5/15/2025

giaf117_Reviewer_2_Report_Revision_1 Wei Sun -- 8/3/2025

## Data Availability

The raw sequencing data that support the findings of this study have been deposited into NCBI with accession number SRA: SRR35052312 and EBI with accession number ENA: ERR15401760. All additional supporting data are available in the *GigaScience* repository, GigaDB [78].

## References

[1] Fu J, Wang Z, Huang L, et al. Review of the botanical characteristics, phytochemistry, and pharmacology of Astragalus membranaceus (Huangqi). Phytother Res. 2014;28(9):1275–83. 10.1002/ptr.5188. 25087616

[2] Auyeung KK, Han Q-B, Ko JK. Astragalus membranaceus: a review of its protection against inflammation and gastrointestinal cancers. Am J Chin Med. 2016;44(1):1–22. 10.1142/S0192415X16500014. 26916911

[3] Li C-X, Liu Y, Zhang Y-Z, et al. Astragalus polysaccharide: a review of its immunomodulatory effect. Arch Pharm Res. 2022;45(6):367–89. 10.1007/s12272-022-01393-3. 35713852

[4] Chen J, Wu X-T, Xu Y-Q, et al. Global transcriptome analysis profiles metabolic pathways in traditional herb Astragalus membranaceus Bge. Var. Mongolicus (Bge.) Hsiao. BMC Genomics. 2015;16(Suppl 7):S15. 10.1186/1471-2164-16-S7-S15. PMC4474414 26099797

[5] Kim Y, Thwe A, Li X, et al. Accumulation of astragalosides and related gene expression in different organs of Astragalus membranaceus Bge. Var mongholicus (Bge.). Molecules. 2014;19:10922–35. 10.3390/molecules190810922. 25068786 PMC6270750

[6] Kim YB, Thwe AA, Li X, et al. Accumulation of flavonoids and related gene expressions in different organs of Astragalus membranaceus Bge. Appl Biochem Biotechnol. 2014;173(8):2076–85. 10.1007/s12010-014-1004-1. 24903957

[7] Yu M, Zhao J, Shan Y, et al. Genome-wide DNA methylation analysis of Astragalus on the intervention of ID2 promoter via PI3K/Akt signaling pathway in peritoneal fibrosis. Sci Rep. 2025;15(1):15786. 10.1038/s41598-025-96709-w. 40328830 PMC12056223

[8] Xu W-X, Liu Z-Y, Bu Q-Y, et al. Eight new triterpenoid saponins from the leaves of Astragalus membranaceus (Fisch.) Bunge and their neuroprotective effects. Fitoterapia. 2025;183:106559. 10.1016/j.fitote.2025.106559. 40258427

[9] Xiao S, Liu L, Qin X, et al. Astragenol alleviates neuroinflammation and improves Parkinson’s symptoms through amino acid metabolism pathway and inhibition of ferroptosis. J Ethnopharmacol. 2025;348:119896. 10.1016/j.jep.2025.119896. 40306495

[10] Zhang K, Qu G, Zhang Y, et al. Assembly and comparative analysis of the first complete mitochondrial genome of astragalus membranaceus (Fisch.) Bunge: an invaluable traditional Chinese medicine. BMC Plant Biol. 2024;24(1):1055. 10.1186/s12870-024-05780-4. 39511474 PMC11546474

[11] Li J, Harata-Lee Y, Denton MD, et al. Long read reference genome-free reconstruction of a full-length transcriptome from Astragalus membranaceus reveals transcript variants involved in bioactive compound biosynthesis. Cell Discov. 2017;3(1):17031. 10.1038/celldisc.2017.31. 28861277 PMC5573880

[12] Wang J, Rong Z, Shi W, et al. Characterization of the WRKY family transcription factors in Astragalus membranaceus and their expression under drought stress. BMC Plant Biol. 2025;25(1):593. 10.1186/s12870-025-06592-w. 40329159 PMC12054254

[13] Qi L, Sun H, Tian C, et al. The AmMYB35-AmFLS module mediates the accumulation of flavonol induced by drought stress in astragalus membranaceus. Food Biosci. 2025;68:106541. 10.1016/j.fbio.2025.106541.

[14] Hwang C, Yan S, Choe Y, et al. Efficient hairy root induction system of astragalus membranaceus and significant enhancement of astragalosides via overexpressing AmUGT15. Plant Cell Rep. 2024;43(12):285. 10.1007/s00299-024-03370-0. 39560736

[15] Fan H, Chai Z, Yang X, et al. Chromosome-scale genome assembly of astragalus membranaceus using PacBio and Hi-C technologies. Sci Data. 2024;11(1):1071. 10.1038/s41597-024-03852-6. 39358417 PMC11446949

[16] Xu B, Huang J-P, Peng G, et al. Total biosynthesis of the medicinal triterpenoid saponin astragalosides. Nat Plants. 2024;10:1826–37. 10.1038/s41477-024-01827-4. 39433972

[17] Chen Y, Fang T, Su H, et al. A reference-grade genome assembly for Astragalus mongholicus and insights into the biosynthesis and high accumulation of triterpenoids and flavonoids in its roots. Plant Commun. 2022;4(2):100469. 10.1016/j.xplc.2022.100469 36307985 PMC10030368

[18] Shang L, He W, Wang T, et al. A complete assembly of the rice Nipponbare reference genome. Mol Plant. 2023;16(8):1232–36. 10.1016/j.molp.2023.08.003. 37553831

[19] Chen J, Wang Z, Tan K, et al. A complete telomere-to-telomere assembly of the maize genome. Nat Genet. 2023;55(7):1221–31. 10.1038/s41588-023-01419-6. 37322109 PMC10335936

[20] Li M, Chen C, Wang H, et al. Telomere-to-telomere genome assembly of sorghum. Sci Data. 2024;11:835. 10.1038/s41597-024-03664-8. 39095379 PMC11297213

[21] Li H, Durbin R. Genome assembly in the telomere-to-telomere era. Nat Rev Genet. 2024;25(9):658–70. 10.1038/s41576-024-00718-w. 38649458

[22] Garg V, Bohra A, Mascher M, et al. Unlocking plant genetics with telomere-to-telomere genome assemblies. Nat Genet. 2024;56:1788–99. 10.1038/s41588-024-01830-7. 39048791

[23] De Coster W, D’Hert S, Schultz DT, et al. NanoPack: visualizing and processing long-read sequencing data. Bioinformatics. 2018;34:2666–69. 10.1093/bioinformatics/bty149. 29547981 PMC6061794

[24] Cheng H, Concepcion GT, Feng X, et al. Haplotype-resolved de novo assembly using phased assembly graphs with hifiasm. Nat Methods. 2021;18(2):170–75. 10.1038/s41592-020-01056-5. 33526886 PMC7961889

[25] Kolmogorov M, Yuan J, Lin Y, et al. Assembly of long, error-prone reads using repeat graphs. Nat Biotechnol. 2019;37(5):540–46. 10.1038/s41587-019-0072-8. 30936562

[26] Hu J, Wang Z, Sun Z, et al. NextDenovo: an efficient error correction and accurate assembly tool for noisy long reads. Genome Biol. 2024;25(1):107. 10.1186/s13059-024-03252-4. 38671502 PMC11046930

[27] Ruan J, Li H. Fast and accurate long-read assembly with wtdbg2. Nat Methods. 2020;17(Suppl 6):1–4. 10.1038/s41592-019-0669-3 31819265 PMC7004874

[28] Zeng X, Yi Z, Zhang X, et al. Chromosome-level scaffolding of haplotype-resolved assemblies using Hi-C data without reference genomes. Nat Plants. 2024;10(8):1184–200. 10.1038/s41477-024-01755-3. 39103456

[29] Durand NC, Robinson JT, Shamim MS, et al. Juicebox provides a visualization system for Hi-C contact maps with unlimited zoom. Cell Syst. 2016;3(1):99–101. 10.1016/j.cels.2015.07.012. 27467250 PMC5596920

[30] Lin Y, Ye C, Li X, et al. quarTeT: a telomere-to-telomere toolkit for gap-free genome assembly and centromeric repeat identification. Horticult Res. 2023;10:uhad127. 10.1093/hr/uhad127. PMC10407605 37560017

[31] Xu G-C, Xu TJ, Zhu R, et al. LR_Gapcloser: a tiling path-based gap closer that uses long reads to complete genome assembly. Gigascience. 2018;8:giy157. 10.1093/gigascience/giy157 PMC6324547 30576505

[32] Jain C, Rhie A, Hansen NF, et al. Long-read mapping to repetitive reference sequences using Winnowmap2. Nat Methods. 2022;19(6):705–10. 10.1038/s41592-022-01457-8. 35365778 PMC10510034

[33] Vaser R, Sović I, Nagarajan N, et al. Fast and accurate de novo genome assembly from long uncorrected reads. Genome Res. 2017;27(5):737–46. 10.1101/gr.214270.116. 28100585 PMC5411768

[34] Seppey M, Manni M, Zdobnov EM. BUSCO: assessing genome assembly and annotation completeness. Methods Mol Biol. 2019;1962:227–45. 10.1186/s13059-020-02134-9 31020564

[35] Rhie A, Walenz BP, Koren S, et al. Merqury: reference-free quality, completeness, and phasing assessment for genome assemblies. Genome Biol. 2020;21:245. 10.1186/s13059-020-02134-9. 32928274 PMC7488777

[36] Li H . Minimap2: pairwise alignment for nucleotide sequences. Bioinformatics. 2018;34(18):3094–100. 10.1093/bioinformatics/bty191. 29750242 PMC6137996

[37] Ou S, Chen J, Jiang N. Assessing genome assembly quality using the LTR Assembly Index (LAI). Nucleic Acids Res. 2018;46(21):e126. 10.1093/nar/gky730 30107434 PMC6265445

[38] Chen Q, Yang C, Zhang G, et al. GCI: a continuity inspector for complete genome assembly. Bioinformatics. 2024;40(11):btae633. 10.1093/bioinformatics/btae633. 39432569 PMC11550331

[39] Li K, Xu P, Wang J, et al. Identification of errors in draft genome assemblies at single-nucleotide resolution for quality assessment and improvement. Nat Commun. 2023;14(1):6556. 10.1038/s41467-023-42336-w. 37848433 PMC10582259

[40] Bergman CM, Quesneville H. Discovering and detecting transposable elements in genome sequences. Briefings Bioinf. 2007;8(6):382–92. 10.1093/bib/bbm048. 17932080

[41] Xu Z, Wang H. LTR-FINDER: an efficient tool for the prediction of full-length LTR retrotransposons. Nucleic Acids Res. 2007;35:W265–68. 10.1093/nar/gkm286. 17485477 PMC1933203

[42] Benson G . Tandem repeats finder: a program to analyze DNA sequences. Nucleic Acids Res. 1999;27(2):573–80. 10.1093/nar/27.2.573. 9862982 PMC148217

[43] Grabherr MG, Haas BJ, Yassour M, et al. Full-length transcriptome assembly from RNA-seq data without a reference genome. Nat Biotechnol. 2011;29(7):644–52. 10.1038/nbt.1883. 21572440 PMC3571712

[44] Haas B . Improving the Arabidopsis genome annotation using maximal transcript alignment assemblies. Nucleic Acids Res. 2003;31:5654–66. 10.1093/nar/gkg770. 14500829 PMC206470

[45] Kim D, Langmead B, Salzberg SL. HISAT: a fast spliced aligner with low memory requirements. Nat Methods. 2015;12(4):357–60. 10.1038/nmeth.3317. 25751142 PMC4655817

[46] Kovaka S, Zimin AV, Pertea GM, et al. Transcriptome assembly from long-read RNA-seq alignments with StringTie2. Genome Biol. 2019;20(1):278. 10.1186/s13059-019-1910-1. 31842956 PMC6912988

[47] Hou X, Wang D, Cheng Z, et al. A near-complete assembly of an Arabidopsis thaliana genome. Mol Plant. 2022;15(8):1247–50. 10.1016/j.molp.2022.05.014. 35655433

[48] Zhang C, Xie L, Yu H, et al. The T2T genome assembly of soybean cultivar ZH13 and its epigenetic landscapes. Mol Plant. 2023;16(11):1715–18. 10.1016/j.molp.2023.10.003. 37803825

[49] Keilwagen J, Hartung F, Grau J. GeMoMa: homology-based gene prediction utilizing intron position conservation and RNA-seq data. Methods Mol Biol. 2019;1962:161–77. 10.1007/978-1-4939-9173-0_9 31020559

[50] Haas BJ, Salzberg SL, Zhu W, et al. Automated eukaryotic gene structure annotation using EVidenceModeler and the Program to assemble spliced alignments. Genome Biol. 2008;9(1):R7. 10.1186/gb-2008-9-1-r7. 18190707 PMC2395244

[51] Buchfink B, Xie C, Huson DH. Fast and sensitive protein alignment using DIAMOND. Nat Methods. 2015;12(1):59–60. 10.1038/nmeth.3176. 25402007

[52] Bairoch A, Apweiler R. The SWISS-PROT protein sequence data bank and its supplement TrEMBL in 1999. Nucleic Acids Res. 1999;27(1):49–54. 10.1093/nar/27.1.49. 9847139 PMC148094

[53] Kanehisa M . KEGG: Kyoto Encyclopedia of Genes and Genomes. Nucleic Acids Res. 2000;28(1):27–30. 10.1093/nar/28.1.27. 10592173 PMC102409

[54] Jones P, Binns D, Chang H-Y, et al. InterProScan 5: genome-scale protein function classification. Bioinformatics. 2014;30(9):1236–40. 10.1093/bioinformatics/btu031. 24451626 PMC3998142

[55] Wu Y-M, Li J-R, Li J, et al. Investigation of sex expression profiles and the cantharidin biosynthesis genes in two blister beetles. PLoS One. 2023;18:e0290245. 10.1371/journal.pone.0290245. 37594933 PMC10437994

[56] Langmead B, Salzberg SL. Fast gapped-read alignment with Bowtie 2. Nat Methods. 2012;9(4):357–59. 10.1038/nmeth.1923. 22388286 PMC3322381

[57] Li B, Dewey CN. RSEM: accurate transcript quantification from RNA-seq data with or without a reference genome. BMC Bioinf. 2011;12(1):323. 10.1186/1471-2105-12-323. PMC3163565 21816040

[58] Love MI, Huber W, Anders S. Moderated estimation of fold change and dispersion for RNA-seq data with DESeq2. Genome Biol. 2014;15(12):550. 10.1186/s13059-014-0550-8. 25516281 PMC4302049

[59] Li D, Qian J, Li W, et al. A high-quality genome assembly of the eggplant provides insights into the molecular basis of disease resistance and chlorogenic acid synthesis. Mol Ecol Resour. 2021;21(4):1274–86. 10.1111/1755-0998.13321. 33445226

[60] Lowe TM, Eddy SR. tRNAscan-SE: a program for improved detection of transfer RNA genes in genomic sequence. Nucleic Acids Res. 1997;25(5):955–64. 10.1093/nar/25.5.955. 9023104 PMC146525

[61] Wang H, Wang J, Chen C, et al. A complete reference genome of broomcorn millet. Sci Data. 2024;11:657. 10.1038/s41597-024-03489-5. 38906866 PMC11192726

[62] Eddy SR . Profile hidden Markov models. Bioinformatics. 1998;14(9):755–63. 10.1093/bioinformatics/14.9.755. 9918945

[63] Zhang Y, Pan X, Shi T, et al. P450Rdb: a manually curated database of reactions catalyzed by cytochrome P450 enzymes. J Adv Res. 2024;63:35–42. 10.1016/j.jare.2023.10.012. 37871773 PMC11380020

[64] Nakamura T, Yamada KD, Tomii K, et al. Parallelization of MAFFT for large-scale multiple sequence alignments. Bioinformatics. 2018;34(14):2490–92. 10.1093/bioinformatics/btq224 29506019 PMC6041967

[65] Capella-Gutiérrez S, Silla-Martínez JM, Gabaldón T. trimAl: a tool for automated alignment trimming in large-scale phylogenetic analyses. Bioinformatics. 2009;25(15):1972–73. 10.1093/bioinformatics/btp348 19505945 PMC2712344

[66] Minh BQ, Schmidt HA, Chernomor O, et al. IQ-TREE 2: new models and efficient methods for phylogenetic inference in the genomic era. Mol Biol Evol. 2020;37(5):1530–34. 10.1093/molbev/msaa015. 32011700 PMC7182206

[67] Liu Y, Wang Q, Liu X, et al. pUGTdb: a comprehensive database of plant UDP-dependent glycosyltransferases. Mol Plant. 2023;16(4):643–46. 10.1016/j.molp.2023.01.003. 36609142

[68] Marçais G, Delcher AL, Phillippy AM, et al. MUMmer4: a fast and versatile genome alignment system. PLoS Comput Biol. 2018;14: e1005944. 10.1371/journal.pcbi.1005944 29373581 PMC5802927

[69] Xie C, Mao X, Huang J, et al. KOBAS 2.0: a web server for annotation and identification of enriched pathways and diseases. Nucleic Acids Res. 2011;39(Web Server issue):W316–22. 10.1093/nar/gkr483. 21715386 PMC3125809

[70] Quan WS . Karyotype analysis of astragalus membranaceus. Hubei Agricultural Sciences. 2006;45(5):631–633. 10.14088/j.cnki.issn0439-8114.2006.05.038

[71] Hong K . Karyotype diversity of six Astragalus species. Guihaia. 2012;32(5):579–82. 10.3969/j.issn.1000-3142.2012.05.002

[72] Fan H, Chai Z, Yang X, et al. Chromosome-scale genome assembly of Astragalus membranaceus using PacBio and Hi-C technologies. Sci Data. 2024;11:1071. 10.1038/s41597-024-03852-6. 39358417 PMC11446949

[73] Deng Y, Liu S, Zhang Y, et al. A telomere-to-telomere gap-free reference genome of watermelon and its mutation library provide important resources for gene discovery and breeding. Mol Plant. 2022;15:1268–84. 10.1016/j.molp.2022.06.010. 35746868

[74] Liu Y, Liu Q, Su H, et al. Genome-wide mapping reveals R-loops associated with centromeric repeats in maize. Genome Res. 2021;31(8):1409–18. 10.1101/gr.275270.121. 34244230 PMC8327920

[75] Seitz C, Eder C, Deiml B, et al. Cloning, functional identification and sequence analysis of flavonoid 3'-hydroxylase and flavonoid 3',5'-hydroxylase cDNAs reveals independent evolution of flavonoid 3',5'-hydroxylase in the Asteraceae family. Plant Mol Biol. 2006;61(3):365–81. 10.1007/s11103-006-0012-0. 16830174

[76] Rizwan HM, He J, Arshad MB, et al. Characterization of phenylalanine ammonia-lyase genes in soybean: genomic insights and expression analysis under abiotic stress tolerance. Plant Stress. 2025;16:100896. 10.1016/j.stress.2025.100896.

[77] Xu J-J, Fang X, Li C-Y, et al. General and specialized tyrosine metabolism pathways in plants. aBIOTECH. 2020;1(2):97–105. 10.1007/s42994-019-00006-w. 36304719 PMC9590561

[78] Qin H, Li A, Zhong S, et al. Supporting data for “The Complete Genome Assembly of Astragalus membranaceus: Enabling More Accurate Genetic Research.” GigaScience Database. 2025. 10.5524/102751. PMC12486382 41032019

