# Supplementary material for: The complete genome assembly of Astragalus membranaceus: enabling more accurate genetic research
Source: Gigascience. 2025 Oct 1;14:giaf117. doi: 10.1093/gigascience/giaf117 (PMC12486382; doi:10.1093/gigascience/giaf117)
Supplement: giaf117_Supplemental_Files [file giaf117_supplemental_files.zip › Revised Supplementary Figure.docx]

**Supplementary Figures**


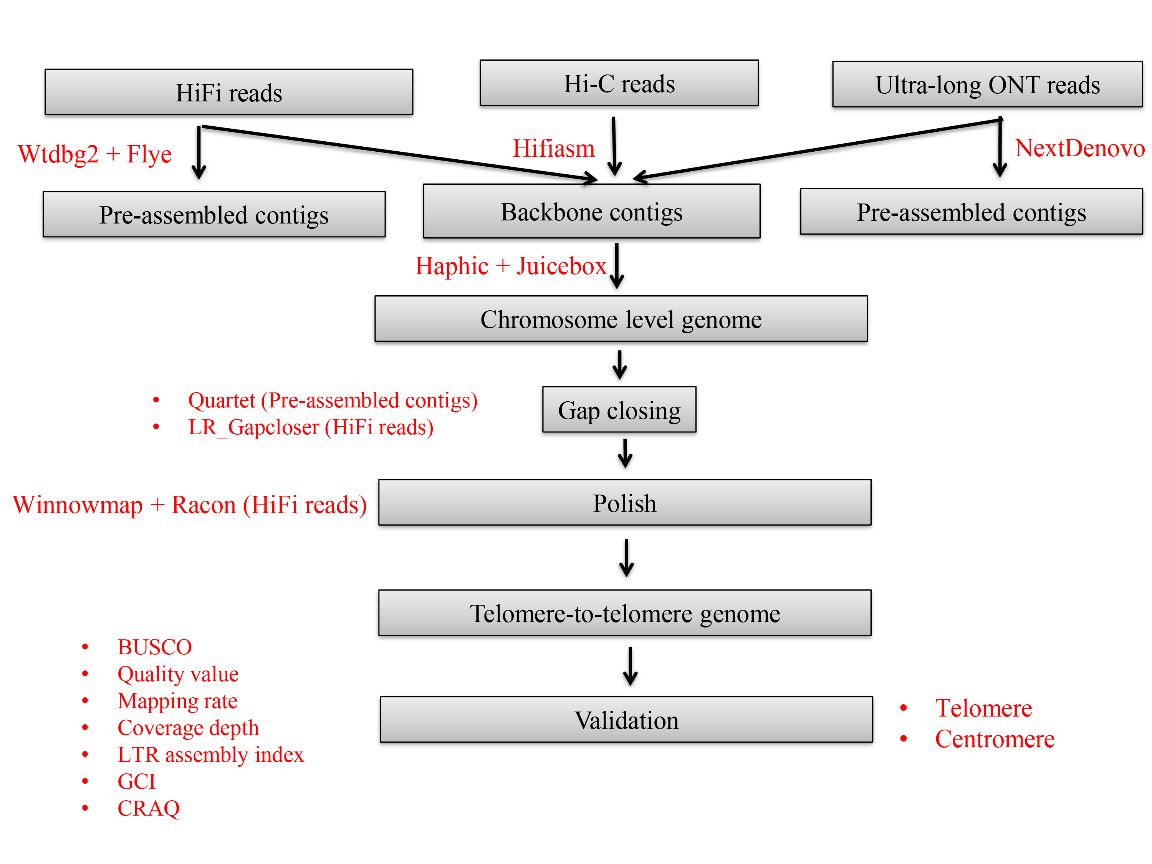


**Supplementary Fig. 1 The pipelines overview of AM-T2T assembly.**


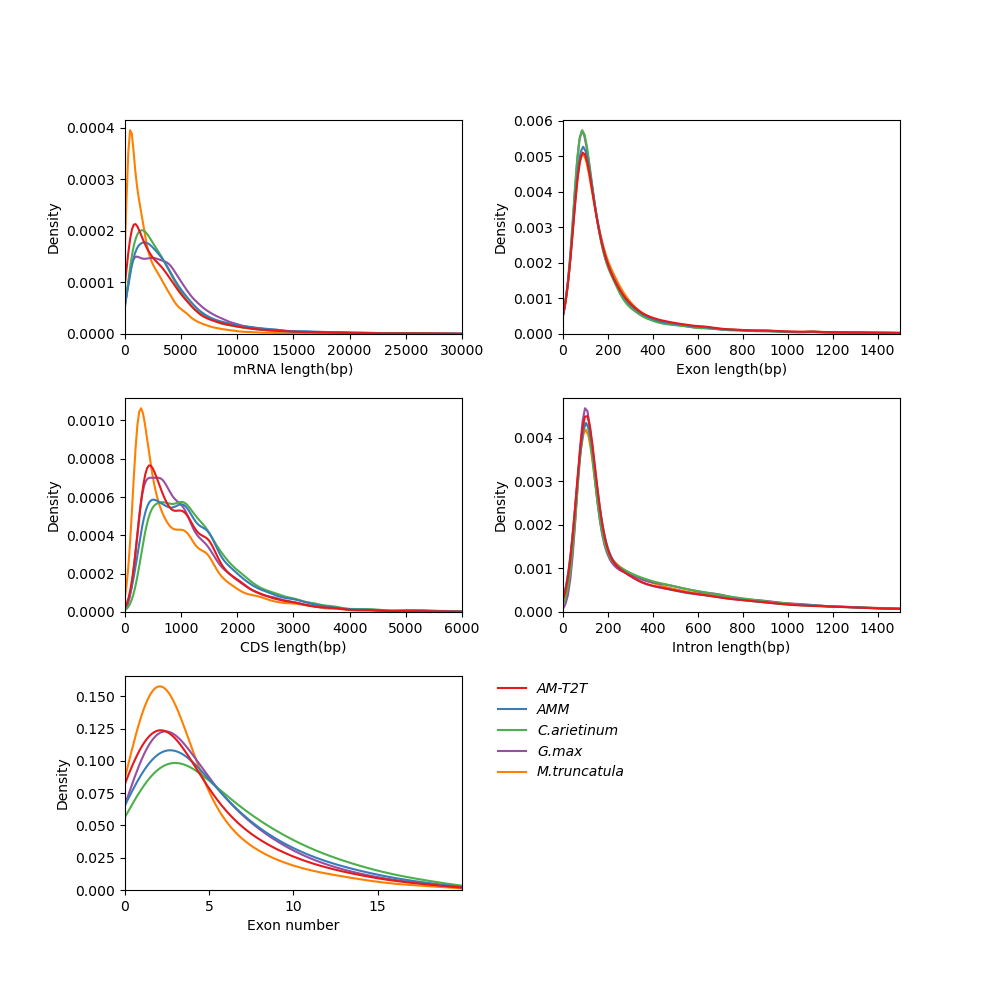


**Supplementary Fig. 2 Distribution of the gene components in the AM-T2T assembly.** Window refers to the length of every point. No obvious unexpected differences exist among these three organisms, indicating the high quality of gene structure annotation. AMM, *Astragalus membranaceus* var. mongholicus.


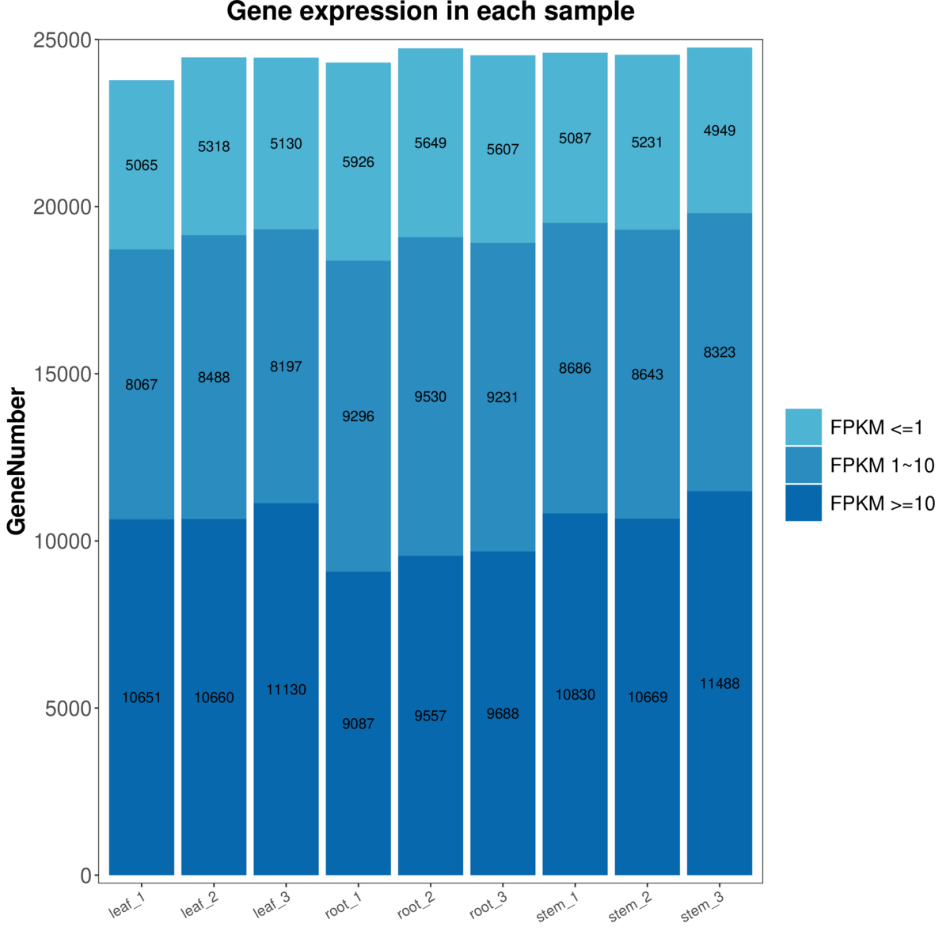


**Supplementary Fig. 3 Statistical histogram of gene expression numbers.**


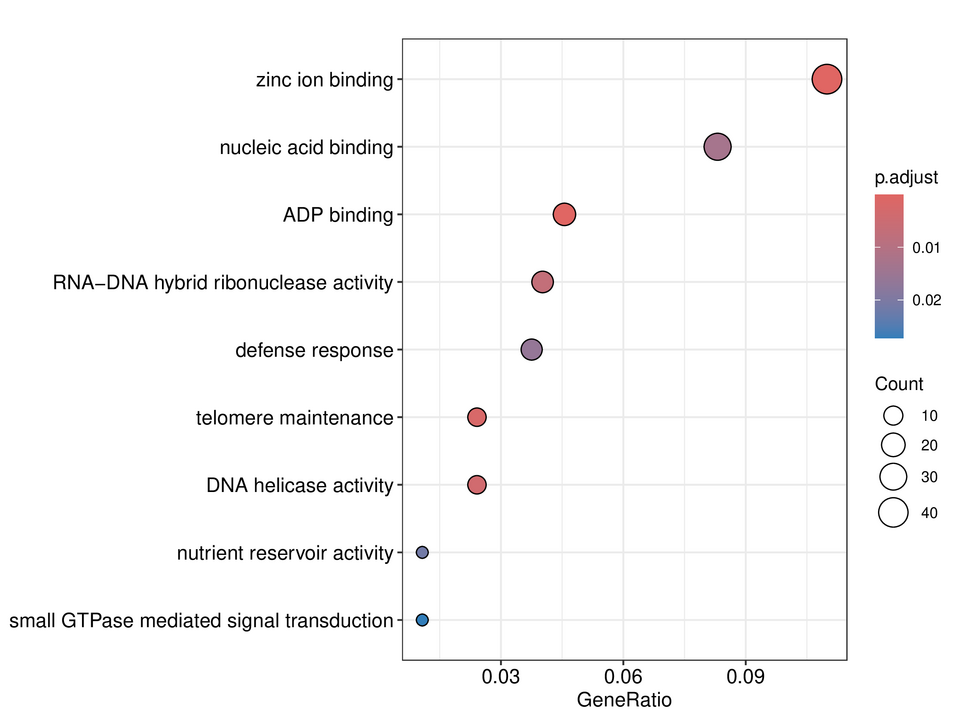


**Supplementary Fig. 4 GO enrichment analysis of 898 PUR-associated genes.** Gene ratio (x-axis) is the percentage of the number of genes present in this GO term over the total number of genes in this category. Larger size of a GO term represents a higher gene number.


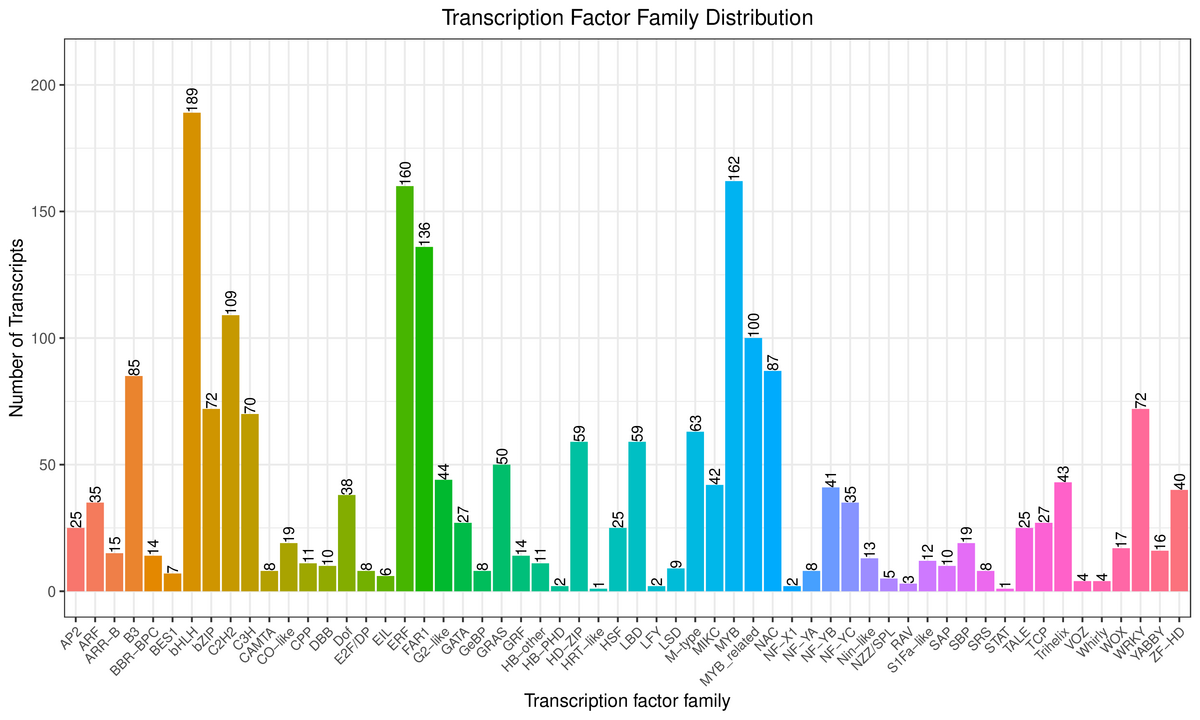


**Supplementary Fig. 5 Statistical chart of transcription factor family distribution in AM-T2T assembly.**


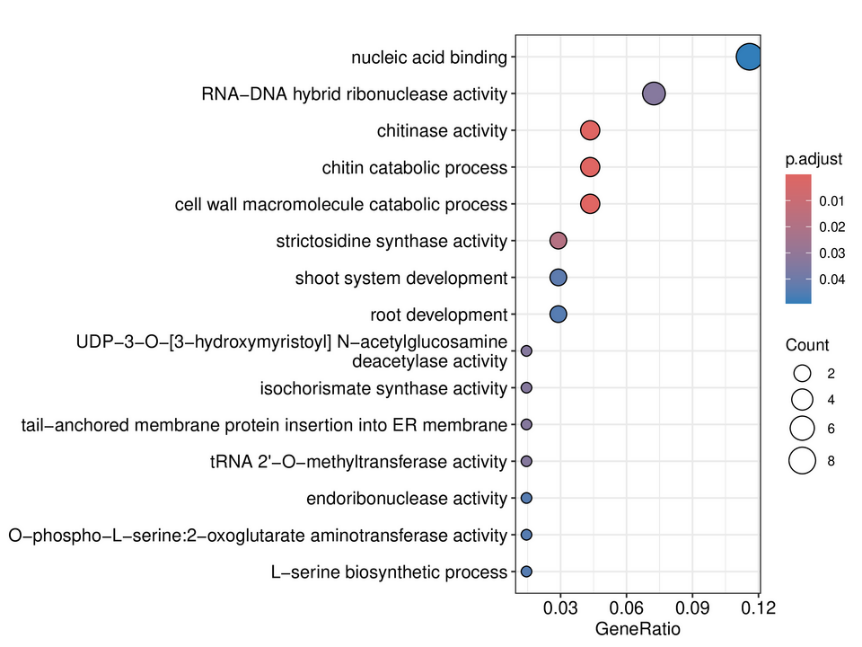


**Supplementary Fig. 6 GO enrichment analysis of 169 genes within centromeres.** Gene ratio (x-axis) is the percentage of the number of genes present in this GO term over the total number of genes in this category. Larger size of a GO term represents a higher gene number.


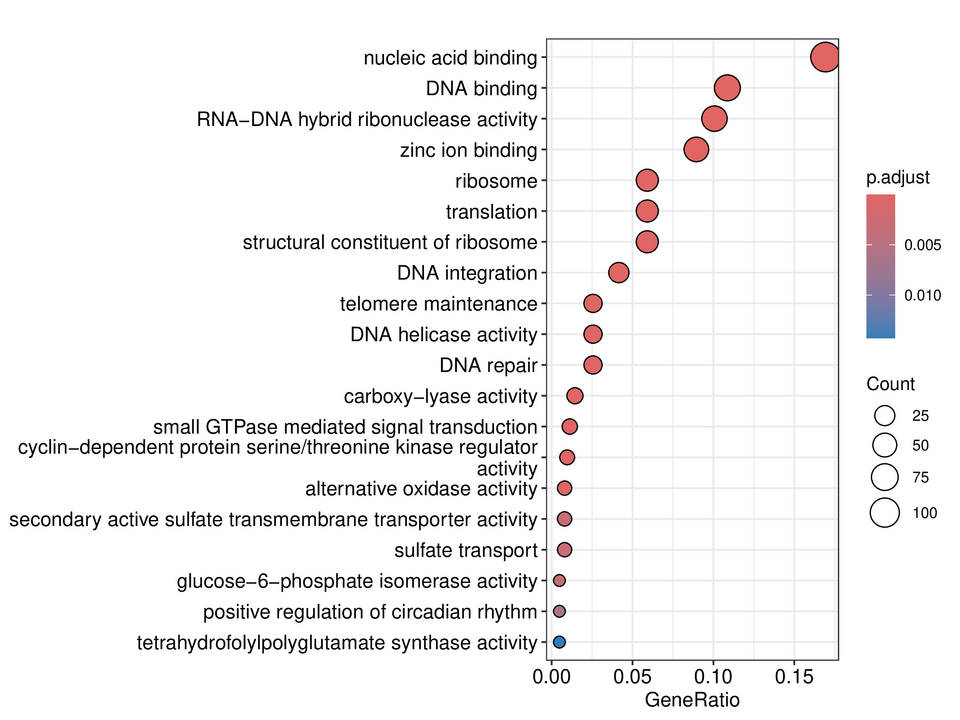


**Supplementary Fig. 7 GO enrichment analysis of 1128 AM-T2T specific genes with FPKM ≥ 1.** Gene ratio (x-axis) is the percentage of the number of genes present in this GO term over the total number of genes in this category. Larger size of a GO term represents a higher gene number.

**
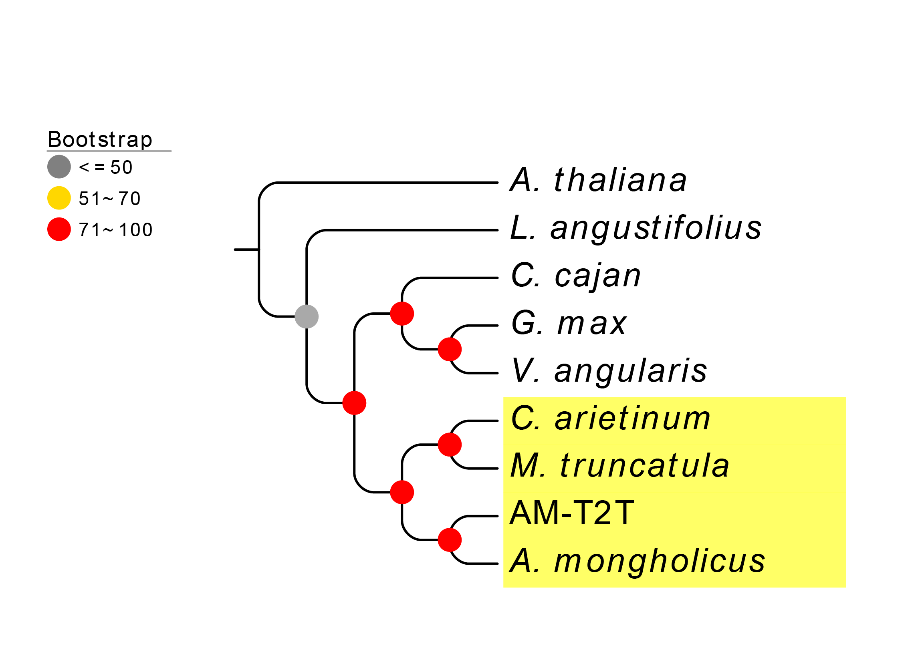
**

**Supplementary Fig. 8 Phylogenetic tree of the nine species.**

**
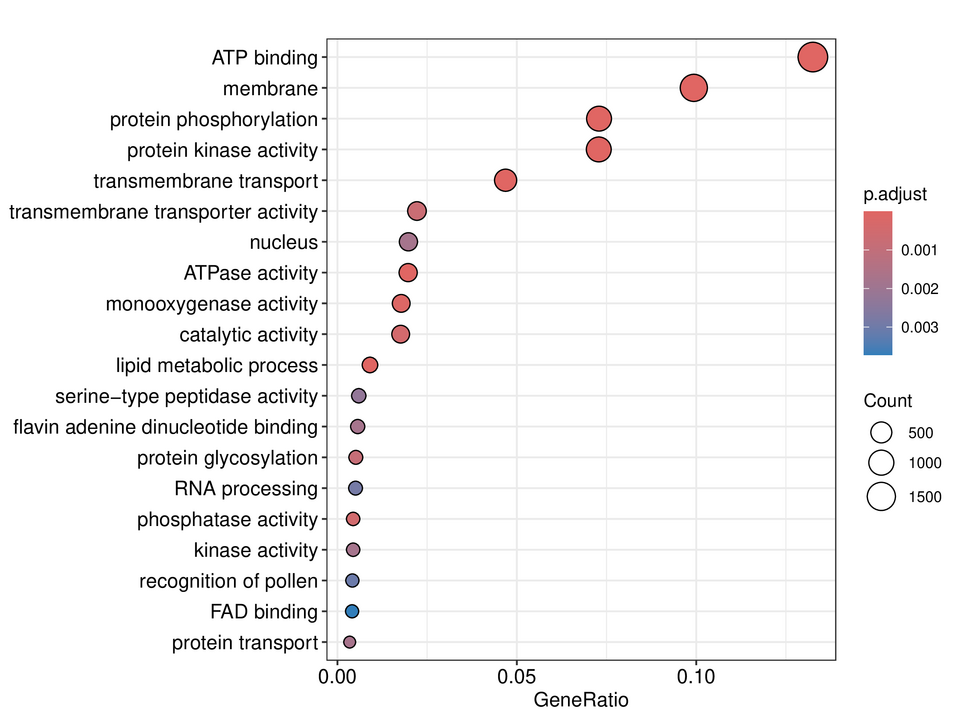
**

**Supplementary Fig. 9 GO enrichment analysis of 20,652 AM-T2T core genes.** Gene ratio (x-axis) is the percentage of the number of genes present in this GO term over the total number of genes in this category. Larger size of a GO term represents a higher gene number.

**
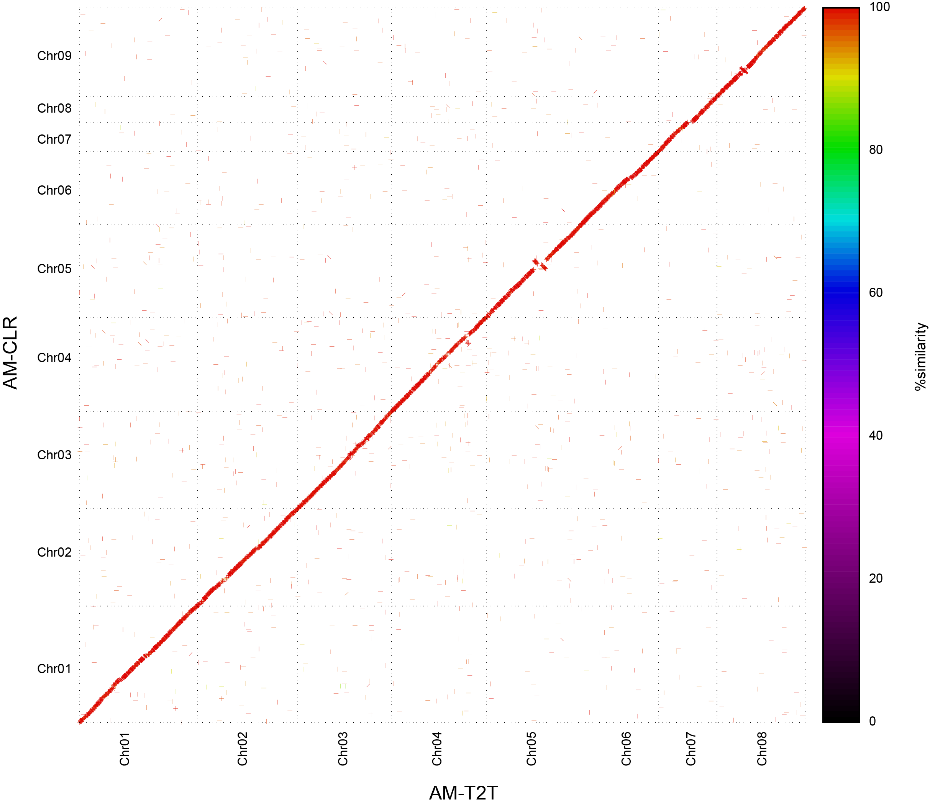
**

**Supplementary Fig. 10 Comparison of sequence synteny between AM-T2T and AM-CLR.**

**
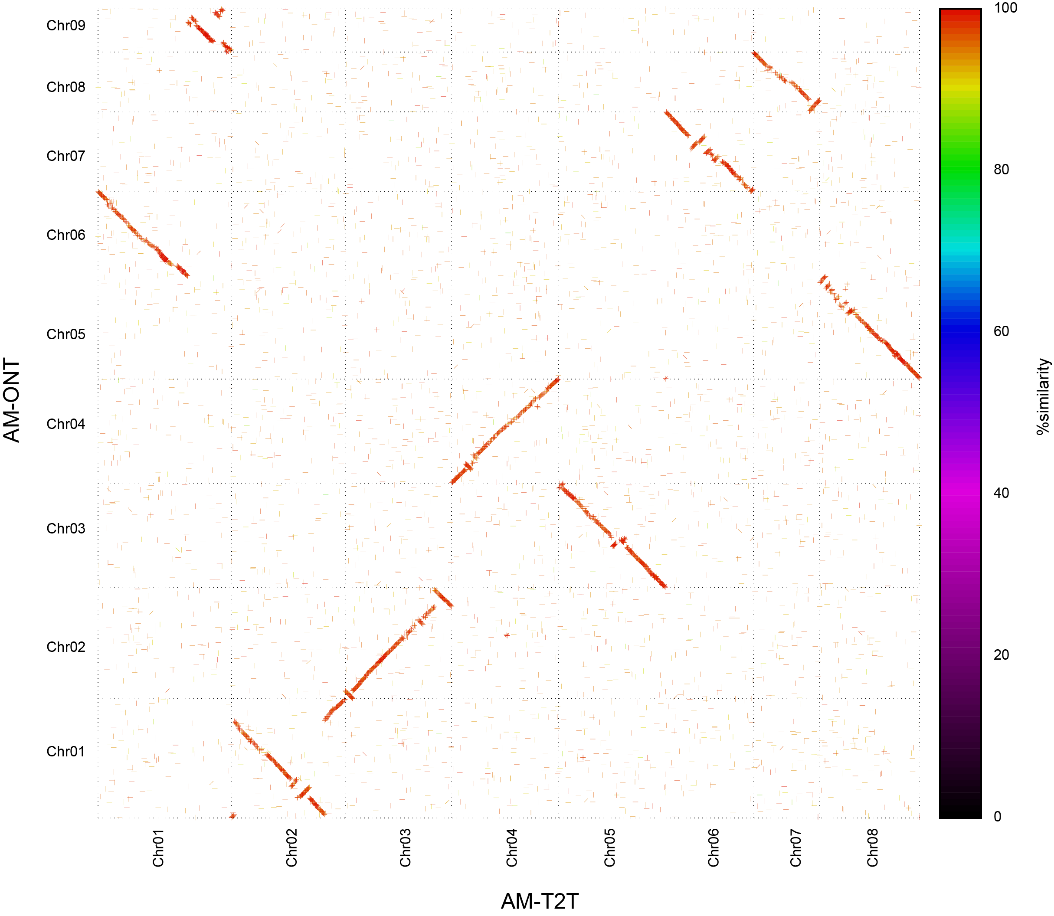
**

**Supplementary Fig. 11 Comparison of sequence synteny between AM-T2T and AM-ONT.**
