# Supplementary material for: The complete genome assembly of Astragalus membranaceus: enabling more accurate genetic research
Source: Gigascience. 2025 Oct 1;14:giaf117. doi: 10.1093/gigascience/giaf117 (PMC12486382; doi:10.1093/gigascience/giaf117)
Supplement: giaf117_GIGA-D-25-00123_Original_Submission [file giaf117_giga-d-25-00123_original_submission.pdf]

GigaScience

The complete genome assembly of Astragalus membranaceus: enabling more accurate genetic research

--Manuscript Draft--

**Manuscript Number:**	GIGA-D-25-00123
**Full Title:**	The complete genome assembly of Astragalus membranaceus: enabling more accurate genetic research
**Article Type:**	Data Note
**Funding Information:**	Fundamental Research Program of Shanxi Province (202103021224158) — Associate Research Fellow Qin Huibin<br>the National Natural Science Foundation of China (31601457) — Associate Research Fellow Qin Huibin<br>Chinese Materia Medica, China Agriculture Research System (CARS-21) — Research Fellow Hongling Tian<br>Hengshan Astragalus Research Institute's Local Partnership Project (XDHZHQY2022-01) — Research Fellow Hongling Tian
**Abstract:**	Background: Astragalus membranaceus (Fisch.) Bunge is a globally significant medicinal plant renowned for its potent immunomodulatory and antioxidant properties. However, the existing reference genome for this species remains incomplete, characterized by fragmented assemblies and the absence of centromeric and telomeric regions, thereby limiting comprehensive exploration of the genetic mechanisms underlying its key traits.<br>Findings : We hereby present the first complete genome assembly for Astragalus membranaceus (Fisch.) Bge “AM-T2T”, generated through PacBio HiFi, ONT long reads, and Hi-C scaffolding. The assembly achieved a total size of 1.39 Gb with a N50 of 180.45 Mb. It features annotations of 64.22% repetitive sequences, 16 telomeres, 8 centromeres on 8 chromosomes, and 32,600 high-confident genes. Notably, 158.58 Mb of previously unassembled regions (PURs) were resolved, harboring 46 TFs. The assembly benefits mapping analysis for RNA-seq, outperforming two previously published genomes (AM-ONT, AM-CLR) as reference genomes. Additionally, 2,267 unique genes and 20,652 conserved genes were identified within the AM-T2T genome.<br>Conclusions : This complete genome assembly of A. membranaceus fills critical gaps in the understanding of this species and its adaptations. It represents a significant advancement in the genomic characterization of A. membranaceus, providing a robust resource that will bolster genetic research, breeding programs, and medicinal applications.
**Corresponding Author:**	Qin Huibin, Ph.D.<br>Shanxi Agricultural University<br>Taiyuan, CHINA
**Corresponding Author Secondary Information:**	
**Corresponding Author's Institution:**	Shanxi Agricultural University
**Corresponding Author's Secondary Institution:**	
**First Author:**	Qin Huibin, Ph.D.
**First Author Secondary Information:**	
**Order of Authors:**	Qin Huibin, Ph.D.<br>Huibin Qin<br>Aohui Li<br>Shuyu Zhong<br>Huazhi Wang

Powered by Editorial Manager® and ProduXion Manager® from Aries Systems Corporation

	Hongling Tian
**Order of Authors Secondary Information:**	
**Additional Information:**	
**Question**	**Response**
Are you submitting this manuscript to a special series or article collection?	No
**Experimental design and statistics**<br><br>Full details of the experimental design and statistical methods used should be given in the Methods section, as detailed in our Minimum Standards Reporting Checklist. Information essential to interpreting the data presented should be made available in the figure legends.<br><br>Have you included all the information requested in your manuscript?	Yes
**Resources**<br><br>A description of all resources used, including antibodies, cell lines, animals and software tools, with enough information to allow them to be uniquely identified, should be included in the Methods section. Authors are strongly encouraged to cite Research Resource Identifiers (RRIDs) for antibodies, model organisms and tools, where possible.<br><br>Have you included the information requested as detailed in our Minimum Standards Reporting Checklist?	Yes
**Availability of data and materials**<br><br>All datasets and code on which the conclusions of the paper rely must be either included in your submission or deposited in publicly available repositories (where available and ethically appropriate), referencing such data using a unique identifier in the references and in	Yes

the “Availability of Data and Materials” section of your manuscript.<br><br>Have you have met the above requirement as detailed in our Minimum Standards Reporting Checklist?	
GigaScience has policies and guidelines in place for the use of generative AI-writing tools such as ChatGPT. If you have used such writing tools to assist with writing the manuscript this must be declared and cited in the text. Authors should not list AI-writing tools and other AI-assisted technologies as an author or co-author and should acknowledge that they are fully responsible for text generated or refined by AI-writing tools.<p><br><br>A summary of use (particularly in the introduction or among methods) needs to be included at the end of the paper, and the outputs should also be included as a supplementary file hosted in GigaDB or other open repositories. Please read our guidelines for more information. <p><br><br>By submitting to GigaScience, you are aware of the journal's AI-writing tools policy, and if you have declared use of such tools below, you have acknowledged this where appropriate in your manuscript and have made a summary of use and outputs available. <p><br><b>AI-assisted writing tools have been used in the preparation of this manuscript?	No

Powered by Editorial Manager® and ProduXion Manager® from Aries Systems Corporation

Manuscript Click here to access/download;Manuscript;Manuscript0405.docx

DATA NOTE

The complete genome assembly of *Astragalus membranaceus*: enabling more accurate genetic research

Huibin Qin [1*†], Aohui Li [2†], Shuyu Zhong [2†], Huazhi Wang [2†], Hongling Tian [3*]

1 Center for Agricultural Genetic Resources Research, Shanxi Agricultural University/ Institute of Crop Germplasm Resources, Shanxi Academy of Agricultural Sciences, Key Laboratory of Crop Gene Resources and Germplasm Enhancement on Loess Plateau, Ministry of Agriculture, Shanxi Key Laboratory of Genetic Resources and Genetic Improvement of Minor Crops, Taiyuan 030031, China

2 College of Agronomy, Shanxi Agricultural University, Taigu 030801, China

3 Shanxi Agricultural University, Shanxi Academy of Agricultural Science, The Industrial Crop Institute, Taiyuan 030031, China

*Correspondence address.

Huibin Qin, China. E-mail: qinhuibin@sxau.edu.cn

Hongling Tian, China. E-mail: thl2003@163.com.

[†] These authors contributed equally to this work.

Abstract

Background: *Astragalus membranaceus* (Fisch.) Bunge is a globally significant medicinal plant renowned for its potent immunomodulatory and antioxidant properties. However, the existing reference genome for this species remains incomplete, characterized by fragmented assemblies and the absence of centromeric and telomeric regions, thereby limiting comprehensive exploration of the genetic mechanisms underlying its key traits.

Findings: We hereby present the first complete genome assembly for *Astragalus membranaceus* (Fisch.) Bge "AM-T2T", generated through PacBio HiFi, ONT long reads, and Hi-C scaffolding. The assembly achieved a total size of 1.39 Gb with a N50 of 180.45 Mb. It features annotations of 64.22% repetitive sequences, 32,600 high-confident genes, 16 telomeres, 8 centromeres on 8 chromosomes, and GCI score of 36.23. Notably, 158.58 Mb of previously unassembled regions (PURs) were resolved, harboring 46 TFs. The assembly benefits mapping analysis for RNA-seq, outperforming two previously published genomes (AM-ONT, AM-CLR) as reference genomes. Additionally, 2,267 unique genes and 20,652 conserved genes were identified within the AM-T2T genome.

Conclusions: This complete genome assembly of *A. membranaceus* fills critical gaps in the understanding of this species and its adaptations. It represents a significant advancement in the genomic characterization of *A. membranaceus*, providing a robust resource that will bolster genetic research, breeding programs, and medicinal applications.

Key words: *Astragalus membranaceus*, telomere-to-telomere genome, genome annotation, previously unassembled regions

Data Description

Context

Astragalus membranaceus (Fisch.) Bunge, a prominent member of the Fabaceae family, has been historically employed in various cultural practices across Asia. Its dried roots, known as "Huangqi" or Astragali Radix, are recognized for containing diverse bioactive compounds, including flavonoids, triterpenoids, polysaccharides, and amino acids [1]. Flavonoids are involved in plant defense mechanisms against biotic and abiotic stresses and have shown potential in antioxidant and anti-inflammatory activities beneficial to human health [2-4]. Triterpenoids such as astragalosides have been reported to demonstrate pharmacological effects such as potential immunomodulatory activity and cardiovascular protective properties in preclinical studies [5, 6]. These bioactive components make *A. membranaceus* a valuable resource for the development of new drugs and functional foods. China is the largest global producer of Huangqi, with approximately half of the annual yield processed into herbal products, while the remainder is used in pharmaceutical and extract formulations.

Recent advancements in genomics have enhanced the characterization of *A. membranaceus*. Zhang *et al*. assembled the first complete mitochondrial genome of this species, revealing a multi-chromosome structure and providing insights into the evolutionary mechanisms of this medicinal plant [7]. Additionally, Li *et al*. reconstructed the full-length transcriptome of *A. membranaceus* using PacBio Iso-Seq technology, identifying numerous transcript variants involved in the biosynthesis of bioactive compounds such as astragalosides and calycosin [8]. *De novo* genome assembly is a fundamental and powerful tool in genomics research. Recently, two versions of *A. membranaceus* genome assemblies have been developed using different sequence platforms. Base

on Pacific Biosciences (PacBio) continuous long reads (CLR) reads and chromatin conformation capture (Hi-C) technology, a total of 1.43 Gb *A. membranaceus* genome was obtained, with a contig N50 value of 1.67 Mb (AM-CLR) [9]. Furthermore, 1.47 Gb chromosome-level genome assembly of *A. membranaceus* was generated using a combination of MGI-SEQ short-read, Oxford Nanopore (ONT) long-read and Hi-C technologies (AM-ONT) [10]. Besides, the genome of *Astragalus mongholicus* (AMM), another authorized plant source of Astragali Radix, has been decoded [11]. However, these assemblies remain incomplete in repetitive sequence regions, centromeres, and telomeres. This limitation hinders a comprehensive understanding of the genetic mechanisms underlying the biosynthesis of important bioactive compounds and key traits such as growth and stress tolerance.

Telomere-to-telomere (T2T) genome assemblies are critical for deciphering genome evolution and accelerating crop improvement initiatives. Currently, advances in sequencing make T2T genome assembly feasible, enabling comprehensive genome identification. PacBio high-fidelity (HiFi) sequencing generates highly accurate long-read datasets with mean read lengths of 10-25 kb and >99.9% base accuracy. The primary determinant of assembly complexity is not the size of the genome, but the repetitive sequence. ONT has addressed this challenge through ultra-long-read methods producing reads averaging ~50 kb (with lengths exceeding 100 kb), overcoming limitations posed by repetitive regions refractory to HiFi assembly. Leveraging these methodologies, several important crops, such as rice [12], maize [13], and sorghum [14], have successively released T2T-level genomes. Notably, recent reviews have systematically synthesized the methodological frameworks and key considerations for undertaking T2T genome assemblies [15, 16].

Therefore, we integrated PacBio HiFi sequencing, ONT ultralong sequencing, and Hi-C technology to assemble a complete genome assembly for *Astragalus membranaceus* (Fisch.) Bge (Fig. 1). This assembly enabled the first analysis of the telomeric and centromeric regions. Furthermore, we used our T2T genome to identify PUR regions, and found genes in these regions. Also, unique genes and conserved genes were studied. This T2T genome assembly marks a significant advancement in *A. membranaceus* genomics, providing a solid foundation for diverse downstream comparative genomic analyses and pan-genome studies.

Methods

Sample collection

The study materials (SXHQ0000254) used in this study was collected from the Zhengyao Garden of Gansu University of Traditional Chinese Medicine. The voucher specimen is currently deposited in the medicinal plant experimental field of the Fenyang Germplasm Resource Nursery of Chinese Medicinal Materials, located in Lvliang City, Shanxi Province. High-quality genomic DNA was extracted from healthy young leaves. All samples were frozen in liquid nitrogen and stored at -80°C for preservation and subsequent analysis.

Karyotype analysis

Root tips were excised from germinated seeds and pretreated in a solution containing 0.001 mol/L 8-hydroxyquinoline and 0.02% colchicine (1:1, v/v) at 4°C for 4 hours to synchronize mitotic cells at metaphase. After thorough rinsing, samples were fixed in freshly prepared Carnoy's fixative (methanol: acetic acid, 3:1, v/v) for 4-24 hours and hydrolyzed in 1 mol/L HCl at 60°C for 10

minutes. To improve cell wall digestion, enzymatic treatment with a cellulase-pectinase mixture (6%:4%, 2:1, v/v) was performed at 37°C for 5-6 hours, followed by a low-osmotic treatment in distilled water at 37°C for 30 minutes. Chromosomes were stained with carbol fuchsin, and slides were prepared using a standardized squash protocol. Chromosome images were captured using a Nikon 80i microscope equipped with a cold CCD camera, and karyotype parameters were analyzed using Zeiss Karyotype software.

Sequencing and filtering

For HiFi sequencing, SMRTbell target size libraries were constructed according to PacBio's standard protocol (Pacific Biosciences) using about the 16-kb preparation solutions with the SMRTbell Express Template Prep Kit 2.0. The sequencing was conducted in HiFi mode on the PacBio Revio platform (RRID:SCR_017990) at BGI Genomics. The PacBio HiFi reads, initially generated in BAM format, were converted to FASTQ format using the bam2fastq tool (version 1.0.0) [17]. For ONT sequencing, ONT ultra-long insert libraries were obtained using the Oxford Nanopore SQK-LSK109 kit and sequenced on the PromethION (RRID:SCR_017987) platform The ONT data underwent processing using NanoFilt version 2.8.020 (RRID:SCR_016966) [17] with a quality threshold of 7. Previous studies of AM-CLR provided RNA-seq and Hi-C reads for supplementary analysis.

Genome assembly and Hi-C scaffolding

At first, the contigs were generated using various method. Ultra-long ONT reads were processed using Next Denovo version 2.5.2 (RRID:SCR_025033) [18]. Flye version 2.9.4

(RRID:SCR_017016) [19] and Wtdbg2 version 2.5 (RRID:SCR_017225) [20] were employed to assemble the contigs with HiFi reads. The backbone contigs were generated with Hifiasm version 0.19.9 (RRID:SCR_021069) [21] using the command: " hifiasm -o AM.asm -t 50 --ul-cut 1000 --n-hap 2 --telo-m TTTAGGG --h1 $HiC_fq1 --h2 $HiC_fq2 --ul $ont $hifi". Hi-C reads (accession number: SRR27790545) were utilized to anchor chromosomes with Haphic version 1.0.6 [22]. An additional error correction step was carried out with Juicebox version 2.13.07 (RRID:SCR_021172) [23] according to the interaction signal. Contigs obtained by NextDenovo version 2.5.2 (RRID:SCR_025033) [18]. Flye version 2.9.4 [19] and Wtdbg2 version 2.5 (RRID:SCR_017225) [20] were used for filling gaps with quartet_gapfiller.py script from quarTeT version 1.1.1 [24]. As recommended, the specific parameters used were “-f 5000 -l 1000 -i 40 -m 1000000 -t 20”. In addition, we applied the LR_Gapcloser (RRID:SCR_017021) [25] program with HiFi reads to close the remained gaps in the assembled chromosomes, referring to the methods described in the telomere-to-telomere genome assembly of sorghum [14]. To enhance genome quality, Winnowmap version 2.03 (RRID:SCR_025349) [26] was used to align HiFi reads to the chromosomes, followed by filtering to exclude secondary alignments and excessive clipping with the 'falconc bam-filter-clipped' tool. Finally, Racon version 1.5.0 (RRID:SCR_017642) [27] was performed for further polishing with the filtered alignments.

The completeness of the genome assembly was assessed utilizing Benchmarking Universal Single-Copy Orthologs (BUSCO) version 5.4.3 (RRID:SCR_015008) [28] with the embryophyta_odb10 database, which included 1,614 orthologs. The quality value (QV) was evaluated by Merqury program version 1.3 (RRID:SCR_022964) [29] with 17-mer. Long reads from ONT and HiFi were aligned to the assembly with Minimap2 version 2.24-r1122

(RRID:SCR_018550) [30]. After identifying LTR structures and using complete LTR elements to calculate the LTR assembly index (LAI) value, we performed calculations to determine the genome assembly integrity, which was quantified using the LAI score [31]. In addition, the Genome Continuity Inspector (GCI) was assessed using GCI version 1.0 [32]. At last, a new reference-free tool, Clipping information for Revealing Assembly Quality (CRAQ), was employed to scan the regions of low quality in the genome assembly [33].

Genome annotations

The content of repetitive sequences in the AM-T2T was predicted using homology searching and the *ab initio* prediction method. For homology-based prediction, Repeat Masker version 4.0.7 [34] and Repeat Protein Mask version 4.0.7 were used to search against rebase. For *ab initio* prediction, LTR_FINDER version 1.07 (RRID:SCR_015247) [35] and Repeat Modeler version 1.0.8 were carried out. Tandem Repeats Finder version 4.10 (RRID:SCR_022193) [36] was used to identify the tandem repeat elements.

The gene prediction process employed a comprehensive strategy that integrated transcriptome-based, and homology-based methods. Initially, RNA-seq clean reads were assembled using Trinity version 2.8.5 (RRID:SCR_013048) [37], with the parameters '--max_memory 200G --CPU 40 --min_contig_length 200 --genome_guided_bam merged_sorted.bam --full_cleanup --min_kmer_cov 3 --min_glue 3 --bfly_opts '-V 5 --edge-thr=0.1 --stderr' --genome_guided_max_intron 10000', yielded 221,161 transcripts with a N50 size of 1,636. The assembled transcripts were then aligned to the assembly using Program to Assemble Spliced Alignment (PASA) version 2.4.1 (RRID:SCR_014656) [38], generating gene structures from valid

transcript alignments (PASA-set). Additionally, RNA-seq clean reads were mapped to the assembly via Hisat2 version 2.0.1 (RRID:SCR_015530) [39]. Subsequently, Stringtie version 1.2.2 (RRID:SCR_016323) [40] and TransDecoder version 5.7.1 (RRID:SCR_017647) were employed to assemble the transcripts and identify candidate coding regions, resulting in the creation of gene models (Stringtie-set). Homologous genomes from seven assemblies, including AM-CLR, *Astragalus membranaceus* var. mongholicus [11], *Arabidopsis thaliana* Col-PEK [41], *Glycine max* (ZH13-T2T) [42], *Trifolium pratense* (ensembl release-59), *Phaseolus vulgaris* (ensembl release-59), and *Medicago truncatula* (ensembl release-59) were downloaded and used as queries to search against the assembly using GeMoMa version 1.9 (RRID:SCR_017646) [43]. These homology predictions were referred to as "Homology-set". The gene models from these three sources were subsequently merged using EvidenceModeler version 2.1.0 (RRID:SCR_014659) [44], with different weight parameters assigned to evidence from different sources (10 for Homology-set, 5 for Stringtie-set, and 5 for PASA-set). Finally, the generated gene models underwent further refinement with PASA version 2.4.1 [38] to obtain untranslated regions and alternative splicing variation information. The integrated gene set was translated into amino-acid sequences and annotated using the method described the telomere-to-telomere genome assembly of sorghum [14].

Following the method used in the blister beetles transcriptome, gene expression analysis was carried out using fragments per kilobase of transcript per million mapped reads (FPKM) method [45]. Transcription factor (TF) prediction was performed using the method described in eggplant genome study [46]. Transfer RNAs (tRNAs) and ribosomal RNAs (rRNAs) were predicted using tRNAscan-SE version 1.3.1 (RRID:SCR_008637) [47] and BLASTN (RRID:SCR_001598; *E*-

value ≤ 1e−05) against the rRNA sequences of both *Arabidopsis thaliana* and *Oryza sativa*, respectively. Both microRNAs (miRNAs) and small nuclear RNAs (snRNAs) were identified by searching against the Rfam database (RRID:SCR_010835, release 12.0) using Infernal version 1.1.1 (RRID:SCR_010835).

Telomere and centromere identification

Following a method similar to that described in the study of the complete broomcorn millet assembly [48], we used quarTeT (RRID:SCR_025258) version 1.1.5 TeloExplorer to identify telomeres and CentroMiner to identify centromeres [24]. Given the complex structure of centromeres, we further employed Centromics (https://github.com/zhangrengang/Centromics) to identify centromeres by detecting high-copy tandem repeats from HiFi sequencing data.

Gene families analysis

Protein sets of nine species (*A. membranaceus* var. *mongholicus*, *A. thaliana, Cicer arietinum, Cajanus cajan, G. max, M. truncatula, Lupinus angustifolius, Vigna angularis,* and AM-T2T) were employed in the orthology identification with *A. thaliana* as the out-group. The OrthoMCL version 2.0.9 (RRID:SCR_007839) was applied to determine and cluster gene families among these nine plant species. A total of 1,153 single-copy orthologs among these species were multiply aligned with Muscle version 3.8.1551 (RRID:SCR_011812), then concatenated and used for constructing a phylogenomic tree using IQtree2 version 2.3.6 with parameters of “-B 1000 -m MFP”. Gene Ontology (GO) enrichment was conducted using ClusterProfiler version 4.2.2 (RRID:SCR_016884) to explore the functional characteristics of the unique gene families in the AM-T2T genome.

Results

Complete genome assembly and completeness evaluation

The somatic chromosome complement of *A. membranaceus* displayed a diploid constitution of 2n = 2x = 16, consistent with previous cytogenetic investigations on this species and its variants [49, 50] (Fig. 1B).

We newly sequenced the genome of *A. membranaceus*, generating 92.74 Gb (~66.72 × coverage) of PacBio HiFi reads, and 38.53 Gb (~ 27.72 × coverage) of ONT reads (Supplementary Table S1). Using different data, contigs were constructed with four assembly tools, including Hifiasm, Wtdgb2, Flye, and NextDenovo. Based on the mixed data, Hifiasm produced the most contiguous assemblies, with a contig N50 of approximately 120.48 Mb, and had the smallest number of sequences (Supplementary Table S2). Thus, this assembly served as the backbone for scaffolding contigs while the other contigs were used for downstream gap closing analysis. As a result, a total of 1.41 Gb of Hifiasm assembly sequences were anchored to eight pseudochromosomes, with eight gaps distributed across six of the pseudochromosomes (Supplementary Table S3). After filling all remaining gaps and further polish, a gap-free reference genome named AM-T2T was generated, containing a total length of 1.39 Gb (Table 1).

To assess the accuracy and completeness of the AM-T2T assembly, various methods were employed. First, PacBio HiFi reads were mapped onto the genome, resulting in 100% mapping rate and 99.97% genome coverage (Table 1). In particular, the CRAQ analysis revealed that only 0.02% of the genome was classified as low confidence (Supplementary Table S4). Within these low-confidence regions, the proportion of repetitive sequences was 72.60%, significantly higher than the 64.22% of repetitive sequences in the entire genome. Second, the BUSCO analysis

demonstrated that the completeness of the AM-T2T genome reached 99.63%, surpassing the levels observed in the AM-ONT and AM-CLR assemblies (Table 1). Notably, the RNA-seq mapping analysis revealed that AM-T2T was more suitable for analyzing RNA-seq data, with higher mapping rates (average mapping rate: 88.58%) compared to AM-CLR (average mapping rate: 86.15%) and AM-ONT (average mapping rate: 81.17%) (Supplementary Table S5). Third, the LAI value of the AM-T2T assembly was 22.67, meeting the gold standard for genome assemblies [31]. Fourth, the calculated QV of the genome was 57.51, indicating a base call accuracy higher than 99.999% [29]. Fifth, the Hi-C heatmap demonstrated a high degree of consistency across all pseudochromosomes, confirming the precision in sequencing, ordering, and orientation of contigs (Fig. 2A). The GCI score for the AM-T2T genome was 36.23 (Table 1), significantly surpassing the GCI score of the chicken complete genome (29.37) [32]. Last, using the seven-base telomeric repeat as a sequence query, we identified all the 16 telomeres for the genome (Fig. 2B). Considering all these factors, these comprehensive validation results collectively affirm the exceptional quality and reliability of the AM-T2T assembly.

Genome annotation

A combination of *de novo* and homology-based approaches was employed to annotate repetitive sequences in the AM-T2T genome. Using these methods, we successfully identified a total of 890.27 Mb of repetitive sequences, constituting 64.22% of the entire genome (Supplementary Table S6). This repetitive fraction was slightly smaller than those observed in the AM-CLR and AM-ONT (Table 1). The predominant repeat classes in AM-T2T were LTR retrotransposons and DNA transposons, which constituted 55.60% and 5.54% of the genome, respectively

(Supplementary Table S7). Additionally, 16,417 noncoding RNAs were identified, including 109 miRNAs, 1,544 tRNAs, 4,690 snRNAs, and 10,074 rRNAs (Supplementary Table S8).

A total of 32,600 coding genes were predicted in this genome, with an average coding sequence (CDS) length of 1,169.09 bp (Fig. 3; Table 1; Supplementary Table S9). BUSCO analysis indicated that 99.07% of core conserved plant orthologs were fully detected in the AM-T2T genome (Supplementary Table S10). This completeness level surpassed the metrics observed in the AM-CLR (96.59%) and AM-ONT (97.27%) assemblies. The length distribution of messenger RNA, CDS, exons, and introns among related species supported the reliability of the annotation results (Supplementary Fig. S1). The functional analysis revealed that 98.34% of the coding genes could be annotated through publicly protein datasets, suggesting the accuracy of gene prediction (Supplementary Table S11). In addition, 24,181 (74.17%) genes showed detectable transcriptional activity (FPKM≥1) (Supplementary Fig. S2). Moreover, 2,187 TFs were predicted across 58 types, surpassing the count that in the AM-CLR genome (2,048) [51] (Supplementary Fig. S3). These findings affirmed the completeness and accuracy of gene prediction in the AM-T2T assembly.

The characteristics of centromeric regions

The centromeric region of the genome presents a notable assembly challenge due to its high content of repetitive sequences [52]. So far, the centromeric sequence of the *A. membranaceus* genome has not been fully characterized, and our new T2T genome allows deeper exploration of the repeats in these regions. In the AM-T2T genome, the centromeric sequences of the eight pseudochromosomes were assembled, with an average length of 2,949,226 bp (Table 2). The longest centromeric region, located on pseudochromosome 1, spanned 6,931,092 bp, while the shortest, on pseudochromosome

6, measured 351,503 bp. On average, centromere sequences comprised 88.63% repeat sequences, with the primary transposable elements (TEs) being DNA transposons and LTRs. Notably, the distribution of TEs within the centromeric regions varied across chromosomes, with LTRs predominating in Chromosome 1, and DNA transposons prevalent in Chromosomes 3, 4, 5, and 6. In addition, the average content of tandem repeats is 54.49%, which is much higher than that in the whole genome (8.43%). These results are consistent with previous reports highlighting centromeric enrichment of retrotransposons and tandem repeats [53]. Within the chromosome centromeres of AM-T2T, a total of 169 genes were identified. Function enrichment analysis showed that these genes were significantly enrichment in multiple GO terms, such as “nucleic acid binding”, “RNA-DNA hybrid ribonuclease activity”, and “chitinase activity”, suggesting their potential functions in the segregation of homologous chromosomes (Supplementary Fig. S4). Among these centromeric genes, 99 (58.58%) were expressed with an FPKM value greater than 1, slightly lower than the expression ratio observed among all annotated genes (74.17%).

Unique genes and conserved genes

To explore the unique genes and conserved genes between AM-T2T and other embryophyta, we selected nine species for the construction of gene families and the phylogenomic tree. The statistical analysis of gene family identification results showed that a total of 28,023 gene families were identified, including 9,991 that were common to the nine species. There were 169 gene families unique for AM-T2T genome, containing 2,267 genes (Figure 4A; Supplementary Table S12). Among these unique genes, 2,045 (90.21%) genes were supported by functional annotation and 1,128 (49.76%) genes had a FPKM value above 1.0 in at least one sample (Supplementary Table

S13). The expression genes were significantly enriched in 34 GO terms, such as “nucleic acid binding”, “DNA binding”, “RNA-DNA hybrid ribonuclease activity”, “zinc ion binding”, “translation”, among others (Supplementary Fig. S5). Notably, among the expressed genes, 47 TFs were identified, implying a potential role in transcriptional regulation influencing the physiological traits of *A. membranaceus*. A set of 1,153 single-copy genes were utilized for constructing the phylogenomic tree, which indicated that *A. membranaceus*, *A.mongholicus*, *C.arietinum*, and *M.truncatula* clustered together in a major branch (Supplementary Fig. S6). Out of the 19,271 gene families in the AM-T2T assembly, 14,674 (76.14%) were found within this major branch, encompassing 20,652 genes (Figure 4B). These genes exhibited enrichment in 44 GO terms, with "ATP binding" (GO:0005524) being the most significant (Supplementary Fig. S7).

Whole genome comparative analysis

The comparative analysis of the whole genome revealed significant structural differences between the AM-T2T genome and the AM-CLR genome on chromosome 7. Specifically, chromosome 7 of the AM-T2T genome aligns with chromosomes 7 and 8 of the AM-CLR genome (Figure 5A). Detailed comparison identified that the fusion region of chromosomes 7 and 8 in the AM-CLR genome corresponds precisely to the 55.90 Mb to 56.95 Mb interval of chromosome 7 in the AM-T2T genome. Further alignment analysis between the AM-T2T and AM-ONT genomes suggested that chromosome 1 of the AM-T2T genome corresponds to chromosomes 6 and 9 of the AM-ONT genome (Figure 5B). The fusion region of chromosomes 6 and 9 in the AM-ONT genome corresponds to the 149.27–151.44 Mb on chromosome 1 of the AM-T2T genome. To validate the accuracy of the above-mentioned fusion region assembly, a genome wide comparative analysis of

the AM-T2T and AMM genomes was conducted. The results confirmed that both chromosome 1 and chromosome 7 of the AM-T2T genome align with single chromosomes in the AMM genome (Figure 5C). Additionally, by aligning HiFi reads and ONT reads to the AM-T2T genome, we observed that both HiFi reads and ONT reads provided comprehensive coverage of the fusion region on chromosome 1 (Supplementary Fig. S8). In contrast, while the coverage of HiFi reads was suboptimal for the fusion region on chromosome 7, ONT reads spanned this region, thereby supporting the assembly of this segment (Supplementary Fig. S9). This finding underscores the critical role of ONT reads in enhancing assembly quality.

A total of 158.58 Mb of previously unassembled regions (PURs) were identified through comparison of the AM-T2T genome with the two publicly available *A. membranaceus* genomes (AM-ONT and AM-CLR) (Supplementary Table S14). Most of these PURs were located within repetitive regions, comprising 123.25 Mb (77.72%) of repetitive sequences. Furthermore, 898 genes were annotated within these PURs, including 46 transcription factors (Supplementary Table S15). GO enrichment analyses revealed that these PUR-associated were involved in a variety of essential biological processes, such as “zinc ion binding”, “nucleic acid binding”, “ADP binding”, and more (Fig. 5D).

Conclusions

This study presents the first high-quality telomere-to-telomere genome assembly of *A. membranaceus*, generated using PacBio HiFi reads, ONT sequencing, and Hi-C technologies. The assembled genome spans 1.39 Gb, encompassing 16 telomeres and 8 centromeres distributed across 8 chromosomes. The high quality of the assembly was verified by a 100% mapping rate of PacBio

HiFi reads, 99.63% BUSCO completeness, higher RNA-seq mapping rates, a LAI of 22.67, and a QV of 57.51. Genome annotation revealed 64.22% repetitive sequences, 32,600 protein-coding genes, and 169 centromeric genes. Additionally, 158.58 Mb of PURs, 2,267 unique genes, and 20,652 conserved genes were identified. Genome-wide comparative analysis uncovered significant structural variations in chromosome 1 and chromosome 7 relative to previously published genome assemblies of this species. This study demonstrates the utility of advanced sequencing technologies in resolving complex genomic regions and provides a more accurate foundation for genetic research of *A. membranaceus*.

Abbreviations

BLAST: Basic Local Alignment Search Tool; BUSCO: Benchmarking Universal Single-Copy Orthologs; CLR: continuous long reads; CRAQ: Clipping information for Revealing Assembly Quality; FPKM: fragments per kilobase of transcript per million mapped reads; Gb: gigabase pairs; GCI: Genome Continuity Inspector; GO: Gene Ontology; Hi-C: High-Throughput Chromosome Conformation Capture; HiFi: High-Fidelity; IGV: Integrative Genomics Viewer; LTR: long terminal repeat; Mb: megabase pairs; miRNAs: microRNAs; NCBI: National Center for Biotechnology Information; ncRNA: non-coding RNA; NR: NCBI’s nonredundant database; PASA: Program to Assemble Spliced Alignments; PUR: previously unassembled region; QV: quality value; RNA-seq: RNA sequencing; rRNAs: ribosomal RNAs ; snRNAs: small nuclear RNAs; T2T: telomere-to-telomere; tRNAs: Transfer RNAs

Additional Files

Supplementary Table 1. Summary of newly generated whole genome sequencing data used in this study.

Supplementary Table 2. The statistics of the contig assembly.

Supplementary Table 3. The statistics of the anchored chromosome length.

Supplementary Table 4. The low confidence region within the AM-T2T assembly.

Supplementary Table 5. Summary of the RNA-seq reads genome mapping rate.

Supplementary Table 6. General statistics of repeats in AM-T2T assembly.

Supplementary Table 7. The summary of interspersed repeat contents in AM-T2T assembly.

Supplementary Table 8. Annotation of ncRNA in the AM-T2T assembly.

Supplementary Table 9. The length statistics of genes in AM-T2T assembly.

Supplementary Table 10. BUSCOs analysis of AM-T2T gene set completeness.

Supplementary Table 11. Number of functional annotations for predicted genes in AM-T2T assembly.

Supplementary Table 12. Gene families in AM-T2T and other species.

Supplementary Table 13. The functional annotation of AM-T2T unique genes.

Supplementary Table 14. The identification of PUR region in AM-T2T assembly.

Supplementary Table 15. The gene list in PUR region of AM-T2T assembly.

Author Contributions

Hongling Tian designed this study; Huazhi Wang collected the samples and performed the experiments; Aohui Li and Shuyu Zhong performed the data analysis; Huibin Qin wrote the first

draft of the manuscript. All other authors proofread and revised the manuscript. All authors read and approved the final manuscript.

Funding

This research was funded by the Fundamental Research Program of Shanxi Province (No. 202103021224158), the National Natural Science Foundation of China (No. 31601457), Chinese Materia Medica, China Agriculture Research System (CARS-21), and the Hengshan Astragalus Research Institute's Local Partnership Project (XDHZHQY2022-01).

Data Availability

The raw sequencing data that support the findings of this study have been deposited into the CNGB Sequence Archive of China National GeneBank DataBase with accession number CNP0006722.

Competing Interests

The authors declare that they have no competing interests.

Acknowledgements

We thank every project that provides funding and material support for the study. We also thank each author for their ideas and skills in study design, experimentation, data collection, data analysis, and manuscript writing.

References

1. Fu, J., et al., Review of the botanical characteristics, phytochemistry, and pharmacology of Astragalus membranaceus (Huangqi). Phytotherapy research : PTR, 2014. **28**(9): p. 1275-1283.

2. Auyeung, K.K., Q.-B. Han, and J.K. Ko, Astragalus membranaceus: A Review of its Protection Against Inflammation and Gastrointestinal Cancers. The American journal of Chinese medicine, 2016. **44**(1): p. 1-22.
3. Li, C.-X., et al., Astragalus polysaccharide: a review of its immunomodulatory effect. Archives of pharmacal research, 2022. **45**(6): p. 367-389.
4. Chen, J., et al., Global transcriptome analysis profiles metabolic pathways in traditional herb Astragalus membranaceus Bge. var. mongolicus (Bge.) Hsiao. BMC genomics, 2015. **16 Suppl 7**: p. S15.
5. Kim, Y.B., et al. Accumulation of astragalosides and related gene expression in different organs of Astragalus membranaceus Bge. var mongholicus (Bge.). Molecules (Basel, Switzerland), 2014. **19**, 10922-10935 DOI: 10.3390/molecules190810922.
6. Kim, Y.B., et al., Accumulation of flavonoids and related gene expressions in different organs of Astragalus membranaceus Bge. Applied biochemistry and biotechnology, 2014. **173**(8): p. 2076-2085.
7. Zhang, K., et al., Assembly and comparative analysis of the first complete mitochondrial genome of Astragalus membranaceus (Fisch.) Bunge: an invaluable traditional Chinese medicine. BMC Plant Biology, 2024. **24**(1): p. 1055.
8. Li, J., et al., Long read reference genome-free reconstruction of a full-length transcriptome from Astragalus membranaceus reveals transcript variants involved in bioactive compound biosynthesis. Cell Discovery, 2017. **3**(1): p. 17031.
9. Fan, H., et al., Chromosome-scale genome assembly of Astragalus membranaceus using PacBio and Hi-C technologies. Scientific Data, 2024. **11**(1): p. 1071.
10. Xu, B., et al., Total biosynthesis of the medicinal triterpenoid saponin astragalosides. Nature Plants, 2024.
11. Chen, Y., et al., A reference-grade genome assembly for Astragalus mongholicus and insights into the biosynthesis and high accumulation of triterpenoids and flavonoids in its roots. Plant Communications, 2022. **4**.
12. Shang, L., et al., A complete assembly of the rice Nipponbare reference genome. Molecular plant, 2023. **16**(8): p. 1232-1236.
13. Chen, J., et al., A complete telomere-to-telomere assembly of the maize genome. Nature genetics, 2023. **55**(7): p. 1221-1231.
14. Li, M., et al. Telomere-to-telomere genome assembly of sorghum. Scientific data, 2024. **11**, 835 DOI: 10.1038/s41597-024-03664-8.
15. Li, H. and R. Durbin, Genome assembly in the telomere-to-telomere era. Nature Reviews Genetics, 2024. **25**(9): p. 658-670.
16. Garg, V., et al., Unlocking plant genetics with telomere-to-telomere genome assemblies. Nature Genetics, 2024.
17. De Coster, W., et al., NanoPack: visualizing and processing long-read sequencing data. Bioinformatics, 2018. **34**: p. 2666 - 2669.
18. Hu, J., et al., NextDenovo: an efficient error correction and accurate assembly tool for noisy long reads. Genome Biology, 2024. **25**(1): p. 107.
19. Kolmogorov, M., et al., Assembly of long, error-prone reads using repeat graphs. Nature Biotechnology, 2019. **37**(5): p. 540-546.
20. Ruan, J. and H. Li, Fast and accurate long-read assembly with wtdbg2. Nature Methods, 2020. **17**: p. 1-4.
21. Cheng, H., et al., Haplotype-resolved de novo assembly using phased assembly graphs with hifiasm. Nature Methods, 2021. **18**(2): p. 170-175.
22. Zeng, X., et al., Chromosome-level scaffolding of haplotype-resolved assemblies using Hi-C data without reference genomes. Nature plants, 2024. **10**(8): p. 1184-1200.
23. Durand, N.C., et al., Juicebox Provides a Visualization System for Hi-C Contact Maps with Unlimited Zoom.

Cell systems, 2016. **3**(1): p. 99-101.
24. Lin, Y., et al., quarTeT: a telomere-to-telomere toolkit for gap-free genome assembly and centromeric repeat identification. Horticulture Research, 2023.
25. Xu, G.-C., et al., LR_Gapcloser: a tiling path-based gap closer that uses long reads to complete genome assembly. GigaScience, 2018. **8**.
26. Jain, C., et al., Long-read mapping to repetitive reference sequences using Winnowmap2. Nature methods, 2022. **19**(6): p. 705-710.
27. Vaser, R., et al., Fast and accurate de novo genome assembly from long uncorrected reads. Genome research, 2017. **27 5**: p. 737-746.
28. Seppey, M., M. Manni, and E.M. Zdobnov, BUSCO: Assessing Genome Assembly and Annotation Completeness. Methods in molecular biology, 2019. **1962**: p. 227-245.
29. Rhie, A., et al., Merqury: reference-free quality, completeness, and phasing assessment for genome assemblies. Genome Biology, 2020. **21**.
30. Li, H., Minimap2: pairwise alignment for nucleotide sequences. Bioinformatics, 2018. **34**(18): p. 3094-3100.
31. Ou, S., J. Chen, and N. Jiang, Assessing genome assembly quality using the LTR Assembly Index (LAI). Nucleic acids research, 2018. **46**(21): p. e126.
32. Chen, Q., et al., GCI: a continuity inspector for complete genome assembly. Bioinformatics, 2024. **40**(11).
33. Li, K., et al., Identification of errors in draft genome assemblies at single-nucleotide resolution for quality assessment and improvement. Nature Communications, 2023. **14**(1): p. 6556.
34. Bergman, C.M. and H. Quesneville, Discovering and detecting transposable elements in genome sequences. Briefings in Bioinformatics, 2007. **8**(6): p. 382-392.
35. Xu, Z. and H. Wang, LTR-FINDER: An efficient tool for the prediction of full-length LTR retrotransposons. Nucleic acids research, 2007. **35**: p. W265-8.
36. Benson, G., Tandem repeats finder: a program to analyze DNA sequences. Nucleic acids research, 1999. **27 2**: p. 573-80.
37. Grabherr, M.G., et al., Full-length transcriptome assembly from RNA-Seq data without a reference genome. Nature biotechnology, 2011. **29 7**: p. 644-52.
38. Haas, B., Improving the Arabidopsis genome annotation using maximal transcript alignment assemblies. Nucleic Acids Research, 2003. **31**: p. 5654-5666.
39. Kim, D., B. Langmead, and S.L. Salzberg, HISAT: a fast spliced aligner with low memory requirements. Nature Methods, 2015. **12**(4): p. 357-360.
40. Kovaka, S., et al., Transcriptome assembly from long-read RNA-seq alignments with StringTie2. Genome Biology, 2019. **20**(1): p. 278.
41. Hou, X., et al., A near-complete assembly of an Arabidopsis thaliana genome. Molecular plant, 2022. **15**(8): p. 1247-1250.
42. Zhang, C., et al., The T2T genome assembly of soybean cultivar ZH13 and its epigenetic landscapes. Molecular plant, 2023. **16**(11): p. 1715-1718.
43. Jens, et al., GeMoMa: Homology-Based Gene Prediction Utilizing Intron Position Conservation and RNA-seq Data. Methods in Molecular Biology, 2019.
44. Haas, B.J., et al., Automated eukaryotic gene structure annotation using EVidenceModeler and the Program to Assemble Spliced Alignments. Genome Biology, 2008. **9**(1): p. R7.
45. Wu, Y.-M., et al. Investigation of sex expression profiles and the cantharidin biosynthesis genes in two blister beetles. PloS one, 2023. **18**, e0290245.
46. Li, D., et al., A high-quality genome assembly of the eggplant provides insights into the molecular basis of disease resistance and chlorogenic acid synthesis. Molecular ecology resources, 2021. **21**(4): p. 1274-1286.

47. Lowe, T.M. and S.R. Eddy, tRNAscan-SE: a program for improved detection of transfer RNA genes in genomic sequence. Nucleic acids research, 1997. **25**(5): p. 955-964.
48. Wang, H., et al. A complete reference genome of broomcorn millet. Scientific data, 2024. **11**, 657.
49. quan, W.S., Karyotype Analysis of *Astragalus membranaceus.* Hubei Agricultural Sciences, 2006.
50. Hong, K., karyotype diversity of six *Astragalus* species. Guihaia, 2012. **32(5)**: p. 579–582.
51. Fan, H., et al. Chromosome-scale genome assembly of *Astragalus membranaceus* using PacBio and Hi-C technologies. Scientific data, 2024. **11**, 1071.
52. Deng, Y., et al., A telomere-to-telomere gap-free reference genome of watermelon and its mutation library provide important resources for gene discovery and breeding. Molecular plant, 2022. **15**.
53. Liu, Y., et al., Genome-wide mapping reveals R-loops associated with centromeric repeats in maize. Genome research, 2021. **31**(8): p. 1409-1418.

Figures and Legends

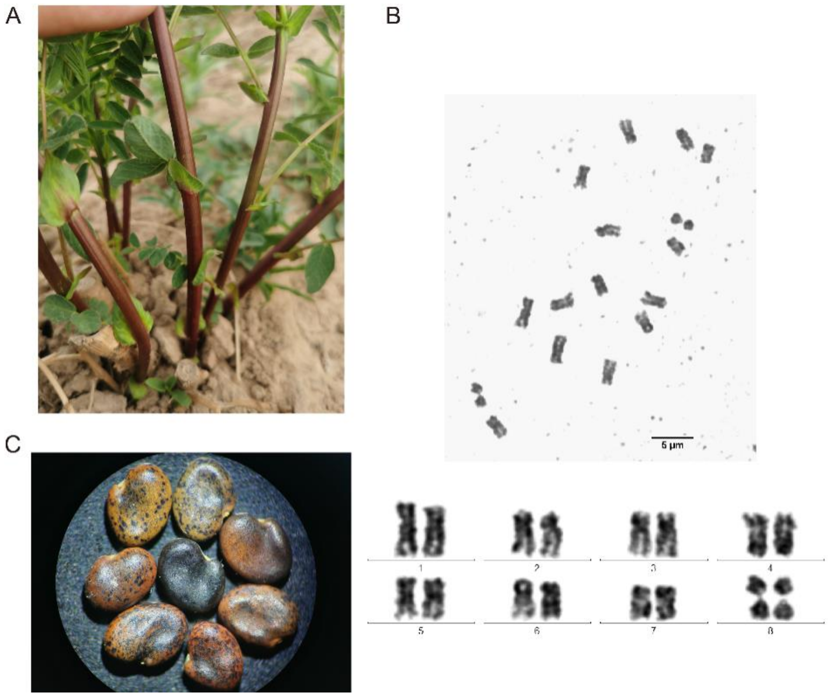

Fig. 1 | The morphology and karyotype of a *Astragalus membranaceus* (Fisch.) Bunge. A, The stems of *A. membranaceus*. **B,** The karyotype of *A. membranaceus* via karyotype analysis. **C,** The seeds of *A. membranaceus*.

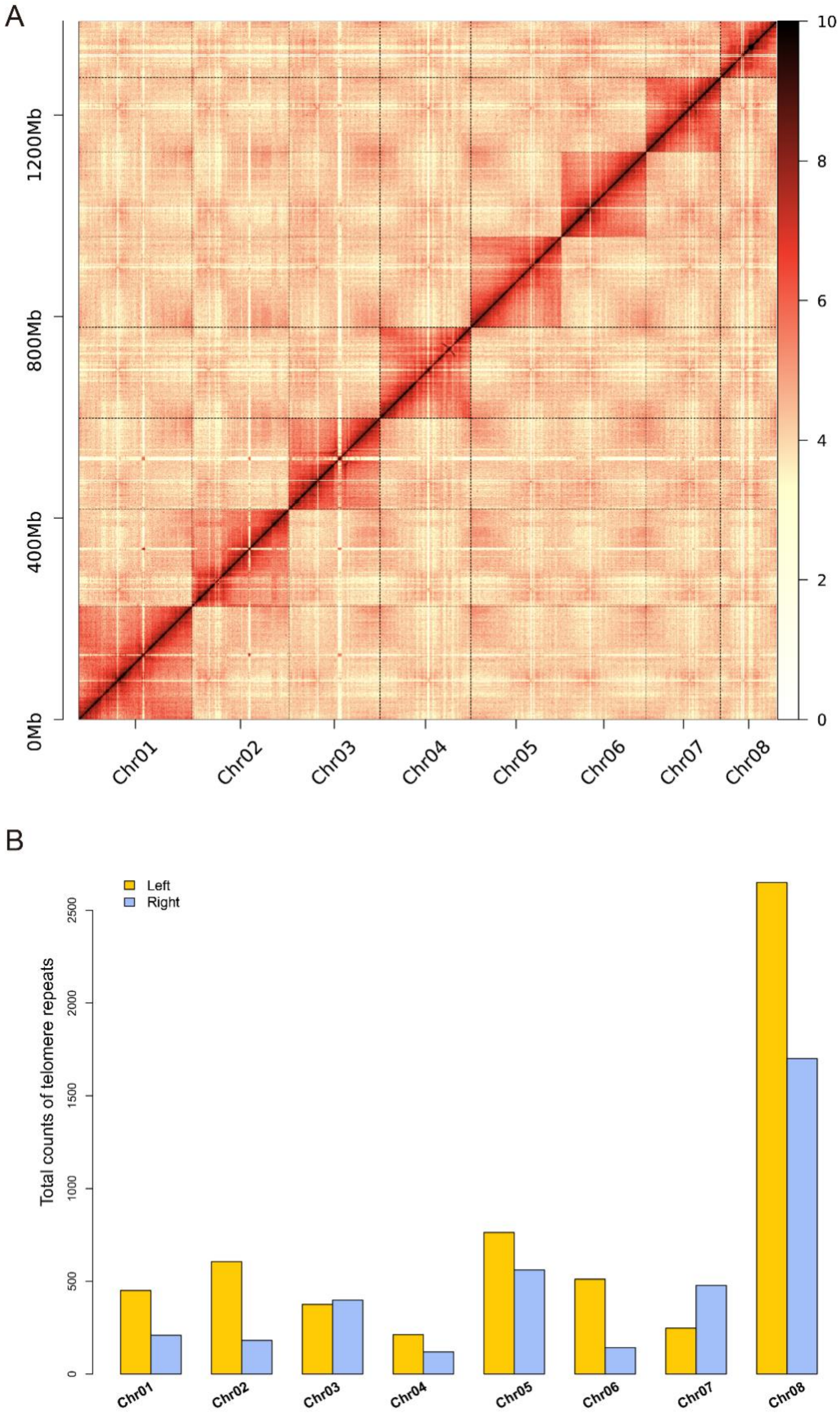

Fig. 2 | Genomic characteristics of AM-T2T assembly. A, Intensity signal heat map of the Hi-C chromosome interaction. The colour block illuminates the intensity of interaction from yellow (low) to red (high). **B**, The statistics of telomere repeats.

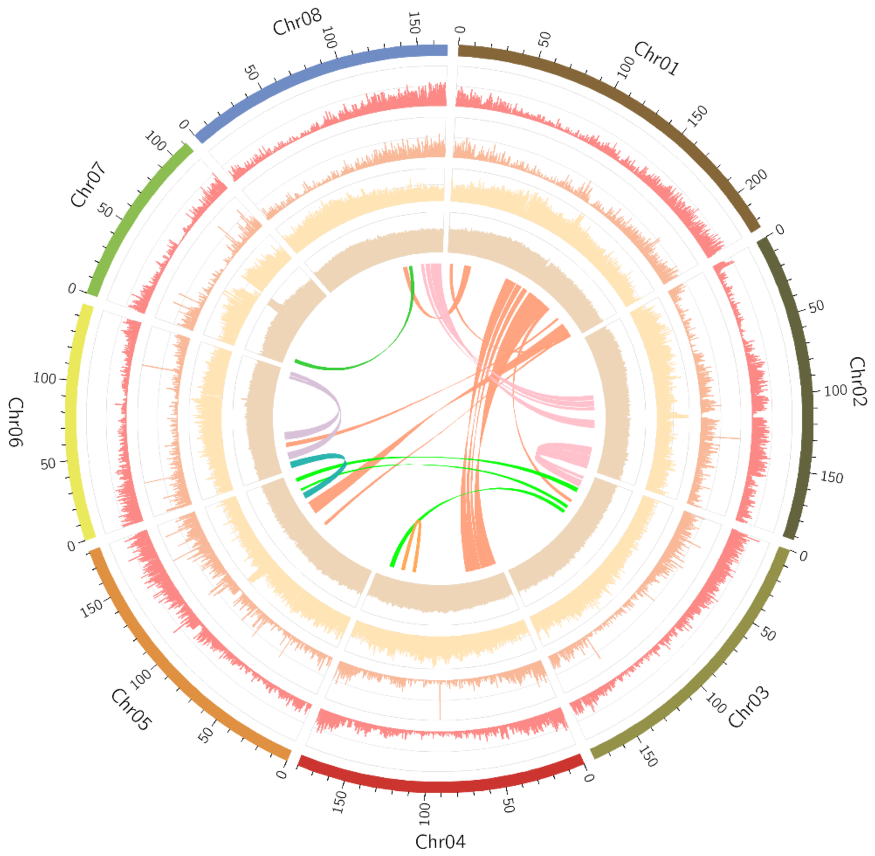

Fig. 3 | Circos plot of the assembled AM-T2T genome. Circular tracks from outside to inside indicate the pseudomolecules; gene density; gene length; repetitive density; GC content. The links in the center show syntenic region found in each chromosome.

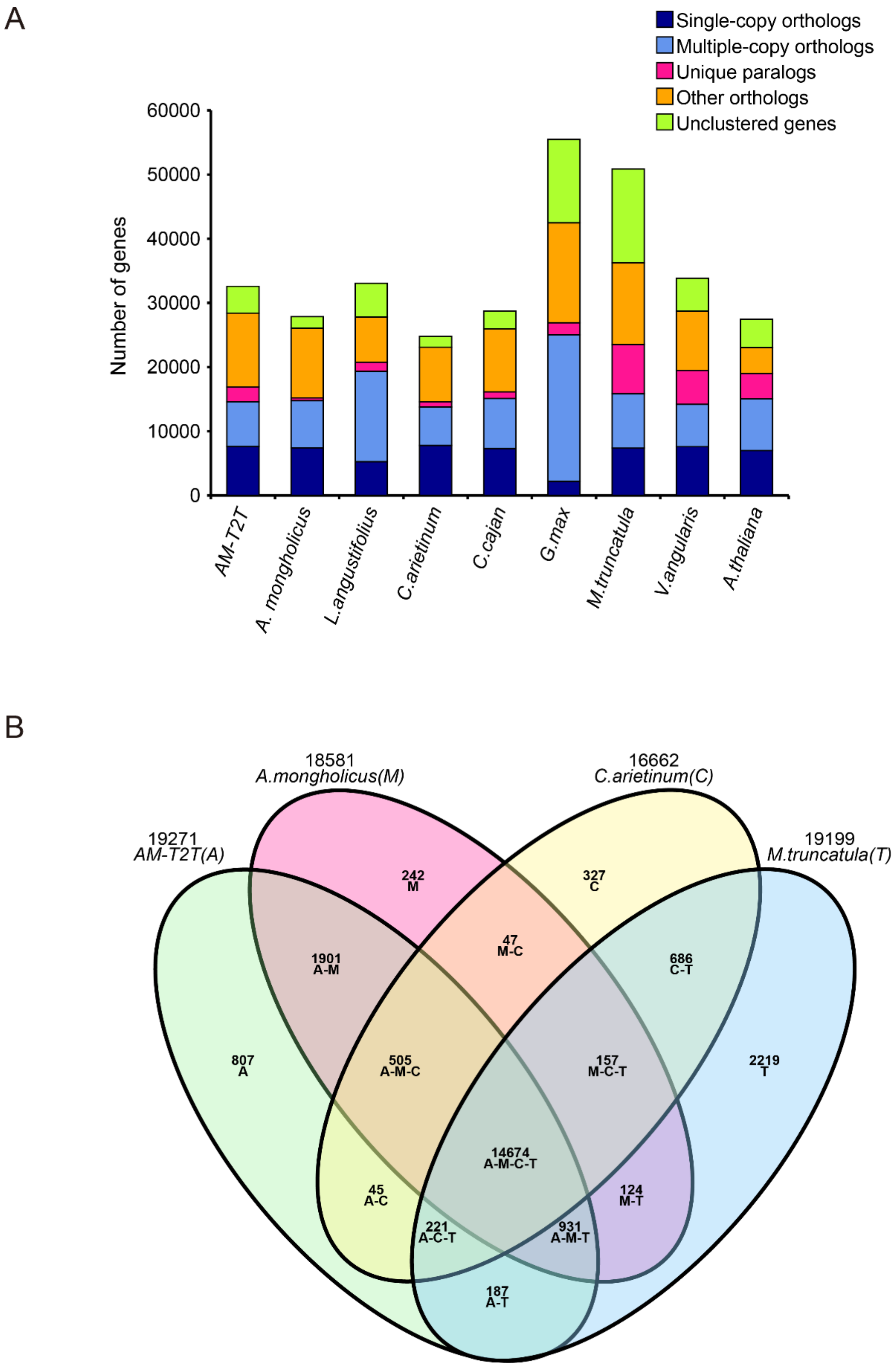

Fig. 4 | Evolution of AM-T2T genome. A, Number of orthologous genes in nine species. **B,** Venn diagram of orthologous gene families in four genomes. The numbers represent quantities of gene families.

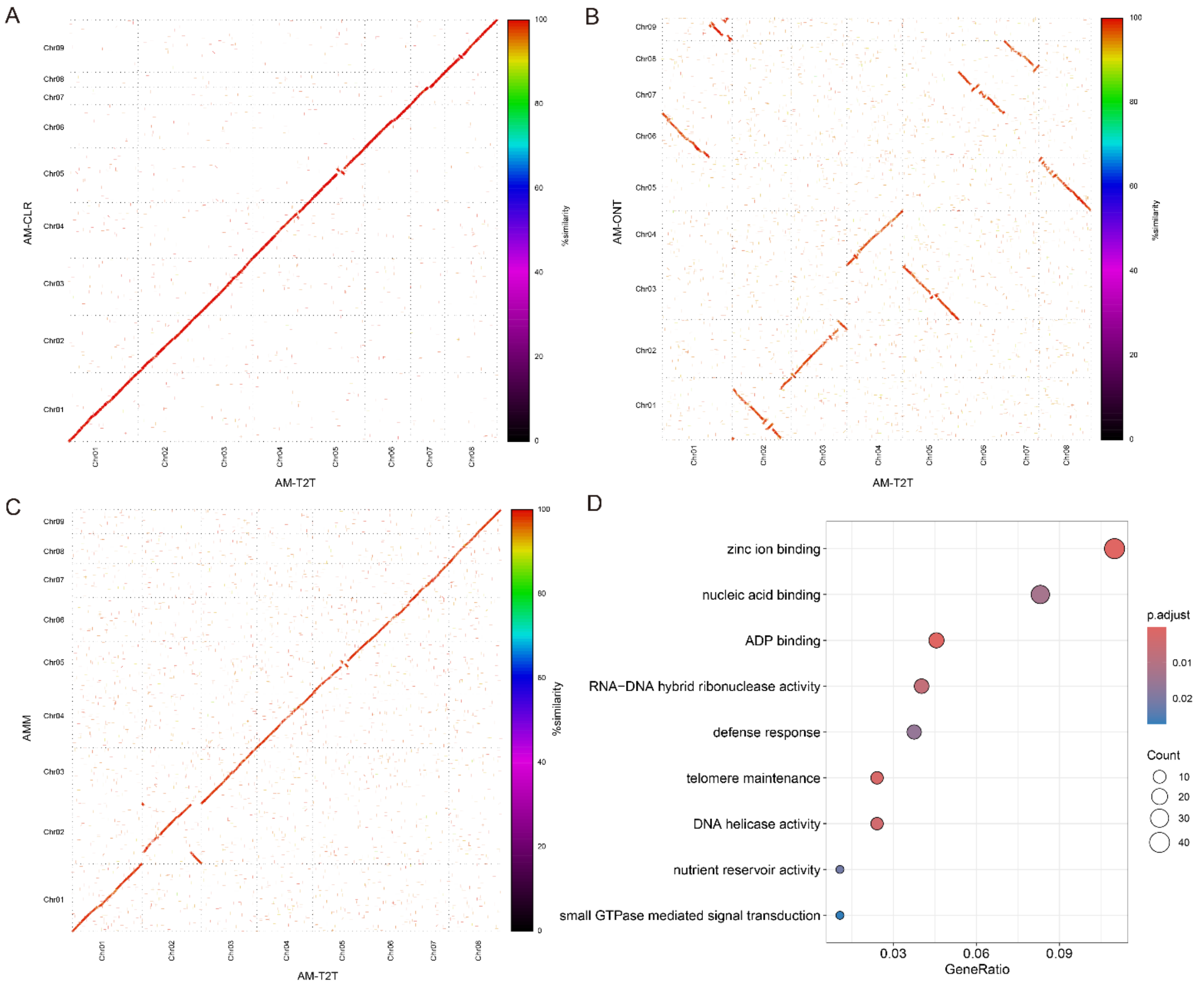

Fig. 5 | Whole genome comparative analysis of AM-T2T. A, Comparison of sequence synteny between AM-T2T and AM-CLR. **B,** Comparison of sequence synteny between AM-T2T and AM-ONT. **C,** Comparison of sequence synteny between AM-T2T and AMM. **D,** GO enrichment analysis of genes in PUR region. The bubble size indicates the gene number of a biological process GO term, with color maps the *p*.adjuct of the enrichment analysis.

Table 1. Assembly statistics of *A. membranaceus* genome assembly.

Genomic feature	AM-T2T
Number of contigs (gaps)	8 (0)
Chromosome number	8
Assembly length (Mb)	1,386.22
Contig N50 (Mb)	180.45
Scaffold N50 (Mb)	180.45
HiFi reads mapping rate (%)	100.00
HiFi reads coverage (%)	99.97
Number of telomeres	16
Protein-coding genes number	32,600
Repeat content (%)	64.22
Genome BUSCOs (%)	99.63
LTR assembly index	22.67
GCI score	36.23
Quality value	57.51

**AM-ONT (Xu *et al.* , 2024)**	**AM-CLR (Fan *et al* ., 2024)**
1,060 (1,032)	1,773 (1,432)
9	9
1,439.71	1,431.18
2.82	1.67
184.69	184.46
-	-
-	-
1	6
38,398	29,914
68.20	67.98
93.37	97.27
-	16.22
-	-
-	48.58

Table 2. The characteristic of centromeric regions of the AM-T2T assembly.

Chromosome	Start	End	GC content	Gene number
Chr01	123,245,059	130,176,150	37.0%	57
Chr02	30,456,263	31,781,639	39.7%	11
Chr03	124,400,000	125,180,000	39.3%	0
Chr04	93,780,950	96,286,216	38.7%	18
Chr05	54,322,074	55,622,641	39.3%	10
Chr06	86,261,760	86,613,262	39.2%	1
Chr07	43,829,593	47,989,591	39.8%	38
Chr08	51,840,000	58,080,000	40.1%	34

Repetitive sequence content (%)	Tandem repeats content (%)
84.92	34.07
84.75	51.33
97.94	85.02
92.76	57.10
84.19	49.08
96.62	77.58
84.12	43.91
83.78	37.84

DNA transposons content (%)	LTR content (%)
1.57	77.50
47.81	36.66
72.89	23.65
53.21	34.99
50.17	35.48
65.91	26.60
39.80	44.46
31.06	51.25

Figure1 Click here to access/download;Figure;Figure1.png

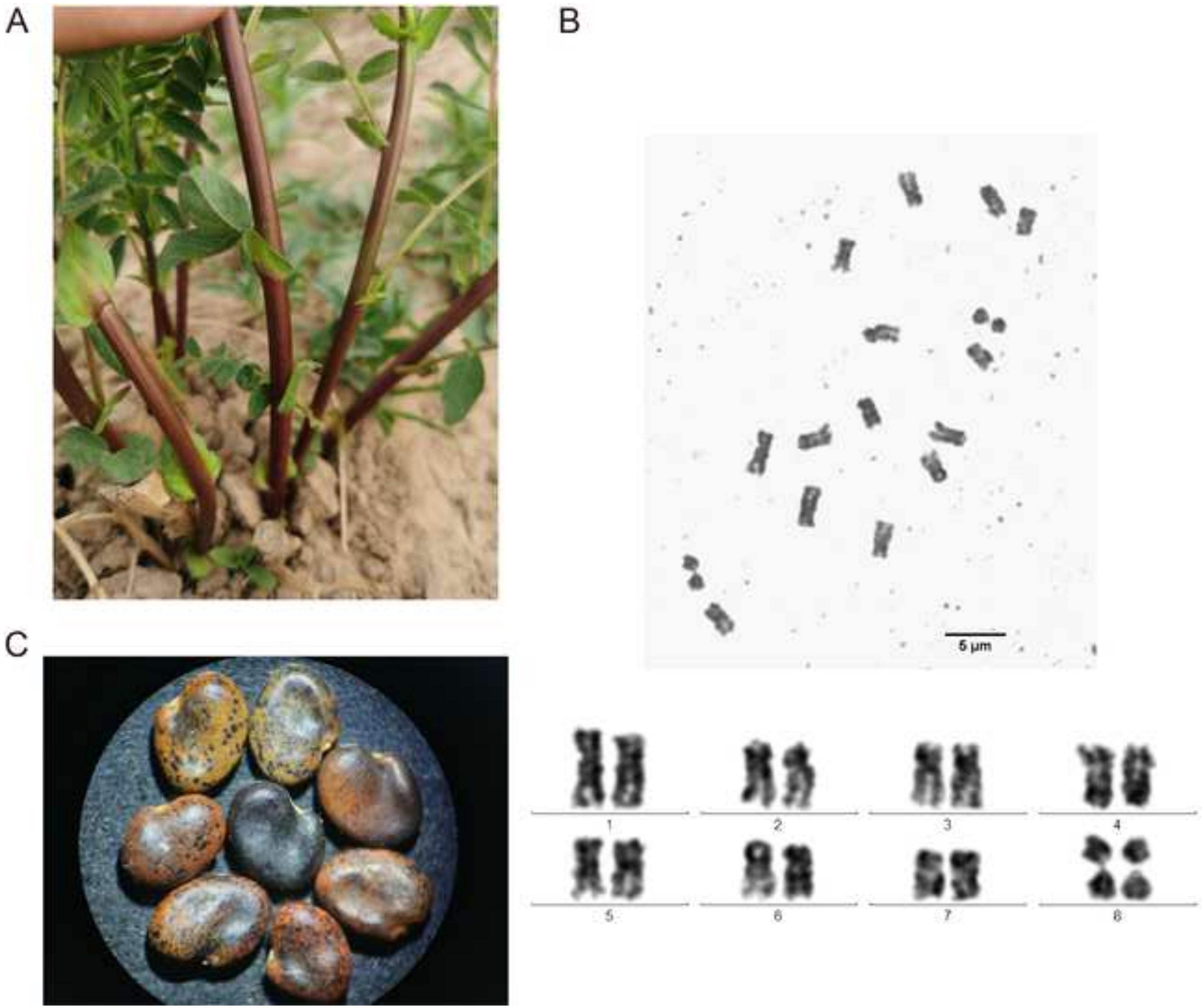

Figure2 Click here to access/download;Figure;Figure2.png

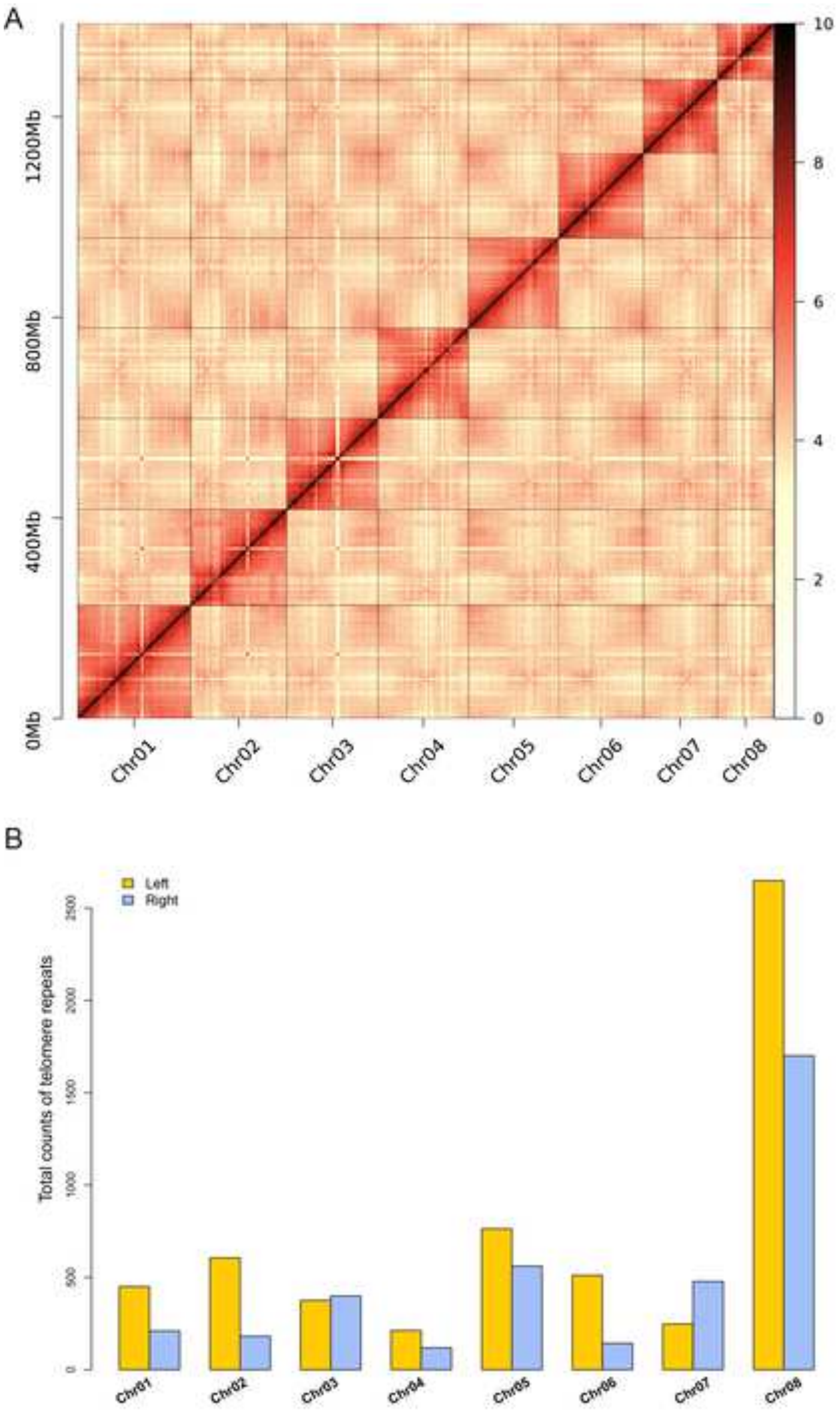

Figure3

Click here to access/download;Figure;Figure3.png

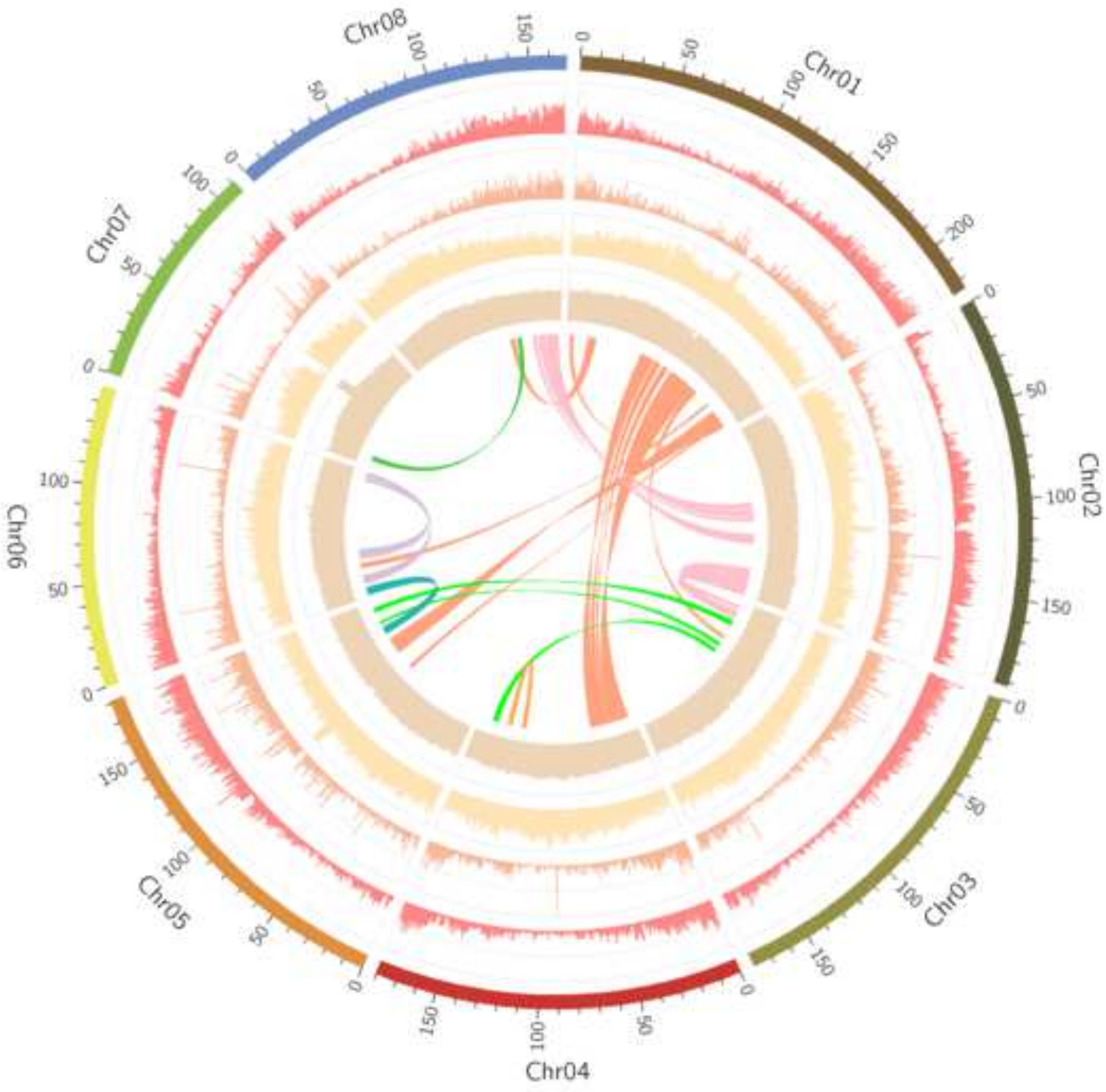

Figure4 Click here to access/download;Figure;Figure4.png

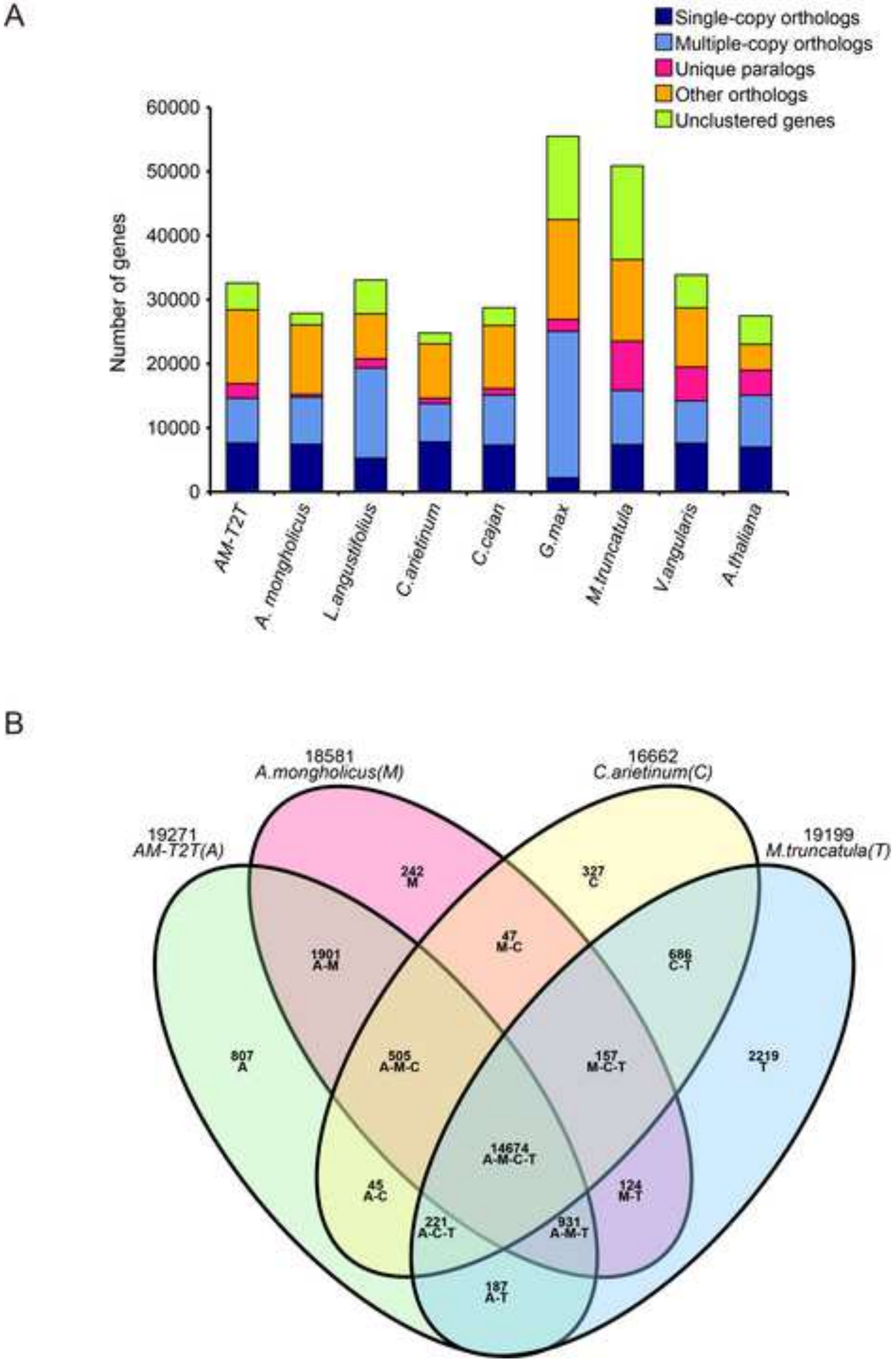

Figure5

Click here to access/download;Figure;Figure5.pdf

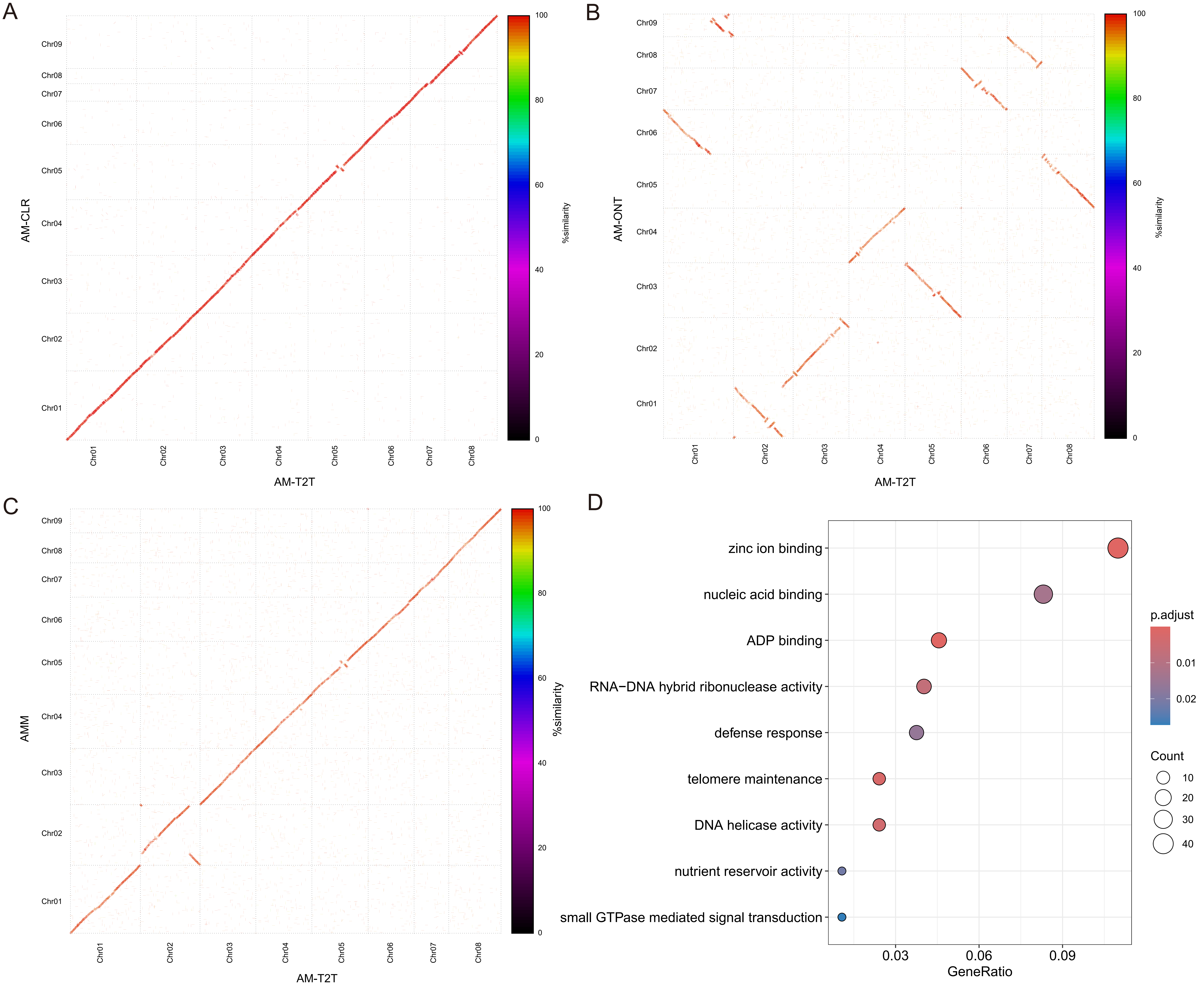

Supplementary Table

Click here to access/download
Supplementary Material
Supplementary Table.xlsx

Supplementary Figure

Click here to access/download
Supplementary Material
Supplementary Figure.docx
