# Supplementary material for: The complete genome assembly of Astragalus membranaceus: enabling more accurate genetic research
Source: Gigascience. 2025 Oct 1;14:giaf117. doi: 10.1093/gigascience/giaf117 (PMC12486382; doi:10.1093/gigascience/giaf117)
Supplement: giaf117_GIGA-D-25-00123_Revision_1 [file giaf117_giga-d-25-00123_revision_1.pdf]

GigaScience

The complete genome assembly of Astragalus membranaceus: enabling more accurate genetic research

--Manuscript Draft--

**Manuscript Number:**	GIGA-D-25-00123R1
**Full Title:**	The complete genome assembly of Astragalus membranaceus: enabling more accurate genetic research
**Article Type:**	Data Note
**Funding Information:**	Fundamental Research Program of Shanxi Province (202103021224158) — Associate Research Fellow Qin Huibin<br>the National Natural Science Foundation of China (31601457) — Associate Research Fellow Qin Huibin<br>Chinese Materia Medica, China Agriculture Research System (CARS-21) — Research Fellow Hongling Tian<br>Hengshan Astragalus Research Institute's Local Partnership Project (XDHZHQY2022-01) — Research Fellow Hongling Tian
**Abstract:**	Background: Astragalus membranaceus (Fisch.) Bunge is a globally significant medicinal plant renowned for its potent immunomodulatory and antioxidant properties. However, the existing reference genome for this species remains incomplete, characterized by fragmented assemblies and the absence of centromeric and telomeric regions, thereby limiting comprehensive exploration of the genetic mechanisms underlying its key traits.<br>Findings : We hereby present the first complete genome assembly for Astragalus membranaceus (Fisch.) Bge “AM-T2T”, generated through PacBio HiFi, ONT long reads, and Hi-C scaffolding. The assembly achieved a total size of 1.39 Gb with a N50 of 180.45 Mb. It features annotations of 64.22% repetitive sequences, 16 telomeres, 8 centromeres on 8 chromosomes, and 32,600 high-confident genes. Notably, 158.58 Mb of previously unassembled regions (PURs) were resolved, harboring 46 TFs. The assembly benefits mapping analysis for RNA-seq, outperforming two previously published genomes (AM-ONT, AM-CLR) as reference genomes. Additionally, 2,267 unique genes and 20,652 conserved genes were identified within the AM-T2T genome.<br>Conclusions : This complete genome assembly of A. membranaceus fills critical gaps in the understanding of this species and its adaptations. It represents a significant advancement in the genomic characterization of A. membranaceus, providing a robust resource that will bolster genetic research, breeding programs, and medicinal applications.
**Corresponding Author:**	Hongling Tian<br><br>CHINA
**Corresponding Author Secondary Information:**	
**Corresponding Author's Institution:**	
**Corresponding Author's Secondary Institution:**	
**First Author:**	Qin Huibin, Ph.D.
**First Author Secondary Information:**	
**Order of Authors:**	Qin Huibin, Ph.D.<br>Aohui Li<br>Shuyu Zhong<br>Huazhi Wang<br>Hongling Tian

Powered by Editorial Manager® and ProduXion Manager® from Aries Systems Corporation

**Order of Authors Secondary Information:**	
**Response to Reviewers:**	Dear editor,<br>We sincerely thank you and the reviewers for your valuable comments and instructive advice, which help us to improve and revise our manuscript (Manuscript Number: GIGA-D-25-00123, Title: The complete genome assembly of Astragalus membranaceus: enabling more accurate genetic research). We have made detailed changes accordingly. Our point-by-point responses to the reviewers' comments are provided as follows for your consideration. For ease of reviewing, all the significant changes in the revised manuscript have been highlighted in red. The list of our point-to-point responses is enclosed below with the reviewers' comments reproduced. We hope the revised manuscript can be accepted by the journal of GigaScience.<br>Thank you very much for your help!<br><br>With best regards,<br>Yours sincerely,<br><br><br>Reviewer reports:<br><br>Reviewer #1: General comments:<br>Astragalus membranaceus is a valuable Chinese medicinal plant. Here, Qin et al. reported a T2T genome assembly for A. membranaceus, which serves as an important genomic resource for future research. However, this manuscript is quite descriptive and lacks innovative findings. I have concerns about this manuscript, especially regarding the T2T assembly process, subsequent bioinformatic analysis, and the writing.<br><br>Major comments:<br>1.The authors briefly described the T2T assembly process. As the T2T assembly is the main highlight of this manuscript, I suggest the authors provide a detailed explanation of the process, especially on how the gaps are closed. For instance, they could elaborate on how the Ultra-Long data helped close these gaps, and how they filled in the remaining gaps after Ultra-Long promotion.<br>Re: Thanks for your valuable suggestions. In our revised manuscript, we added an analysis flowchart for T2T assembly to provide readers with a clearer understanding of the construction process of the T2T genome (see Supplementary Fig. 1). The resulting chromosomal-level assembly exhibits only 8 gaps, facilitated by the genome's high contiguity (Contig N50: 120.48 Mb). This low gap density offered significant advantages for subsequent T2T genome construction. During the gap-filling process, we utilized longer pre-assembled contigs and higher-accuracy HiFi data, consistent with the methods employed in the latest T2T genome assemblies of Prunus campanulata (D. Jiang et al., 2025) and Siniperca roulei (M. Jiang et al., 2025) published in GigaScience.<br><br>Reference:<br>Jiang, D., Li, Y., Zhuge, F., Zhou, Q., Zong, W., Liu, X., & Shen, X. (2025). The telomere-to-telomere genome of flowering cherry (Prunus campanulata) reveals genomic evolution of the subgenus Cerasus. GigaScience, 14. https://doi.org/10.1093/gigascience/giaf009<br>Jiang, M., Zhao, C., Ma, F., Yin, D., Wang, C., Jian, J., & Liu, K. (2025). The telomere-to-telomere gap-free reference genome and taxonomic reassessment of Siniperca roulei. GigaScience, 14. https://doi.org/10.1093/gigascience/giaf068<br><br>2.The section of "Whole genome comparative analysis" present shallow description of structure variation between the T2T and the two other published genomes. The authors did not explain why such variations exist. Does it stem from the genetic difference of different accessions of A. membranaceus? Please give more evidence and explanation here, providing biological insights for reader. The comparison with the genome of another species in the genus does not really confirm the structure variation. Is there any species identification error in this study? Is the sequenced individual AMM? In addition, lines 341-348 could be moved to the previous paragraph. To me, it indicates the improvement of the T2T assembly compared to the published ones. Thus, it could be moved to the paragraph showing the quality of the assembly.<br>Re: Thank you for this comment. As a Data Note, this manuscript focuses on the

Powered by Editorial Manager® and ProduXion Manager® from Aries Systems Corporation

technical aspects of T2T genome construction rather than biological insights. Its primary objective is to establish a foundational framework for future biological research, analogous to the recently published T2T genome of Prunus campanulata in GigaScience (D. Jiang et al., 2025). In this study, we provide the T2T genome resource, alongside performing gene annotation, identifying the CYP450 and UGT gene families (newly added), and constructing structural variants—all of which lay the groundwork for subsequent investigations. As for biological insights, we conducted a whole-genome comparative analysis between AM-T2T and AMM, identifying SV-genes in our revised manuscript. Pathway enrichment analysis of these SV-genes indicates their potential involvement in the survival, development, and environmental adaptation of A. membranaceus. Please find more details in lines 425–450 of the revised manuscript.
As you suggest, the lines 341-348 have been moved to the previous paragraph.

Reference:
Jiang, D., Li, Y., Zhuge, F., Zhou, Q., Zong, W., Liu, X., & Shen, X. (2025). The telomere-to-telomere genome of flowering cherry (Prunus campanulata) reveals genomic evolution of the subgenus Cerasus. GigaScience, 14. https://doi.org/10.1093/gigascience/giaf009

3.What is the biological explanation of the enriched GO terms of the unique genes in A. membranaceus? How does that make sense?
Re: Thanks for your comment. While our study presents broad functional enrichment results for these unique genes, their precise biological roles remain undetermined. We propose that future research should focus on identifying key regulators of saponins and flavonoid biosynthesis pathways, along with associated transcriptional networks.

4.I highly suggest the authors to at least demonstrate an example showing 'how the T2T genome enables addressing any scientific questions'. For example, are the 46 transcription factors, located in previously unassembled regions, involved in regulating the biosynthesis of any secondary metabolites that you mentioned in the first paragraph? Such a demonstration is a strong example of the value of a T2T genome.
Re: Thanks for your comment. Although this manuscript is structured as a Data Note, a genre that emphasizes analytical methodologies for constructing the T2T genome rather than addressing specific scientific questions, the value of our T2T genome and its relevance to scientific inquiry are highlighted in three key aspects:
1.High-quality genome assembly
The T2T genome demonstrates exceptional quality, supported by rigorous metrics: 99.63% BUSCO completeness, a LAI of 22.67, a quality value of 57.51, a GCI (Genome Continuity Inspector) score of 36.23, and a relatively high RNA-seq mapping rate. This superior assembly serves as a more comprehensive and accurate reference genome, empowering researchers to conduct in-depth investigations into the molecular mechanisms governing critical agronomic traits.
2.Novel insights into telomeres and centromeres
Our analysis of telomeres and centromeres establishes a foundation for subsequent cross-species evolutionary studies of centromeres, which could not have been undertaken without the T2T genome.
3.Functional implications of gene annotations
In the revised manuscript, we present an analysis of the CYP450 gene family. Among the 46 transcription factors located in PUR regions, four were identified as CYP450 genes: CYP71B38, KAO2, CYP71A13, and CYP86A1, with CYP71B38 detected in centromeric regions. Previous studies have suggested that CYP71 subfamily genes in Panax ginseng are involved in the biosynthesis of secondary metabolites, aldehydes, and flavonoids (Seitz et al., 2006). Please find more details in lines 381–400 of the revised manuscript.

Reference：
Seitz, C., Eder, C., Deiml, B., Kellner, S., Martens, S., & Forkmann, G. (2006). Cloning, Functional Identification and Sequence Analysis of Flavonoid 3′-hydroxylase and Flavonoid 3′,5′-hydroxylase cDNAs Reveals Independent Evolution of Flavonoid 3′,5′-hydroxylase in the Asteraceae Family. Plant Molecular Biology, 61(3), 365-381. https://doi.org/10.1007/s11103-006-0012-0

5.The authors did not pay enough attention to the language and the logical flow

between paragraphs. Especially, the logic in the introduction is difficult to follow. Please do consider re-organizing paragraphs in the introduction and results section. I have caught quite some improper usage of language and grammar mistakes. I highly require the authors to carefully edit the languages.
Re: Thanks for your reminding. Our revised manuscript has undergone professional editing by a native English speaker to ensure adherence to academic writing conventions, along with enhanced spelling accuracy and grammatical precision. Furthermore, we have refined the structure and content of paragraphs in both the Introduction and Results sections. For instance, we have comprehensively reviewed recent molecular-level research findings on A. membranaceus and integrated their key conclusions into the Introduction to strengthen contextual relevance.

Reviewer #2: The manuscript presents a presents the first high-quality telomere-to-telomere genome assembly of A. membranaceus, and identified 16 telomeres and 8 centromeres distributed across 8 chromosomes. Additionally, 158.58 Mb of PURs, 2,267 unique genes, and 20,652 conserved genes were identified. Genome-wide comparison identified significant structural variations compared with prior assemblies. I read this manuscript with HUGE interests，but still have several concerns need to be clarified.
Re: Thank you for your comments and assistance. We believe that the latest updated version has addressed all the issues you mentioned.

Major:
1、I think the manuscript lacks an analysis linking the medicinal properties of Astragalus membranaceus to its T2T genome. For key genes involved in the synthesis pathways of important compounds like astragaloside and polysaccharides (such as CYP450 and UGT), it remains unclear whether they are located in previously unassembled centromeric regions. We recommend conducting and including an analysis of key genes in the synthesis pathways of important compounds.
Re: Thanks for your valuable suggestions. The CYP450 and UGT gene families have been identified in our revised manuscript. Four CYP450 genes (CYP71B38, KAO2, CYP71A13, and CYP86A1) were localized to the PUR region, with CYP71B38 detected in centromeric regions. No UGT genes localized to either the PUR or centromeric regions. However, phylogenetic analysis revealed significant contraction of the UGT76 gene family and expansion of the UGT73 gene family in the AM genome relative to A. thaliana. Please find more details in lines 380–400 of the revised manuscript.

2、The authors mention that "898 genes were annotated within these PURs, including 46 transcription factors." It is unclear whether these TFs represent newly discovered families or whether they exhibit specific expression in medicinal parts such as roots and leaves. We suggest supplementing the analysis with RNA-seq tissue - specific expression heatmaps.
Re: Thanks for your suggestions. First, the expression profiles of all 898 genes within the PUR region have been added to Supplementary Table S13. Second, we have conducted heatmap visualization, tissue-specific expression analysis, and differential expression analysis for the CYP450 and UGT gene families. For further details, please refer to lines 380–400 of the revised manuscript.

3、In Figure 5, part A of the comparative genomics section does not clearly show the differences between AM-T2T and AMM-CLR. We recommend a more refined collinearity analysis, such as illustrating gene presence / absence, inversions, and other variations for each chromosome across the three genomes (AM-T2T, AM-CLR, and AM-ONT).
Re: Thanks for your comment. We have refined the manuscript’s structure and logical flow, with a particular focus on contrasting structural variations (SVs) between the AM-T2T and AMM. The core objective of comparing multiple A. membranaceus genome assemblies is to validate the accuracy and integrity of the T2T genome. To corroborate the precision and comprehensiveness of our T2T assembly, we performed a series of rigorous validation analyses, including BUSCO assessment, quality value (QV) calculation, mapping rate analysis, LAI (LTR Assembly Index) evaluation, GCI (Genome Continuity Inspector), and CRAQ (Clipping information for Revealing Assembly Quality) analysis. Our comparative framework prioritizes contrasts between the AM-T2T and AMM genomes, thereby highlighting the advantages of the T2T

genome in SV identification. Moving forward, we aim to identify specific SVs associated with the biosynthesis of astragalosides and flavonoids, which will be visualized for readers via dedicated structural variation diagrams. Please find more details in lines 425–450 of the revised manuscript.

Minor

1、The body paragraphs of the manuscript currently employ a hanging indent at the beginning of some paragraph. Please carefully revise the text to ensure paragraphs are formatted without indentation at the start.
Re: Revised.

2、Regarding lines 166-168: The tools and database names mentioned in this section appear to have formatting inconsistencies. Software tools such as "Next Denovo" ,"Repeat Masker", "Repeat Protein Mask," "Repeat Modeler", and "Repeats Finder" should follow standard naming conventions (e.g., RepeatMasker, RepeatProteinMask, RepeatModeler, and RepeatsFinder, written as single terms without spaces). Additionally, the database "rebase" should be capitalized as RepBase. For clarity and reproducibility, we recommend adding official website links for these tools (e.g., RepeatMasker: http://www.repeatmasker.org).
Re: Revised.

3、In line 176, the phrase "yielded 221,161 transcripts with a N50 size of 1,636" omits the unit for the N50 value. Please add the appropriate unit (e.g., bp) to ensure clarity and adherence to scientific reporting standards (revise to "N50 size of 1,636 bp").
Re: Revised.

4、In lines 196-197, the description of FPKM (Fragments Per Kilobase Million) calculation lacks sufficient detail. Please elaborate on the computational workflow, including the specific software or tools used (e.g., Cufflinks, StringTie, or custom scripts), parameters applied, and normalization steps. This will enhance reproducibility and methodological transparency.
Re: The analysis software and parameters for gene expression levels have been detailed. Please find more details in lines 231–238 of the revised manuscript.

5、In line 201, Arabidopsis thaliana is written in full. Per taxonomic conventions, after the first mention, the genus name should be abbreviated (e.g., A. thaliana). Please revise subsequent instances accordingly.
Re: Revised.

6、In line 208, the sentence "we used quarTeT (RRID:SCR_025258) version 1.1.5 TeloExplorer to identify telomeres" conflates two software tools without clarifying their relationship. To avoid confusion, please rephrase to specify whether quarTeT and TeloExplorer are integrated modules or separate tools used in tandem. For example: "we used quarTeT (RRID:SCR_025258, version 1.1.5) with TeloExplorer to identify telomeres."
Re: Revised.

7、The "Functional Annotation" section lacks critical methodological details. Please specify the databases or tools used for annotation (e.g., InterProScan, EggNOG, KEGG, or GO), the parameters applied, and the criteria for assigning functional categories. This omission hinders reproducibility and clarity.
RE: Thanks for your comment. The detailed method of functional annotation was added. Please find more details in lines 225–230 of the revised manuscript.

8、In lines 218-220, the description of the concatenated phylogenetic tree construction is incomplete. Please clarify the concatenation strategy (e.g., supermatrix approach).
RE: Thanks for your comment. The description of “The alignments were concatenated and converted into a super-gene alignment in Phylip format” have been added. Please find more details in lines 275–276 of the revised manuscript.

9、In lines 230-231, the sentence "Using different data, contigs were constructed with four assembly tools, including Hifiasm, Wtdgb2, Flye, and NextDenovo" belongs to the Methods section. Please avoid reiterating methodological steps in the Results and

	focus instead on presenting key findings.<br>Re: Revised. The sentence “Using different data, contigs were constructed with four assembly tools, including Hifiasm, Wtdgb2, Flye, and NextDenovo.” have been move to the methods section. At the same time, the language in this paragraph was polished and modified.<br><br>10、In line 310, In line 310, the presentation of results lacks specific data. It is recommended to include a table with the expression data of the genes.<br>Re: Revised. The gene expression matrix has been provided in Supplementary Table S15.<br><br>11、In line 316, the Latin names in "A.mongholicus, C.arietinum, and M.truncatula" lack spacing between the genus abbreviation and the species epithet (e.g., "A. mongholicus" instead of "A.mongholicus"). Ensure all Latin names follow the standard format (Genus abbreviation + space + species*, italicized).<br>Re: Revised.<br><br>12、For the phylogenetic tree in Figure S6: Add branch support values (e.g., bootstrap percentages, posterior probabilities) to key nodes. Highlight focal species or clades of interest using distinct colors or symbols to improve visual clarity.<br>Re: Revised. Bootstrap values have been added. Please find more details in Figure S8.<br><br>13、In lines 317-319, the phrase "out of the 19,271..." lacks context regarding the comparison species. Revise to explicitly state the reference group (e.g., "Compared to [species name], out of the 19,271...").<br>Re: This study does not include comparisons to the most recent common ancestor for contraction/expansion inference, as the scope is limited to gene family enumeration and phylogenetic placement. If it is an analysis of gene family contraction and expansion, according to your suggestion, "Compared with the most recent common ancestor" should be added.<br><br>14、Ensure genus abbreviations in Figure 4 (e.g., "A.mongholicus") include a space after the period (e.g., "A. mongholicus"). The label "AM-T2T" in Figure 4B should not be italicized, as it refers to an assembly name rather than a taxonomic term.<br>Re: Revised. Due to the addition of analysis of the CYP450 and UGT gene families, the original Figure 4 has been changed to Figure 5 in our revised article.<br><br>15、The manuscript lacks a description of the comparative genomic analysis methods and it is recommended to supplement this information.<br>Re: The method of "Identification of structural variants between AM-T2T and AMM", and “Identification of PUR regions”, has been added in our revised manuscript. Please find more details in lines 281–289 of the revised manuscript.
**Additional Information:**	
**Question**	**Response**
Are you submitting this manuscript to a special series or article collection?	No
**Experimental design and statistics**<br><br>Full details of the experimental design and statistical methods used should be given in the Methods section, as detailed in our Minimum Standards Reporting Checklist. Information essential to interpreting the data presented should be made available in the figure legends.	Yes

Powered by Editorial Manager® and ProduXion Manager® from Aries Systems Corporation

Have you included all the information requested in your manuscript?	
**Resources**<br><br>A description of all resources used, including antibodies, cell lines, animals and software tools, with enough information to allow them to be uniquely identified, should be included in the Methods section. Authors are strongly encouraged to cite Research Resource Identifiers (RRIDs) for antibodies, model organisms and tools, where possible.<br><br>Have you included the information requested as detailed in our Minimum Standards Reporting Checklist?	Yes
**Availability of data and materials**<br><br>All datasets and code on which the conclusions of the paper rely must be either included in your submission or deposited in publicly available repositories (where available and ethically appropriate), referencing such data using a unique identifier in the references and in the “Availability of Data and Materials” section of your manuscript.<br><br>Have you have met the above requirement as detailed in our Minimum Standards Reporting Checklist?	Yes
GigaScience has policies and guidelines in place for the use of generative AI-writing tools such as ChatGPT. If you have used such writing tools to assist with writing the manuscript this must be declared and cited in the text. Authors should not list AI-writing tools and other AI-assisted technologies as an author or co-author and should acknowledge that they are fully responsible for text generated or refined by AI-writing tools.<p><br><br>A summary of use (particularly in the introduction or among methods) needs to	No

be included at the end of the paper, and the outputs should also be included as a supplementary file hosted in GigaDB or other open repositories. Please read our guidelines for more information. <p><br><br>By submitting to GigaScience, you are aware of the journal's AI-writing tools policy, and if you have declared use of such tools below, you have acknowledged this where appropriate in your manuscript and have made a summary of use and outputs available. <p><br><b>AI-assisted writing tools have been used in the preparation of this manuscript?	

Manuscript
Click here to access/download;Manuscript;Revised_Manuscript-Aug

DATA NOTE

The complete genome assembly of *Astragalus membranaceus*: enabling more accurate genetic research

Huibin Qin [1*†], Aohui Li [2†], Shuyu Zhong [2†], Huazhi Wang [2†], Hongling Tian [3*]

1 Center for Agricultural Genetic Resources Research, Shanxi Agricultural University/ Institute of Crop Germplasm Resources, Shanxi Academy of Agricultural Sciences, Key Laboratory of Crop Gene Resources and Germplasm Enhancement on Loess Plateau, Ministry of Agriculture, Shanxi Key Laboratory of Genetic Resources and Genetic Improvement of Minor Crops, Taiyuan 030031, China

2 College of Agronomy, Shanxi Agricultural University, Taigu 030801, China

3 Shanxi Agricultural University, Shanxi Academy of Agricultural Science, The Industrial Crop Institute, Taiyuan 030031, China

*Correspondence address.

Huibin Qin, China. E-mail: nkyqhb@163.com.

Hongling Tian, China. E-mail: thl2003@163.com.

[†] These authors contributed equally to this work.

Qin Huibin [0000-0003-2229-6482] Hongling Tian [0009-0000-7392-0260]

Abstract

Background: *Astragalus membranaceus* (Fisch.) Bunge is a globally significant medicinal plant renowned for its potent immunomodulatory and antioxidant properties. However, the existing reference genome for this species remains incomplete, characterized by fragmented assemblies and the absence of centromeric and telomeric regions, thereby limiting comprehensive exploration of the genetic mechanisms underlying its key traits.

Findings: We hereby present the first complete genome assembly for *Astragalus membranaceus* (Fisch.) Bge "AM-T2T", achieved through the integration of PacBio HiFi, ultra-long Oxford Nanopore Technologies, and Hi-C sequencing. The assembly achieved a total size of 1.39 Gb with a N50 of 180.45 Mb. The genome exhibits remarkable completeness (99.63% BUSCO completeness; LAI of 22.67) and high accuracy (quality value of 57.51; GCI score of 36.23). It features annotations of 64.22% repetitive sequences, 16 telomeres, 8 centromeres, 32,600 high-confident genes, 248 cytochrome P450 monooxygenases (CYP450s), and 163 uridine diphosphate glycosyltransferases (UGTs). Notably, 158.58 Mb of previously unassembled regions (PURs) were resolved, harboring four CYP450s. Additionally, 2,267 unique genes and 20,652 conserved genes were identified within the AM-T2T genome. Comparative analysis with *Astragalus mongholicus* assembly revealed 1,413 structural variations.

Conclusions: This complete genome assembly of *A. membranaceus* represents a significant advancement in the genomic characterization of *A. membranaceus*, providing a robust resource that will bolster genetic research, breeding programs, and medicinal applications.

Key words: *Astragalus membranaceus*, telomere-to-telomere genome, genome annotation,

previously unassembled regions, structural variations

Data Description

Context

Astragalus membranaceus (Fisch.) Bunge (NCBI:txid649199), a key species in the Fabaceae family, has served as a fundamental component of traditional pharmacopeias across Asian civilizations for millennia. Its dried roots, known as "Huangqi" or *Astragali Radix,* have been utilized for over thousands of years in traditional Chinese medicine [1]. This medicinal plant accumulates diverse bioactive compounds, including flavonoids, triterpenoids, polysaccharides, and amino acids. Flavonoids not only participate in plant defense against biotic and abiotic stresses but also exhibit significant antioxidant and anti-inflammatory activities beneficial to human health [2-4]. Triterpenoids such as astragalosides have demonstrated pharmacological effects like enhancing immunity, protecting the cardiovascular system, and exhibiting anti-tumor properties [5, 6]. Recent studies have further expanded our understanding of its therapeutic potential. Yu *et al.* reported that *A. membranaceus* attenuates peritoneal fibrosis in both *in vivo* and *in vitro* models by suppressing DNA methyltransferase activity [7]. This effect correlated with improved histopathology, reduced mesothelial-mesenchymal transition biomarkers, and downregulated DNMT1/DNMT3a expression. Mechanistically, it alleviates fibrosis *via* Dnmt3a-mediated epigenetic regulation of ID2 promoter methylation through the PI3K/Akt pathway, establishing DNA methylation as a critical target for suppressing fibrogenesis. Additionally, Shenshuaikang enema, a formulation derived from *A. membranaceus*, has shown significant efficacy and safety in treating chronic kidney disease by restoring intestinal barrier function via regulation of the

microbiota-gut-kidney axis [8]. Astragenol, another bioactive component, alleviates neuroinflammation and ameliorates Parkinson's disease symptoms through modulation of amino acid metabolism and inhibition of ferroptosis [9]. Such multifaceted biological activities and therapeutic properties have established *A. membranaceus* as a valuable resource for drug discovery and functional food development, driving the need for deeper insights into its genetic and metabolic mechanisms.

Recent advances in genomics have deepened our understanding of *A. membranaceus* biology. Zhang *et al*. decoded the first complete mitochondrial genome, revealing a multi-chromosome structure and providing insights into the evolutionary mechanisms of this medicinal plant [10]. Li *et al*. reconstructed the full-length transcriptome using PacBio Iso-Seq technology, identifying numerous transcript variants involved in the biosynthesis of bioactive compounds such as astragalosides and calycosin [11]. Furthermore, Wang *et al.* identified 76 WRKY transcription factors (TFs) in *A. membranaceus*, with AmWRKY8 conferring drought resistance through hormonal signaling and nuclear-localized transcriptional activation [12]. Concurrently, AmMYB35 was shown to upregulate flavonol biosynthesis under drought stress [13]. At the metabolic engineering level, direct injection of Rhizobium rhizogenes carrying an AmUGT15 overexpression cassette into *A. membranaceus* stem explants induced hairy roots with significantly enhanced astragaloside biosynthetic capacity [14].

De novo genome assembly is a fundamental and powerful tool in genomics research. Recently, two *A. membranaceus* genome assemblies have been developed using different sequence platforms: One 1.43 Gb assembly (AM-CLR) was generated using Pacific Biosciences (PacBio) continuous long reads (CLR) and chromatin conformation capture (Hi-C) technology, achieving a contig N50

of 1.67 Mb [15]. Another 1.47 Gb chromosome-level genome assembly (AM-ONT) employed MGI-SEQ short-read, Oxford Nanopore (ONT) long-read and Hi-C technologies (AM-ONT) [16]. Additionally, the genome of *Astragalus mongholicus* (AMM), another authorized plant source of Astragali Radix, has been decoded [17]. However, these assemblies remain incomplete in repetitive sequence regions, centromeres, and telomeres**,** limiting comprehensive understanding of genetic mechanisms governing bioactive compound biosynthesis and key agronomic traits.

Telomere-to-telomere (T2T) assembly represents a critical advancement for deciphering complex genomes. Recent technological breakthroughs have made T2T genome assembly feasible, enabling comprehensive genome identification. PacBio high-fidelity (HiFi) sequencing generates highly accurate long-read datasets with mean read lengths of 10–25 kb and >99.9% base accuracy. The primary determinant of assembly complexity is not the size of the genome, but the repetitive sequence. ONT has addressed this challenge through ultra-long-read methods producing reads averaging ~50 kb (with lengths exceeding 100 kb), overcoming limitations posed by repetitive regions refractory to HiFi assembly. Leveraging these methodologies, T2T genomes have been achieved for staple crops such as rice [18], maize [19], and sorghum [20]. Methodological frameworks for T2T assembly have also been systematically reviewed, providing robust guidance for genomic studies of medicinal plants [21, 22].

Therefore, to address the genomic gaps in *A. membranaceus,* we integrated PacBio HiFi sequencing, ONT ultralong sequencing, and Hi-C technology to construct its T2T genome assembly. This enabled, for the first time, comprehensive characterization of telomeric and centromeric regions. Leveraging this T2T genome, we identified PUR regions and annotated genes within these regions. Furthermore, genome-wide investigations were performed for CYP450s and

UGTs, while unique and conserved gene sets were systematically analyzed. This T2T genome assembly marks a significant advancement in *A. membranaceus* genomics, providing a solid foundation for diverse downstream comparative genomic analyses and pan-genome studies.

Methods

Sample collection

The study materials (SXHQ0000254) used in this study was collected from the Zhengyao Garden of Gansu University of Traditional Chinese Medicine (Fig. 1A; Fig. 1C). The voucher specimen is currently deposited in the medicinal plant experimental field of the Fenyang Economic Research Institute, located in Lvliang City, Shanxi Province. High-quality genomic DNA was extracted from healthy young leaves. All samples were frozen in liquid nitrogen and stored at -80°C for preservation and subsequent analysis.

Karyotype analysis

Root tips were excised from germinated seeds and pretreated in a solution containing 0.001 mol/L 8-hydroxyquinoline and 0.02% colchicine (1:1, v/v) at 4°C for 4 hours to synchronize mitotic cells at metaphase. After thorough rinsing, samples were fixed in freshly prepared Carnoy’s fixative (methanol:acetic acid, 3:1, v/v) for 4–24 hours and hydrolyzed in 1 mol/L HCl at 60°C for 10 minutes. To improve cell wall digestion, enzymatic treatment with a cellulase-pectinase mixture (6%:4%, 2:1, v/v) was performed at 37°C for 5–6 hours, followed by a low-osmotic treatment in distilled water at 37°C for 30 minutes. Chromosomes were stained with carbol fuchsin, and slides were prepared using a standardized squash protocol. Chromosome images were captured using a

Nikon 80i microscope equipped with a cold CCD camera, and karyotype parameters were analyzed using Zeiss Karyotype software.

Sequencing and filtering

For HiFi sequencing, SMRTbell target size libraries were constructed according to PacBio's standard protocol (Pacific Biosciences) using about the 16-kb preparation solutions with the SMRTbell Express Template Prep Kit 2.0. The sequencing was conducted in HiFi mode on the PacBio Revio platform (RRID:SCR_017990) at BGI Genomics. The PacBio HiFi reads, initially generated in BAM format, were converted to FASTQ format using the bam2fastq tool (version 1.0.0) [17]. For ONT sequencing, ONT ultra-long insert libraries were obtained using the Oxford Nanopore SQK-LSK109 kit and sequenced on the PromethION (RRID:SCR_017987) platform The ONT data underwent processing using NanoFilt version 2.8.020 (RRID:SCR_016966) [23] with a quality threshold of 7. Previous studies of AM-CLR provided RNA-seq and Hi-C reads for supplementary analysis.

Genome assembly and Hi-C scaffolding

We use a comprehensive strategy for T2T assembly (Supplementary Fig. S1). Four assembly tools were employed to generate contigs using diverse datasets: Hifiasm version 0.19.9 (RRID:SCR_021069) [24], Wtdgb2 version 2.5 (RRID:SCR_017225), Flye version 2.9.4 (RRID:SCR_017016) [25], and NextDenovo version 2.5.2 (RRID:SCR_025033) [26]. Specifically, ultra-long ONT reads were processed using NextDenovo version 2.5.2 (RRID:SCR_025033) [26], while HiFi reads were assembled into contigs using Flye version 2.9.4 (RRID:SCR_017016) [25]

and Wtdbg2 version 2.5 (RRID:SCR_017225) [27]. The backbone contigs were generated with Hifiasm version 0.19.9 (RRID:SCR_021069) [24] using the command: " hifiasm -o AM.asm -t 50 --ul-cut 1000 --n-hap 2 --telo-m TTTAGGG --h1 $HiC_fq1 --h2 $HiC_fq2 --ul $ont $hifi". Hi-C reads (accession number: SRR27790545) were utilized to anchor contigs into chromosomes via Haphic version 1.0.6 [28]. An additional error correction step was carried out with Juicebox version 2.13.07 (RRID:SCR_021172) [29] according to the interaction signal. Contigs obtained by NextDenovo version 2.5.2 (RRID:SCR_025033) [26], Flye version 2.9.4 [25], and Wtdbg2 version 2.5 (RRID:SCR_017225) [27] were used for filling gaps with quartet_gapfiller.py script from quarTeT version 1.1.1 [30]. As recommended, the specific parameters used were “-f 5000 -l 1000 -i 40 -m 1000000 -t 20”. Remaining gaps in assembled chromosomes were closed using LR_Gapcloser (RRID:SCR_017021) [31] program with HiFi reads, following the T2T assembly protocol for sorghum [20]. To enhance genome quality, Winnowmap version 2.03 (RRID:SCR_025349) [32] was used to align HiFi reads to the chromosomes, followed by filtering to exclude secondary alignments and excessive clipping with the 'falconc bam-filter-clipped' tool. Finally, Racon version 1.5.0 (RRID:SCR_017642) [33] was performed for further polishing with the filtered alignments.

The completeness of the genome assembly was assessed utilizing Benchmarking Universal Single-Copy Orthologs (BUSCO) version 5.4.3 (RRID:SCR_015008) [34] with the embryophyta_odb10 database, which included 1,614 orthologs. The quality value (QV) was evaluated by Merqury program version 1.3 (RRID:SCR_022964) [35] with 17-mer. Long reads from ONT and HiFi were aligned to the assembly with Minimap2 version 2.24-r1122 (RRID: SCR_018550) [36]. After identifying LTR structures and using complete LTR elements to calculate

the LTR assembly index (LAI) value, we performed calculations to determine the genome assembly integrity, which was quantified using the LAI score [37]. In addition, the Genome Continuity Inspector (GCI) was assessed using GCI version 1.0 [38]. At last, a new reference-free tool, Clipping information for Revealing Assembly Quality (CRAQ), was employed to scan the regions of low quality in the genome assembly [39].

Identification of PUR regions

The AM-CLR and AM-ONT genomes were aligned to the AM-T2T assembly using Winnowmap version 2.03 (RRID:SCR_025349) [32] and Minimap2 version 2.24-r1122 (RRID:SCR_018550) [36], respectively. The parameters employed for Winnowmap and Minimap2 were as follows: -ax asm20 -t 20 -H –MD $ref $qurey > out.sam. The alignment SAM file was converted to PAF format using paftools.js with the command: paftools.js sam2paf -p out.sam > out.paf. Sequence regions in the AM-T2T assembly that remained uncovered (with mapping quality [MAPQ] > 0) were extracted using the following commands: cat out.paf |awk '{if ($12 > 0) print $6"\t"$8"\t"$9}' | bedtools sort -i - |bedtools merge -i - |bedtools complement -i - -g chr.len > out.pur.region

Genome annotations

The content of repetitive sequences in the AM-T2T was predicted using homology searching and the *ab initio* prediction method. For homology-based prediction, RepeatMasker version 4.0.7 [40] and RepeatProteinMask version 4.0.7 were used to search against RepBase. For *ab initio* prediction, LTR_FINDER version 1.07 (RRID:SCR_015247) [41] and RepeatModeler version 1.0.8 were

carried out. Tandem Repeats Finder version 4.10 (RRID:SCR_022193) [42] was used to identify the tandem repeat elements.

The gene prediction process employed a comprehensive strategy that integrated transcriptome-based, and homology-based methods. Initially, RNA-seq clean reads were assembled using Trinity version 2.8.5 (RRID:SCR_013048) [43], with the parameters '--max_memory 200 G --CPU 40 --min_contig_length 200 --genome_guided_bam merged_sorted.bam --full_cleanup --min_kmer_cov 3 --min_glue 3 --bfly_opts '-V 5 --edge-thr=0.1 --stderr' --genome_guided_max_intron 10000', yielded 221,161 transcripts with a N50 size of 1,636 bp. The assembled transcripts were then aligned to the assembly using Program to Assemble Spliced Alignment (PASA) version 2.4.1 (RRID:SCR_014656) [44], generating gene structures from valid transcript alignments (PASA-set). Additionally, RNA-seq clean reads were mapped to the assembly via Hisat2 version 2.0.1 (RRID:SCR_015530) [45]. Subsequently, Stringtie version 1.2.2 (RRID:SCR_016323) [46] and TransDecoder version 5.7.1 (RRID:SCR_017647) were employed to assemble the transcripts and identify candidate coding regions, resulting in the creation of gene models (Stringtie-set). Homologous genomes from seven assemblies, including AM-CLR, AMM [17], *Arabidopsis thaliana* Col-PEK [47], *Glycine max* (ZH13-T2T) [48], *Trifolium pratense* (ensembl release-59), *Phaseolus vulgaris* (ensembl release-59), and *Medicago truncatula* (ensembl release-59) were downloaded and used as queries to search against the assembly using GeMoMa version 1.9 (RRID:SCR_017646) [49]. These homology predictions were referred to as "Homology-set". The gene models from these three sources were subsequently merged using EvidenceModeler version 2.1.0 (RRID:SCR_014659) [50], with different weight parameters assigned to evidence from different sources (10 for Homology-set, 5 for Stringtie-set, and 5 for

PASA-set). Finally, the generated gene models underwent further refinement with PASA version 2.4.1 [44] to obtain untranslated regions and alternative splicing variation information. The integrated gene set was translated into amino-acid sequences and annotated using the method described the telomere-to-telomere genome assembly of sorghum [20]. Diamond version 0.9.30 (RRID:SCR_009457) [51] with an E-value cutoff of 1e-05 was used to compare the protein against four public databases, including NCBI non-redundant protein sequence database, SwissProt [52], Kyoto Encyclopedia of Genes and Genomes (KEGG) [53], Translation of European Molecular Biology Laboratory. Gene ontology (GO) terms of these genes were identified using InterProScan version 5.59-91.0 (RRID:SCR_005829) [54].

Gene expression analysis was conducted using the fragments per kilobase of transcript per million mapped reads (FPKM) method, following the approach applied in the blister beetle transcriptome study [55]. Briefly, RNA-seq clean reads were mapped to the reference genome using Bowtie2 version 2.5.4 (RRID:SCR_016368) with the following parameters: -q --phred33 --sensitive --dpad 0 --gbar 99999999 --mp 1,1 --np 1 --score-min L,0,-0.1 -I 1 -X 1000 --no-mixed --no-discordant -p 8 -k 200 [56]. Gene expression levels were subsequently quantified using RSEM version 1.3.3 (RRID:SCR_000262) with default parameters [57]. Differential gene expression (DGE) analysis was performed using the DESeq2 version 1.46.0 (RRID:SCR_015687) [58]. TF prediction was implemented following the method described in the eggplant genome study [59]. Transfer RNAs (tRNAs) and ribosomal RNAs (rRNAs) were predicted using tRNAscan-SE version 1.3.1 (RRID:SCR_008637) [60] and BLASTN (RRID:SCR_001598; *E*-value ≤ 1e–05) against the rRNA sequences of both *A. thaliana* and *Oryza sativa*, respectively. Both microRNAs

(miRNAs) and small nuclear RNAs (snRNAs) were identified by searching against the Rfam database (RRID:SCR_010835, release 12.0) using Infernal version 1.1.1 (RRID:SCR_010835).

Telomere and centromere identification

Following a method similar to that described in the study of the complete broomcorn millet assembly [61], we used quarTeT (RRID:SCR_025258) version 1.1.5 with TeloExplorer to identify telomeres and CentroMiner to identify centromeres [30]. Given the complex structure of centromeres, we further employed Centromics (RRID:SCR_025253) to identify centromeres by detecting high-copy tandem repeats from HiFi sequencing data.

Genome-wide identification of CYP450s and UGTs

To identify full-length CYP450 candidates in the AM-T2T genome and AMM genome, we employed the PF00067 hidden Markov model (HMM) profile from InterPro. The HMMER version 3.4 was utilized for candidate extraction with parameters: -E 1e-5, followed by length filtration (exclusion of sequences with <400 or >600 amino acids) [62]. *A. thaliana* CYP450 reference sequences were acquired from the CYP450 database [63]. Multiple sequence alignments were generated with MAFFT version 7.526, and non-conserved regions were filtered using trimAl version 1.4.1 [64, 65]. Phylogenetic trees of CYP450 genes were constructed using IQ-TREE2 version 2.3.6 with the parameters: -nt AUTO -bb 1000 -pre iqtree [66].

For UGT identification, we applied the same pipeline used for CYP450s. The UGT HMM profile (PF00201) was retrieved from InterPro, and *A. thaliana* UGT reference sequences were

obtained from the Plant UGTs database [67]. Only genes with amino acid lengths between 350–600 are retained.

Gene families analysis

Protein sets of nine species (*A. mongholicus, M. truncatula, A. thaliana, Cicer arietinum, Cajanus cajan, G. max, M. truncatula, Lupinus angustifolius, Vigna angularis,* and AM-T2T) were employed in the orthology identification with *A. thaliana* as the out-group. The OrthoMCL version 2.0.9 (RRID:SCR_007839) was applied to determine and cluster gene families among these nine plant species. A total of 1,153 single-copy orthologs among these species were multiply aligned with Muscle version 3.8.1551 (RRID:SCR_011812). The alignments were concatenated and converted into a super-gene alignment in Phylip format and then used for constructing a phylogenomic tree using IQtree2 version 2.3.6 with parameters of "-B 1000 -m MFP". Gene Ontology (GO) enrichment was conducted using ClusterProfiler version 4.2.2 (RRID:SCR_016884) to explore the functional characteristics of the unique gene families in the AM-T2T genome.

Identification of structural variants between AM-T2T and AMM

Genome alignment between the AM-T2T and AMM genomes was performed using the NUCmer program of MUMmer4 version 4.0.0rc1 (RRID: SCR_018171) [68] with parameter settings: --mum -g 1000 -c 90 -l 40. Subsequently, the delta-filter program was employed to identify alignment blocks using the parameters: -r -q -l 1000. Structural variants (SVs) larger than 50 bp were detected using Assemblytics based on the filtered results. A gene was designated as an "SV-gene" if at least 30% of its regulatory regions (defined as ±2 kb flanking sequences in this study) or coding sequence

(CDS) overlapped with an SV. KEGG pathway analysis was performed using KOBAS version 2.0.12 [69].

Results

Complete genome assembly and completeness evaluation

The somatic chromosome complement of *A. membranaceus* displayed a diploid constitution of 2n = 2x = 16, consistent with previous cytogenetic investigations on this species and its variants [70, 71] (Fig. 1B).

We newly sequenced the genome of *A. membranaceus*, generating 92.74 Gb (~66.72 × coverage) of PacBio HiFi reads, and 38.53 Gb (~ 27.72 × coverage) of ONT reads (Supplementary Table S1). Four distinct software packages were employed for genome assembly based on different data types. Among these, Hifiasm produced the most contiguous genome assembly using the mixed dataset, with a contig N50 of ~120.48 Mb and the fewest contig sequences (Supplementary Table S2). Thus, this assembly served as the backbone for scaffolding contigs, while contigs from the other assemblies were used for downstream gap-filling analysis. As a result, a total of 1.41 Gb of Hifiasm assembly sequences were anchored to eight pseudochromosomes, with eight gaps distributed across six of the pseudochromosomes (Supplementary Table S3). One gap was closed using pre-assembled contigs, and the remaining seven gaps were filled using PacBio HiFi reads. After further polishing, a gap-free reference genome designated as AM-T2T was generated, containing a total length of 1.39 Gb (Table 1).

To assess the accuracy and completeness of the AM-T2T assembly, various methods were employed. First, PacBio HiFi reads were mapped onto the genome, yielding a 100% mapping rate

and 99.97% genome coverage (Table 1). In particular, the CRAQ analysis revealed that only 0.02% of the genome was classified as low-confidence (Supplementary Table S4). Within these low-confidence regions, repetitive sequences accounted for 72.60%, significantly higher than the 64.22% of repetitive sequences in the entire genome, which may be the primary reason for their low-confidence classification. Second, the BUSCO analysis demonstrated that the completeness of the AM-T2T genome reached 99.63%, exceeding those of the AM-ONT and AM-CLR assemblies (Table 1). Notably, the RNA-seq mapping analysis revealed that AM-T2T was more suitable for analyzing RNA-seq data, with higher mapping rates (average mapping rate: 88.58%) compared to AM-CLR (average mapping rate: 86.15%) and AM-ONT (average mapping rate: 81.17%) (Supplementary Table S5). Third, the LAI value of the AM-T2T assembly was 22.67, meeting the gold standard for genome assemblies [37]. Fourth, the calculated QV of the AM-T2T assembly was 57.51, indicating a base call accuracy higher than 99.999% [35]. Fifth, the Hi-C heatmap demonstrated a high degree of consistency across all pseudochromosomes, confirming the precision in sequencing, ordering, and orientation of contigs (Fig. 2A). The GCI score of the AM-T2T genome was 36.23 (Table 1), significantly surpassing the GCI score of the chicken complete genome (29.37) [38]. Using the seven-base telomeric repeat as a sequence query, we identified all the 16 telomeres for the genome (Fig. 2B). Finally, comparative analysis between the AM-T2T genome and two publicly available *A. membranaceus* genomes (AM-ONT and AM-CLR) identified 158.58 Mb of previously unassembled regions (PURs; Supplementary Table S6). Taken together, these comprehensive validation results collectively demonstrate the exceptional quality and reliability of the AM-T2T genome assembly.

Genome annotation

Repetitive sequences in the AM-T2T genome were annotated using a combination of *de novo* and homology-based approaches, identifying 890.27 Mb of repeats, comprising 64.22% of the genome (Supplementary Table S7). This repetitive fraction was slightly smaller than those observed in the AM-CLR and AM-ONT (Table 1). The predominant repeat classes in AM-T2T were LTR retrotransposons and DNA transposons, which constituted 55.60% and 5.54% of the genome, respectively (Supplementary Table S8). Notably, 123.25 Mb (77.72%) of the PURs consisted of repetitive sequences. A total of 16,417 noncoding RNAs were identified, including 109 miRNAs, 1,544 tRNAs, 4,690 snRNAs, and 10,074 rRNAs (Supplementary Table S9).

The genome harbored 32,600 coding genes with an average coding sequence (CDS) length of 1,169.09 bp (Fig. 3; Table 1; Supplementary Table S10). BUSCO analysis indicated that 99.07% of core conserved plant orthologs were fully detected in the AM-T2T genome (Supplementary Table S11). This completeness level surpassed the metrics observed in the AM-CLR (96.59%) and AM-ONT (97.27%) assemblies. The length distributions of messenger RNA, CDS, exons, and introns among related species supported the reliability of the annotation results (Supplementary Fig. S2). Functional annotation assigned 98.34% of coding genes to public databases, validating prediction accuracy (Supplementary Table S12). Furthermore, 24,181 (74.17%) genes showed detectable transcriptional activity (FPKM $\geq$ 1) (Supplementary Fig. S3). Notably, 898 genes were annotated within PURs. GO enrichment analysis revealed that these PUR-associated genes were enriched in essential biological processes, such as “zinc ion binding”, “nucleic acid binding”, “ADP binding”, and more (Supplementary Fig. S4). Among these, 452 PUR genes showed expression (FPKM $\geq$ 1) in at least one sample (Supplementary Table S13). Genome-wide prediction identified

2,187 TFs across 58 types, exceeding the count in the AM-CLR genome (2,048) [72] (Supplementary Fig. S5). Significantly, four CYP450 genes (*CYP71B38*, *KAO2*, *CYP71A13*, and *CYP86A1*) localized within the PURs. These findings affirmed the completeness and accuracy of gene prediction in the AM-T2T assembly.

The characteristics of centromeric regions

The centromeric region of the genome presents a notable assembly challenge due to its high content of repetitive sequences [73]. So far, the centromeric sequence of the *A. membranaceus* genome has not been fully characterized, and our new T2T genome allows deeper exploration of the repeats in these regions. In the AM-T2T genome, centromeric sequences of all eight pseudochromosomes were assembled, with an average length of 2,949,226 bp (Table 2). The longest centromeric region, located on pseudochromosome 1, spanned 6,931,092 bp, while the shortest, on pseudochromosome 6, measured 351,503 bp. On average, centromere sequences contained 88.63% repeat sequences, with the primary transposable elements (TEs) being DNA transposons and LTRs. Notably, TE distribution within centromeric regions varied across chromosomes: LTRs predominated in pseudochromosome 1, whereas DNA transposons were prevalent in pseudochromosomes 3, 4, 5, and 6. In addition, the average content of tandem repeats is 54.49%, which is much higher than that in the whole genome (8.43%). These results are consistent with previous reports highlighting centromeric enrichment of retrotransposons and tandem repeats [74]. Within the chromosome centromeres of AM-T2T, a total of 169 genes were identified. Function enrichment analysis showed that these genes were significantly enrichment in multiple GO terms, such as “nucleic acid binding”, “RNA-DNA hybrid ribonuclease activity”, and “chitinase activity”, suggesting their

potential functions in the segregation of homologous chromosomes (Supplementary Fig. S6). Among these centromeric genes, 99 (58.58%) were expressed with an FPKM value greater than 1, a proportion slightly lower than that of all annotated genes (74.17%).

The identification of CYP450s and UGTs

A total of 248 and 236 CYP450 genes were identified in the AM-T2T and AMM genomes, respectively (Fig. 4A). Transcriptomic analysis revealed that 176 (70.97%) of these genes in AM-T2T were transcriptionally active (FPKM ≥ 1) in root, stem, or leaf tissues (Fig. 4B). Further, 32 CYP450 genes exhibited high expression (FPKM ≥ 10) specifically in roots and were identified as DEGs in both root vs. stem and root vs. leaf comparisons. Six of these 32 DEGs belonged to the CYP71 subfamily. Notably, two CYP71 genes (*CYP71B38* and *CYP71A13*) were localized to the PUR region with *CYP71B38* in centromeric regions. Previous studies suggested that CYP71 subfamily genes in *Panax ginseng* participate in the biosynthesis of secondary metabolites, aldehydes, and flavonoids [75]. Moreover, *CYP71D756* may participate in the biosynthesis of astragaloside IV in *Astragalus* genus plants [16].

For UGT genes, 163 and 149 members were detected in the AM-T2T and AMM genomes, respectively (Fig. 4C). Phylogenetic analysis revealed a significant contraction of the *UGT76* subfamily in both AM (2 genes) and AMM (2 genes) relative to *A. thaliana* (22 genes; Fig. 4C). By contrast, the *UGT73* subfamily showed significant expansion in AM (21 genes) and AMM (15 genes) compared to *A. thaliana* (9 genes). Moreover, 124 UGT genes (76.07%) in AM-T2T showed expression (FPKM ≥ 1) in root, stem, or leaf tissues (Fig. 4D). This expression ratio (76.07%) was higher than that of CYP450 genes (70.97%) and exceeded the genome-wide expression rate

(74.17%). Furthermore, the average expression level of UGT genes was highest in root tissues compared to stem and leaf tissues, suggesting a potentially critical functional role for UGT genes in *A. membranaceus* development, with particularly prominent activity in roots.

Unique genes and conserved genes

To investigate unique genes and conserved genes between the AM-T2T genome and other embryophyta, we selected nine species for gene family construction and phylogenomic tree inference. The statistical analysis of gene family identification results showed that a total of 28,023 gene families were identified, including 9,991 shared across all nine species. The AM-T2T genome harbored 169 unique gene families comprising 2,267 genes (Fig. 5A; Supplementary Table S14). Among these unique genes, 2,045 (90.21%) genes were supported by functional annotation, and 1,128 (49.76%) showed detectable expression (FPKM ≥ 1) in at least one sample (Supplementary Table S15). Expressed unique genes were significantly enriched in 34 GO terms, such as "nucleic acid binding", "DNA binding", "RNA−DNA hybrid ribonuclease activity", "zinc ion binding", "translation", among others (Supplementary Fig. S7). Notably, 47 TFs were identified among these expressed unique genes, implying a potential role in transcriptional regulation influencing the physiological traits of *A. membranaceus*. Future studies of these unique genes might dissect the regulatory genes and transcriptional networks underlying saponin/flavonoid biosynthesis via targeted mechanistic approaches.

A phylogenomic tree constructed from 1,153 single-copy genes showed that *A. membranaceus*, *A. mongholicus*, *C. arietinum*, and *M. truncatula* clustered within a major branch (Supplementary Fig. S8). Among the identified 19,271 gene families in the AM-T2T assembly, 14,674 (76.14%)

resided in this clade, encompassing 20,652 genes (Fig. 5B). These genes exhibited enrichment in 44 GO terms, with "ATP binding" (GO:0005524) being the most significant (Supplementary Fig. S9).

Whole genome comparative analysis between AM-T2T and AMM

Genome-wide comparative analysis of the AM-T2T and AMM genomes revealed that AM-T2T has a haploid pseudochromosome 8 (Fig. 6A), supported by the following lines of evidence: (i) Strong collinearity of pseudochromosome 8 across the AM-T2T, AM-CLR, and AM-ONT genomes indicates a low likelihood of assembly artifacts for this chromosome (Supplementary Fig. S10; Supplementary Fig. S11). (ii) Hi-C signal confirms the accuracy of the pseudochromosome 8 assembly (Fig. 2A). (iii) Comprehensive coverage of this region by both HiFi and ONT reads was observed (Fig. 6B). Additionally, this region has not been classified as low-confidence by CRAQ. The fusion region on pseudochromosome 8 was Chr08:91633450–91664996, which is composed of 91.51% repetitive sequences. Such a high proportion of repetitive sequences presents significant challenges for genome assembly. Future studies should explore the underlying mechanisms of these fusion events, their evolutionary timing, and their impacts on phenotypic traits, which represent important avenues for further investigation.

High-quality genome assemblies enabled comprehensive SV analysis. A total of 95,458 SVs were identified, including 40,764 insertions and 54,694 deletions (Fig. 6C). Among these, 42,397 (44.41%) resided within potential expression regulatory domains or CDSs of reference genes, herein defined as "SV-genes". Functional enrichment analysis revealed that these SV-genes were significantly associated with the following top ten pathways: "Endocytosis", "Tyrosine

metabolism", "Phenylalanine metabolism", "ATP-dependent chromatin remodeling", "Butanoate metabolism", "Spliceosome", "Tropane, piperidine and pyridine alkaloid biosynthesis", "Selenocompound metabolism", "Isoquinoline alkaloid biosynthesis", and "Glutathione metabolism" (Fig. 6D). The tyrosine metabolism pathway serves as a pivotal starting point for the biosynthesis of structurally diverse natural products in plants, while phenylalanine metabolism in soybean has been linked to phenylalanine ammonia-lyase, an enzyme critical for plant development and environmental stress responses [76, 77]. Notably, the SV-genes harbored 97 CYP450s and 51 UGTs. Collectively, these genomic variations provide a comprehensive resource for future fundamental and applied research on *A. membranaceus*.

Conclusions

This study presents the first high-quality telomere-to-telomere genome assembly of *A. membranaceus*, generated using PacBio HiFi reads, ONT sequencing, and Hi-C technologies. The assembled genome spans 1.39 Gb, encompassing 16 telomeres and 8 centromeres distributed across 8 chromosomes. The high quality of the assembly was verified by a 100% mapping rate of PacBio HiFi reads, 99.63% BUSCO completeness, higher RNA-seq mapping rates, a LAI of 22.67, a QV of 57.51, and a GCI score of 36.23. Genome annotation revealed 64.22% repetitive sequences, 32,600 protein-coding genes, 248 CYP450s, 163 UGTs, and 169 centromeric genes. Additionally, 158.58 Mb of PURs, 2,267 unique genes, and 20,652 conserved genes were identified. Genome-wide comparative analysis of the AM-T2T and AMM genomes revealed that AM-T2T has a haploid pseudochromosome 8. This study demonstrates the utility of advanced sequencing

technologies in resolving complex genomic regions and provides a more accurate foundation for genetic research of *A. membranaceus*.

Abbreviations

BLAST: Basic Local Alignment Search Tool; BUSCO: Benchmarking Universal Single-Copy Orthologs; CLR: continuous long reads; CRAQ: Clipping information for Revealing Assembly Quality; CYP450s: cytochrome P450 monooxygenases; FPKM: fragments per kilobase of transcript per million mapped reads; Gb: gigabase pairs; GCI: Genome Continuity Inspector; GO: Gene Ontology; Hi-C: High-Throughput Chromosome Conformation Capture; HiFi: High-Fidelity; IGV: Integrative Genomics Viewer; LTR: long terminal repeat; Mb: megabase pairs; miRNAs: microRNAs; NCBI: National Center for Biotechnology Information; ncRNA: non-coding RNA; NR: NCBI's nonredundant database; PASA: Program to Assemble Spliced Alignments; PUR: previously unassembled region; QV: quality value; RNA-seq: RNA sequencing; rRNAs: ribosomal RNAs ; snRNAs: small nuclear RNAs; T2T: telomere-to-telomere; tRNAs: Transfer RNAs; UGTs: uridine diphosphate glycosyltransferases

Additional Files

Supplementary Table 1. Summary of newly generated whole genome sequencing data used in this study.

Supplementary Table 2. The statistics of the contig assembly.

Supplementary Table 3. The statistics of the anchored chromosome length.

Supplementary Table 4. The low confidence region within the AM-T2T assembly.

Supplementary Table 5. Summary of the RNA-seq reads genome mapping rate.

Supplementary Table 6. The identification of PUR region in AM-T2T assembly.

Supplementary Table 7. General statistics of repeats in AM-T2T assembly.

Supplementary Table 8. The summary of interspersed repeat contents in AM-T2T assembly.

Supplementary Table 9. Annotation of ncRNA in the AM-T2T assembly.

Supplementary Table 10. The length statistics of genes in AM-T2T assembly.

Supplementary Table 11. BUSCOs analysis of AM-T2T gene set completeness.

Supplementary Table 12. Number of functional annotations for predicted genes in AM-T2T assembly.

Supplementary Table 13. The gene list in PUR region of AM-T2T assembly.

Supplementary Table 14. Gene families in AM-T2T and other species.

Supplementary Table 15. The list of AM-T2T unique genes.

Author Contributions

Hongling Tian designed this study; Huazhi Wang collected the samples and performed the experiments; Aohui Li and Shuyu Zhong performed the data analysis; Huibin Qin wrote the first draft of the manuscript. All other authors proofread and revised the manuscript. All authors read and approved the final manuscript.

Funding

This research was funded by the Fundamental Research Program of Shanxi Province (No. 202103021224158), the National Natural Science Foundation of China (No. 31601457), Chinese

Materia Medica, China Agriculture Research System (CARS-21), and the Hengshan Astragalus Research Institute's Local Partnership Project (XDHZHQY2022-01).

Data Availability

The raw sequencing data that support the findings of this study have been deposited into NCBI with accession number SRA: SRR35052312 and EBI with accession number ENA: ERR15401760. All additional supporting data are available in the *GigaScience* repository, GigaDB [78].

Competing Interests

The authors declare that they have no competing interests.

Acknowledgements

We thank every project that provides funding and material support for the study. We also thank each author for their ideas and skills in study design, experimentation, data collection, data analysis, and manuscript writing.

References

1. Fu, J., et al., *Review of the botanical characteristics, phytochemistry, and pharmacology of Astragalus membranaceus (Huangqi).* Phytotherapy research : PTR, 2014. **28**(9): p. 1275-1283.
2. Auyeung, K.K., Q.-B. Han, and J.K. Ko, *Astragalus membranaceus: A Review of its Protection Against Inflammation and Gastrointestinal Cancers.* The American journal of Chinese medicine, 2016. **44**(1): p. 1-22.
3. Li, C.-X., et al., *Astragalus polysaccharide: a review of its immunomodulatory effect.* Archives of pharmacal research, 2022. **45**(6): p. 367-389.
4. Chen, J., et al., *Global transcriptome analysis profiles metabolic pathways in traditional herb Astragalus membranaceus Bge. var. mongolicus (Bge.) Hsiao.* BMC genomics, 2015. **16 Suppl 7**: p. S15.
5. Kim, Y.B., et al. *Accumulation of astragalosides and related gene expression in different organs of Astragalus membranaceus Bge. var mongholicus (Bge.).* Molecules (Basel, Switzerland), 2014. **19**, 10922-10935 DOI: 10.3390/molecules190810922.
6. Kim, Y.B., et al., *Accumulation of flavonoids and related gene expressions in different organs of Astragalus membranaceus Bge.* Applied biochemistry and biotechnology, 2014. **173**(8): p. 2076-2085.

7. Yu, M., et al., *Genome-wide DNA methylation analysis of Astragalus on the intervention of ID2 promoter via PI3K/Akt signaling pathway in peritoneal fibrosis.* Sci Rep, 2025. **15**(1): p. 15786.
8. Xu, W.-x., et al., *Eight new triterpenoid saponins from the leaves of Astragalus membranaceus (Fisch.) Bunge and their neuroprotective effects.* Fitoterapia, 2025. **183**: p. 106559.
9. Xiao, S., et al., *Astragenol alleviates neuroinflammation and improves Parkinson's symptoms through amino acid metabolism pathway and inhibition of ferroptosis.* J Ethnopharmacol, 2025. **348**: p. 119896.
10. Zhang, K., et al., *Assembly and comparative analysis of the first complete mitochondrial genome of Astragalus membranaceus (Fisch.) Bunge: an invaluable traditional Chinese medicine.* BMC Plant Biology, 2024. **24**(1): p. 1055.
11. Li, J., et al., *Long read reference genome-free reconstruction of a full-length transcriptome from Astragalus membranaceus reveals transcript variants involved in bioactive compound biosynthesis.* Cell Discovery, 2017. **3**(1): p. 17031.
12. Wang, J., et al., *Characterization of the WRKY family transcription factors in Astragalus membranaceus and their expression under drought stress.* BMC Plant Biol, 2025. **25**(1): p. 593.
13. Qi, L., et al., *The AmMYB35-AmFLS module mediates the accumulation of flavonol induced by drought stress in Astragalus membranaceus.* Food Bioscience, 2025. **68**: p. 106541.
14. Hwang, C., et al., *Efficient hairy root induction system of Astragalus membranaceus and significant enhancement of astragalosides via overexpressing AmUGT15.* Plant Cell Rep, 2024. **43**(12): p. 285.
15. Fan, H., et al., *Chromosome-scale genome assembly of Astragalus membranaceus using PacBio and Hi-C technologies.* Scientific Data, 2024. **11**(1): p. 1071.
16. Xu, B., et al., *Total biosynthesis of the medicinal triterpenoid saponin astragalosides.* Nature Plants, 2024.
17. Chen, Y., et al., *A reference-grade genome assembly for Astragalus mongholicus and insights into the biosynthesis and high accumulation of triterpenoids and flavonoids in its roots.* Plant Communications, 2022. **4**.
18. Shang, L., et al., *A complete assembly of the rice Nipponbare reference genome.* Molecular plant, 2023. **16**(8): p. 1232-1236.
19. Chen, J., et al., *A complete telomere-to-telomere assembly of the maize genome.* Nature genetics, 2023. **55**(7): p. 1221-1231.
20. Li, M., et al. *Telomere-to-telomere genome assembly of sorghum*. Scientific data, 2024. **11**, 835 DOI: 10.1038/s41597-024-03664-8.
21. Li, H. and R. Durbin, *Genome assembly in the telomere-to-telomere era.* Nature Reviews Genetics, 2024. **25**(9): p. 658-670.
22. Garg, V., et al., *Unlocking plant genetics with telomere-to-telomere genome assemblies.* Nature Genetics, 2024.
23. De Coster, W., et al., *NanoPack: visualizing and processing long-read sequencing data.* Bioinformatics, 2018. **34**: p. 2666 - 2669.
24. Cheng, H., et al., *Haplotype-resolved de novo assembly using phased assembly graphs with hifiasm.* Nature Methods, 2021. **18**(2): p. 170-175.
25. Kolmogorov, M., et al., *Assembly of long, error-prone reads using repeat graphs.* Nature Biotechnology, 2019. **37**(5): p. 540-546.
26. Hu, J., et al., *NextDenovo: an efficient error correction and accurate assembly tool for noisy long reads.* Genome Biology, 2024. **25**(1): p. 107.
27. Ruan, J. and H. Li, *Fast and accurate long-read assembly with wtdbg2.* Nature Methods, 2020. **17**(Suppl 6): p. 1-4.
28. Zeng, X., et al., *Chromosome-level scaffolding of haplotype-resolved assemblies using Hi-C data without*

reference genomes. Nature plants, 2024. **10**(8): p. 1184-1200.
29. Durand, N.C., et al., *Juicebox Provides a Visualization System for Hi-C Contact Maps with Unlimited Zoom.* Cell systems, 2016. **3**(1): p. 99-101.
30. Lin, Y., et al., *quarTeT: a telomere-to-telomere toolkit for gap-free genome assembly and centromeric repeat identification.* Horticulture Research, 2023.
31. Xu, G.-C., et al., *LR_Gapcloser: a tiling path-based gap closer that uses long reads to complete genome assembly.* GigaScience, 2018. **8**.
32. Jain, C., et al., *Long-read mapping to repetitive reference sequences using Winnowmap2.* Nature methods, 2022. **19**(6): p. 705-710.
33. Vaser, R., et al., *Fast and accurate de novo genome assembly from long uncorrected reads.* Genome research, 2017. **27 5**: p. 737-746.
34. Seppey, M., M. Manni, and E.M. Zdobnov, *BUSCO: Assessing Genome Assembly and Annotation Completeness.* Methods in molecular biology (Clifton, N.J.), 2019. **1962**: p. 227-245.
35. Rhie, A., et al., *Merqury: reference-free quality, completeness, and phasing assessment for genome assemblies.* Genome Biology, 2020. **21**.
36. Li, H., *Minimap2: pairwise alignment for nucleotide sequences.* Bioinformatics, 2018. **34**(18): p. 3094-3100.
37. Ou, S., J. Chen, and N. Jiang, *Assessing genome assembly quality using the LTR Assembly Index (LAI).* Nucleic acids research, 2018. **46**(21): p. e126.
38. Chen, Q., et al., *GCI: a continuity inspector for complete genome assembly.* Bioinformatics, 2024. **40**(11).
39. Li, K., et al., *Identification of errors in draft genome assemblies at single-nucleotide resolution for quality assessment and improvement.* Nature Communications, 2023. **14**(1): p. 6556.
40. Bergman, C.M. and H. Quesneville, *Discovering and detecting transposable elements in genome sequences.* Briefings in Bioinformatics, 2007. **8**(6): p. 382-392.
41. Xu, Z. and H. Wang, *LTR-FINDER: An efficient tool for the prediction of full-length LTR retrotransposons.* Nucleic acids research, 2007. **35**: p. W265-8.
42. Benson, G., *Tandem repeats finder: a program to analyze DNA sequences.* Nucleic acids research, 1999. **27 2**: p. 573-80.
43. Grabherr, M.G., et al., *Full-length transcriptome assembly from RNA-Seq data without a reference genome.* Nature biotechnology, 2011. **29 7**: p. 644-52.
44. Haas, B., *Improving the Arabidopsis genome annotation using maximal transcript alignment assemblies.* Nucleic Acids Research, 2003. **31**: p. 5654-5666.
45. Kim, D., B. Langmead, and S.L. Salzberg, *HISAT: a fast spliced aligner with low memory requirements.* Nature Methods, 2015. **12**(4): p. 357-360.
46. Kovaka, S., et al., *Transcriptome assembly from long-read RNA-seq alignments with StringTie2.* Genome Biology, 2019. **20**(1): p. 278.
47. Hou, X., et al., *A near-complete assembly of an Arabidopsis thaliana genome.* Molecular plant, 2022. **15**(8): p. 1247-1250.
48. Zhang, C., et al., *The T2T genome assembly of soybean cultivar ZH13 and its epigenetic landscapes.* Molecular plant, 2023. **16**(11): p. 1715-1718.
49. Jens, et al., *GeMoMa: Homology-Based Gene Prediction Utilizing Intron Position Conservation and RNA-seq Data.* Methods in Molecular Biology, 2019.
50. Haas, B.J., et al., *Automated eukaryotic gene structure annotation using EVidenceModeler and the Program to Assemble Spliced Alignments.* Genome Biology, 2008. **9**(1): p. R7.
51. Buchfink, B., C. Xie, and D.H. Huson, *Fast and sensitive protein alignment using DIAMOND.* Nature Methods, 2015. **12**(1): p. 59-60.

52. Bairoch, A. and R. Apweiler, *The SWISS-PROT protein sequence data bank and its supplement TrEMBL in 1999.* Nucleic Acids Research, 1999. **27**(1): p. 49-54.
53. Kanehisa, M. and S. Goto, *KEGG: Kyoto Encyclopedia of Genes and Genomes.* Nucleic Acids Research, 2000. **28**(1): p. 27-30.
54. Jones, P., et al., *InterProScan 5: genome-scale protein function classification.* Bioinformatics, 2014. **30**(9): p. 1236-1240.
55. Wu, Y.-M., et al. *Investigation of sex expression profiles and the cantharidin biosynthesis genes in two blister beetles*. PloS one, 2023. **18**, e0290245 DOI: 10.1371/journal.pone.0290245.
56. Langmead, B. and S.L. Salzberg, *Fast gapped-read alignment with Bowtie 2.* Nature Methods, 2012. **9**(4): p. 357-359.
57. Li, B. and C.N. Dewey, *RSEM: accurate transcript quantification from RNA-Seq data with or without a reference genome.* BMC Bioinformatics, 2011. **12**(1): p. 323.
58. Love, M.I., W. Huber, and S. Anders, *Moderated estimation of fold change and dispersion for RNA-seq data with DESeq2.* Genome biology, 2014. **15**(12): p. 550.
59. Li, D., et al., *A high-quality genome assembly of the eggplant provides insights into the molecular basis of disease resistance and chlorogenic acid synthesis.* Molecular ecology resources, 2021. **21**(4): p. 1274-1286.
60. Lowe, T.M. and S.R. Eddy, *tRNAscan-SE: a program for improved detection of transfer RNA genes in genomic sequence.* Nucleic acids research, 1997. **25**(5): p. 955-964.
61. Wang, H., et al. *A complete reference genome of broomcorn millet*. Scientific data, 2024. **11**, 657 DOI: 10.1038/s41597-024-03489-5.
62. Eddy, S.R., *Profile hidden Markov models.* Bioinformatics, 1998. **14**(9): p. 755-763.
63. Zhang, Y., et al., *P450Rdb: A manually curated database of reactions catalyzed by cytochrome P450 enzymes.* Journal of Advanced Research, 2024. **63**: p. 35-42.
64. Nakamura, T., et al., *Parallelization of MAFFT for large-scale multiple sequence alignments.* Bioinformatics (Oxford, England), 2018. **34**(14): p. 2490-2492.
65. Capella-Gutiérrez, S., J.M. Silla-Martínez, and T. Gabaldón, *trimAl: a tool for automated alignment trimming in large-scale phylogenetic analyses.* Bioinformatics (Oxford, England), 2009. **25**(15): p. 1972-1973.
66. Minh, B.Q., et al., *IQ-TREE 2: New Models and Efficient Methods for Phylogenetic Inference in the Genomic Era.* Molecular biology and evolution, 2020. **37**(5): p. 1530-1534.
67. Liu, Y., et al., *pUGTdb: A comprehensive database of plant UDP-dependent glycosyltransferases.* Molecular plant, 2023. **16**(4): p. 643-646.
68. Marçais, G., et al., *MUMmer4: A fast and versatile genome alignment system.* PLoS Computational Biology, 2018. **14**.
69. Xie, C., et al., *KOBAS 2.0: a web server for annotation and identification of enriched pathways and diseases.* Nucleic acids research, 2011. **39**(Web Server issue): p. W316-22.
70. quan, W.S., *Karyotype Analysis of Astragalus membranaceus.* Hubei Agricultural Sciences, 2006.
71. Hong, K., *karyotype diversity of six Astragalus species.* Guihaia, 2012. **32(5)**: p. 579–582.
72. Fan, H., et al. *Chromosome-scale genome assembly of Astragalus membranaceus using PacBio and Hi-C technologies*. Scientific data, 2024. **11**, 1071 DOI: 10.1038/s41597-024-03852-6.
73. Deng, Y., et al., *A telomere-to-telomere gap-free reference genome of watermelon and its mutation library provide important resources for gene discovery and breeding.* Molecular plant, 2022. **15**.
74. Liu, Y., et al., *Genome-wide mapping reveals R-loops associated with centromeric repeats in maize.* Genome research, 2021. **31**(8): p. 1409-1418.
75. Seitz, C., et al., *Cloning, functional identification and sequence analysis of flavonoid 3'-hydroxylase and flavonoid 3',5'-hydroxylase cDNAs reveals independent evolution of flavonoid 3',5'-hydroxylase in the*

Asteraceae family. Plant molecular biology, 2006. **61**(3): p. 365-381.

76. Rizwan, H.M., et al., *Characterization of phenylalanine ammonia-lyase genes in soybean: genomic insights and expression analysis under abiotic stress tolerance.* Plant Stress, 2025. **16**: p. 100896.

77. Xu, J.-J., et al., *General and specialized tyrosine metabolism pathways in plants.* aBIOTECH, 2020. **1**(2): p. 97-105.

78. Qin H, Li A, Zhong S, Wang H, Tian H. Supporting data for "The complete genome assembly of Astragalus membranaceus: enabling more accurate genetic research" GigaScience Database. 2025. https://doi.org/10.5524/102751

Figures and Legends

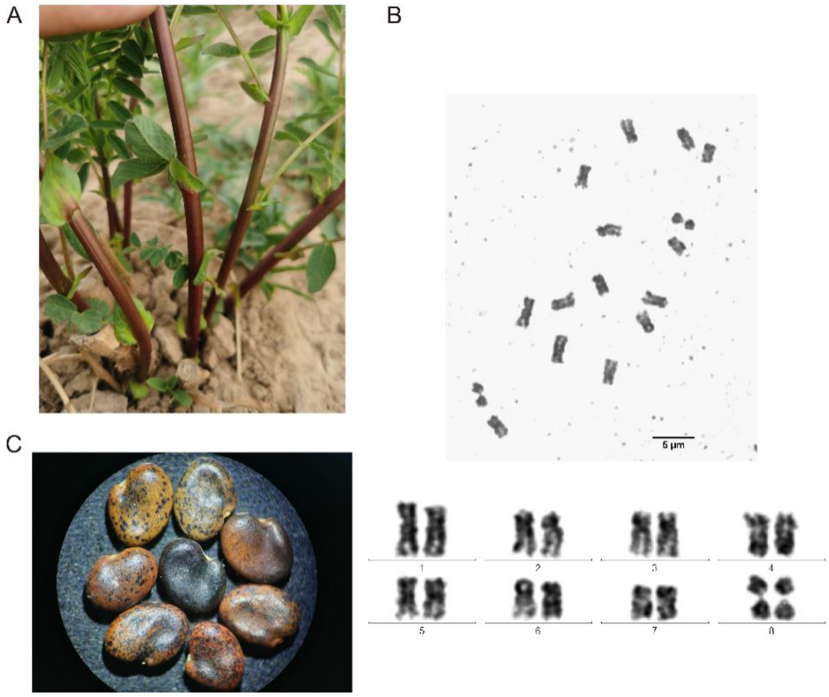

Fig. 1 | The morphology and karyotype of a *Astragalus membranaceus* (Fisch.) Bunge. A, The stems of *A. membranaceus*. **B,** The karyotype of *A. membranaceus* via karyotype analysis. **C,** The seeds of *A. membranaceus*.

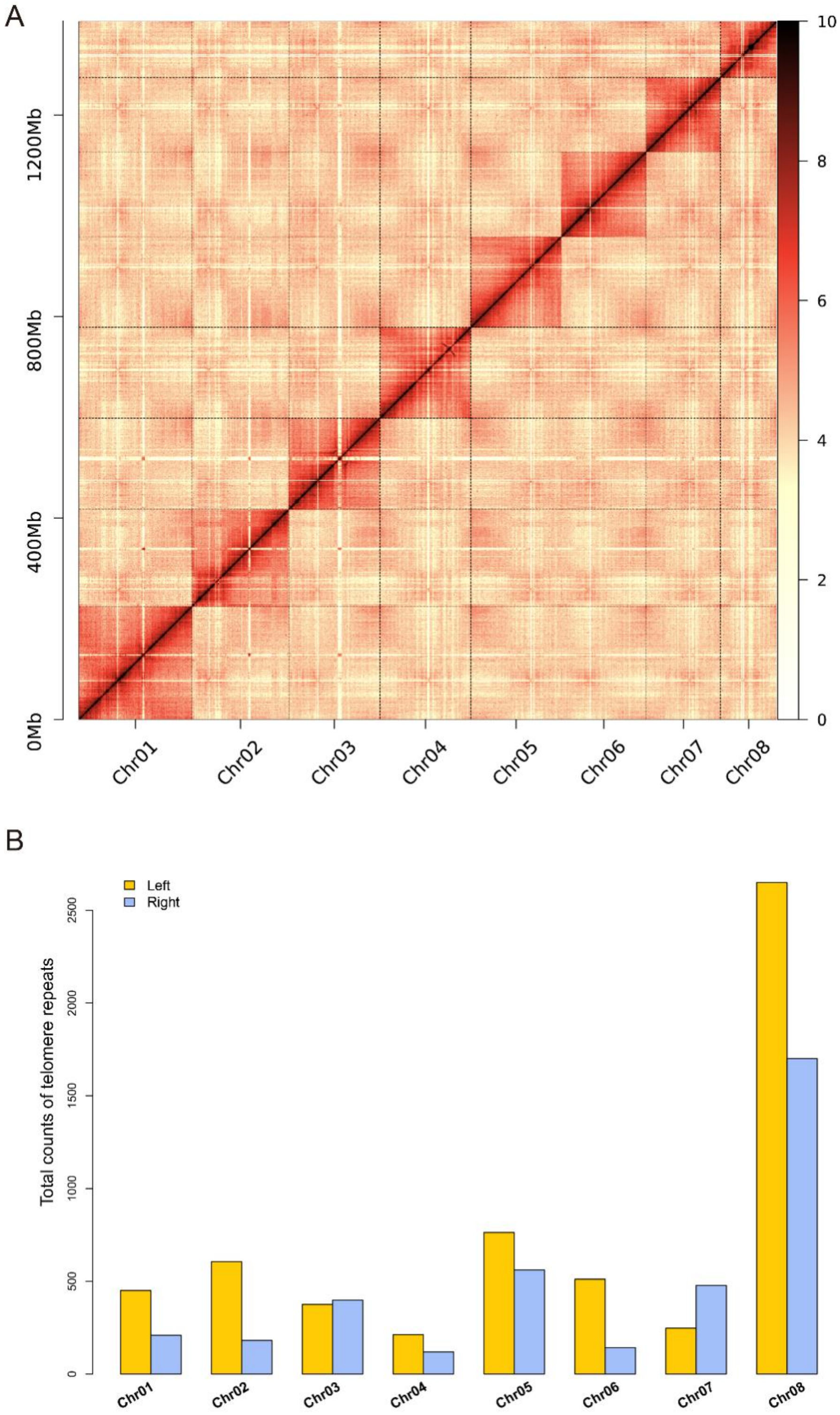

Fig. 2 | Genomic characteristics of AM-T2T assembly. A, Intensity signal heat map of the Hi-C chromosome interaction. The colour block illuminates the intensity of interaction from yellow (low) to red (high). **B**, The statistics of telomere repeats.

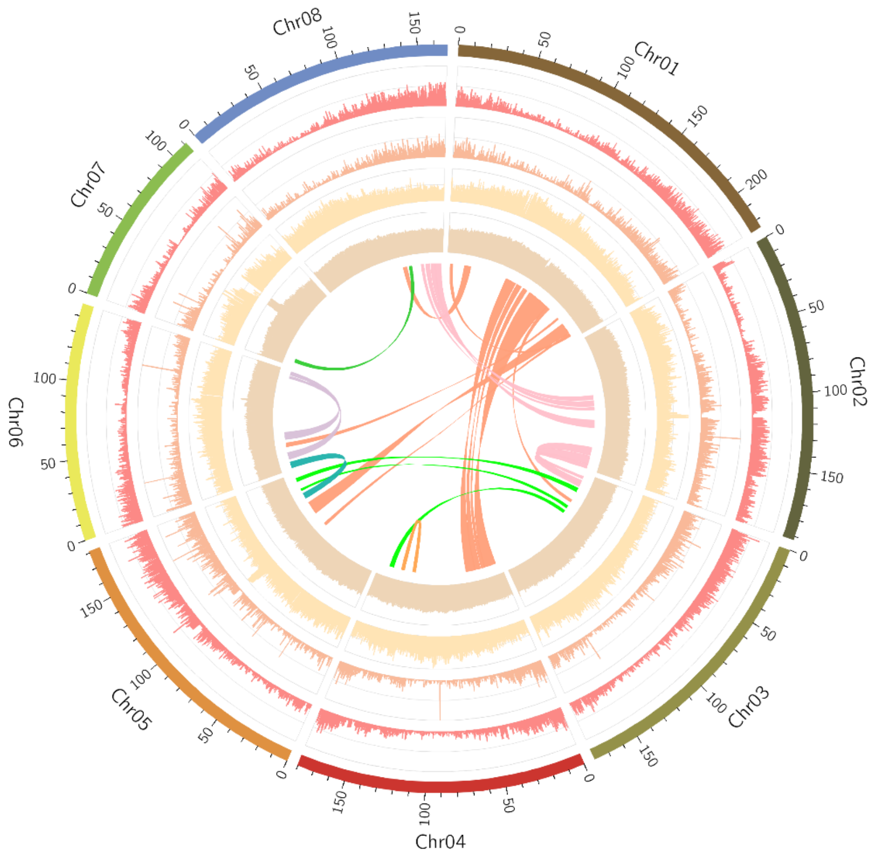

Fig. 3 | Circos plot of the assembled AM-T2T genome. Circular tracks from outside to inside indicate the pseudomolecules; gene density; gene length; repetitive density; GC content. The links in the center show syntenic region found in each chromosome.

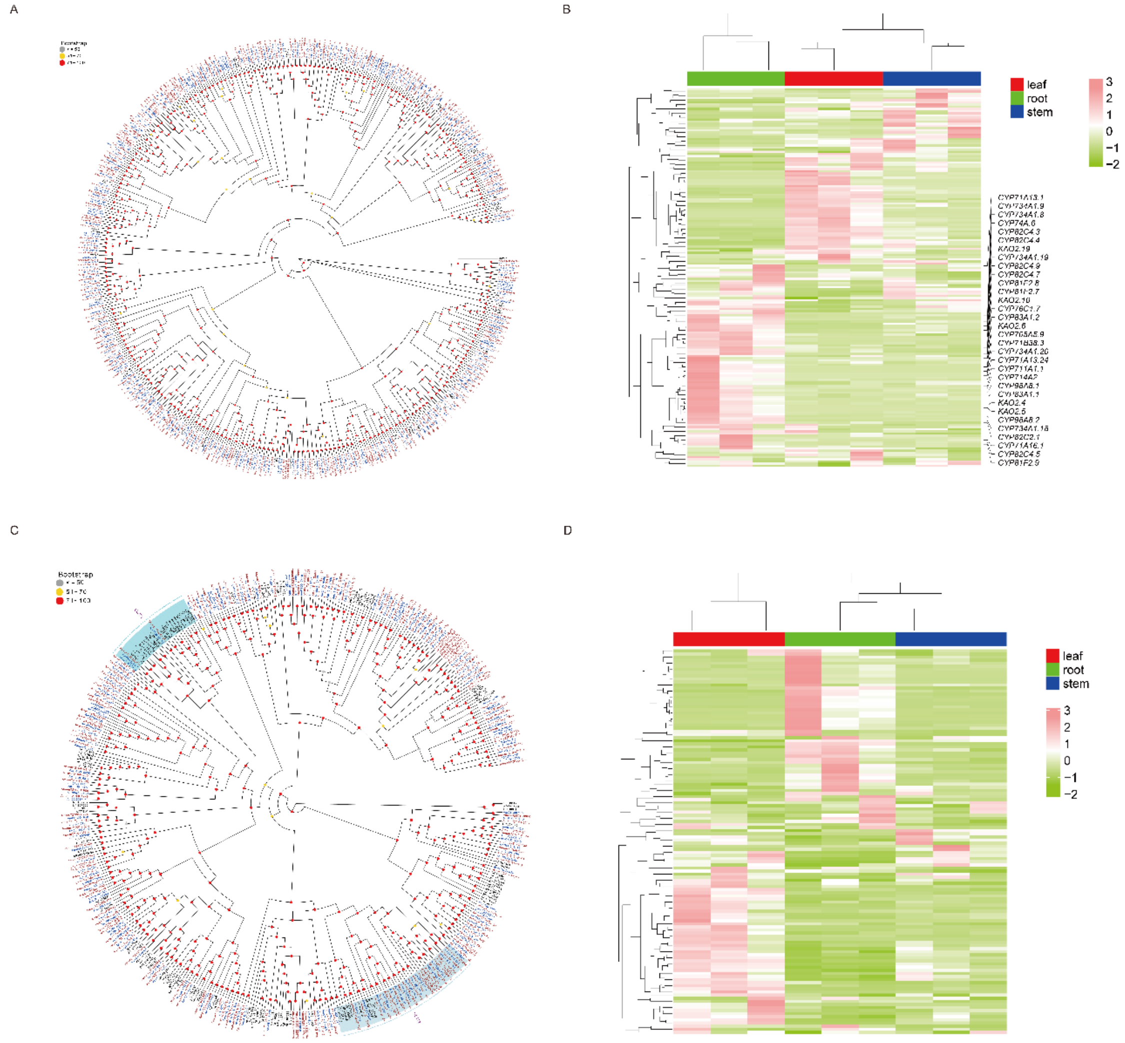

Fig. 4 | The identification of CYP450s and UGTs. A, The phylogenetic tree of CYP450s. B, The heatmap of CYP450s. C, The phylogenetic tree of UGTs. D, The heatmap of UGTs. Genes with IDs highlighted in brown represent those originating from AM-T2T; Genes with IDs highlighted in blue denote genes from AMM, and those in black denote genes from *A. thaliana*.

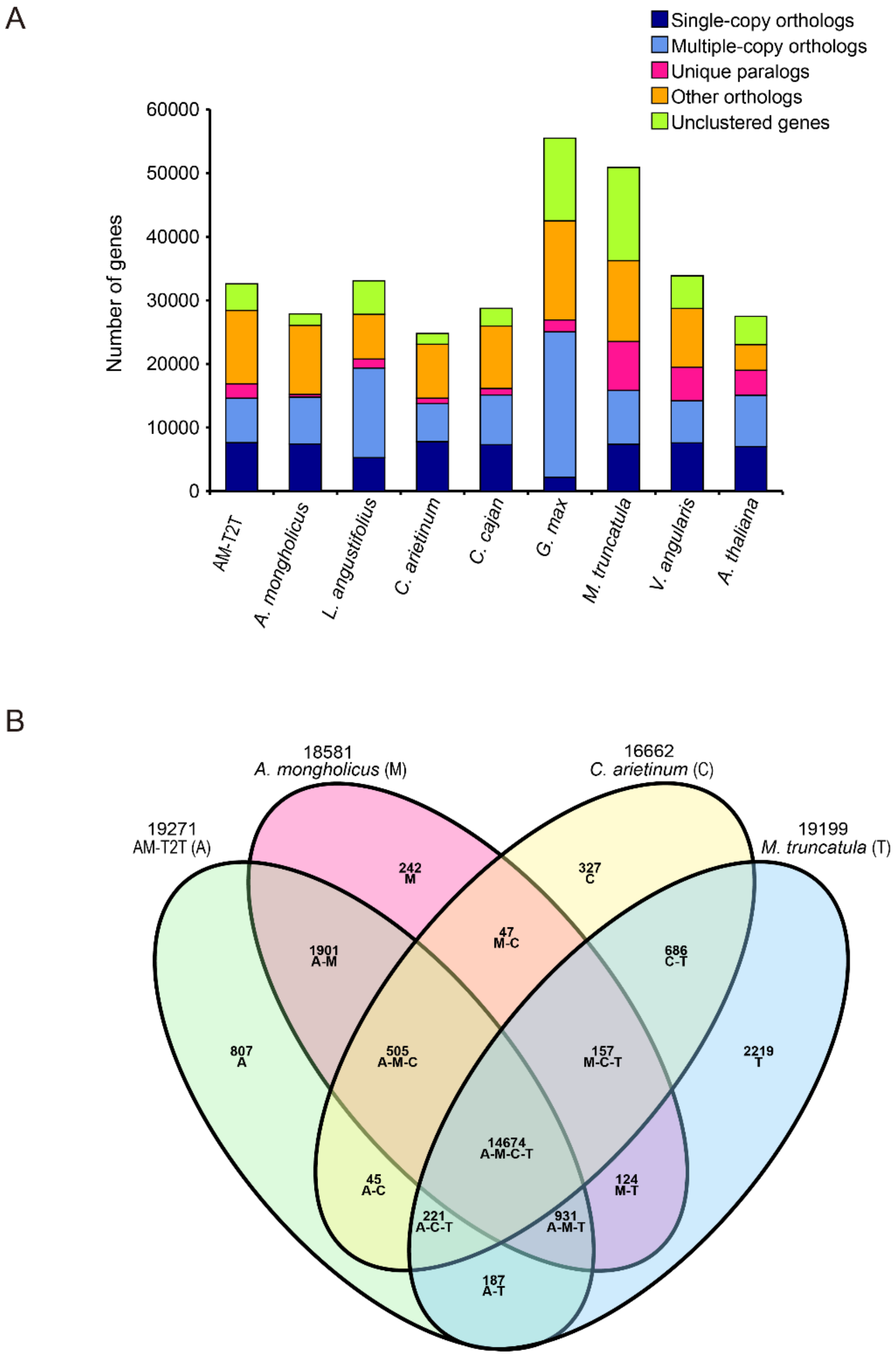

Fig. 5 | Evolution of AM-T2T genome. A, Number of orthologous genes in nine species. **B,** Venn diagram of orthologous gene families in four genomes. The numbers represent quantities of gene families.

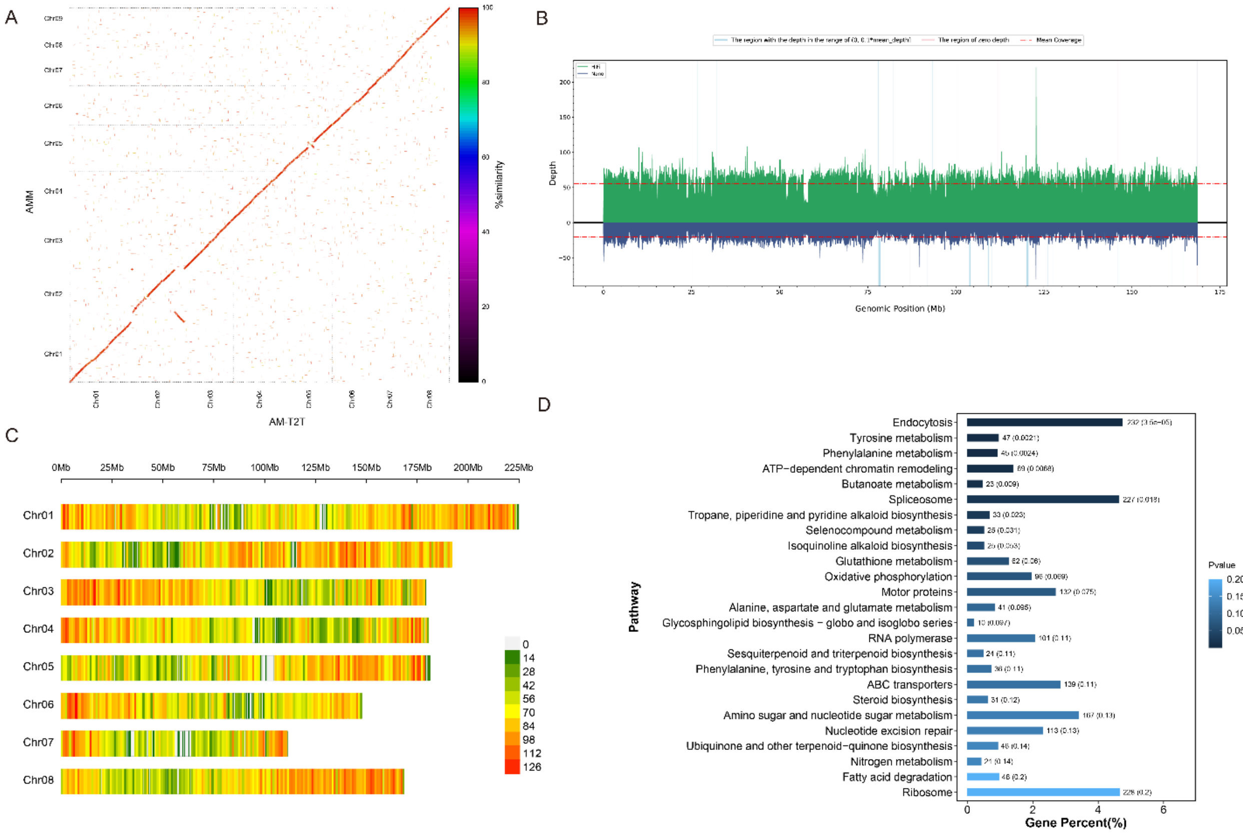

Fig. 6 | Whole genome comparative analysis of between AM-T2T and AMM. A, Comparison of sequence synteny between AM-T2T and AMM. **B,** The filtered depth of PacBio HiFi and ONT reads across pseudochromosome 8. **C,** The density plot of SVs between AM-T2T and AMM. **D,** KEGG enrichment analysis of the SV-genes.

Click here to access/download;Table;Table 1. Assembly statistics of AM-T2T genome assembly.xlsx

Table 1. Assembly statistics of *A. membranaceus* genome assembly.

Genomic feature	AM-T2T
Number of contigs (gaps)	8 (0)
Chromosome number	8
Assembly length (Mb)	1,386.22
Contig N50 (Mb)	180.45
Scaffold N50 (Mb)	180.45
HiFi reads mapping rate (%)	100.00
HiFi reads coverage (%)	99.97
Number of telomeres	16
Protein-coding genes number	32,600
Repeat content (%)	64.22
Genome BUSCOs (%)	99.63
GCI score	36.23
LTR assembly index	22.67
Quality value	57.51

**AM-ONT (Xu *et al.* , 2024)**	**AM-CLR (Fan *et al* ., 2024)**
1,060 (1,032)	1,773 (1,432)
9	9
1,439.71	1,431.18
2.82	1.67
184.69	184.46
-	-
-	-
1	6
38,398	29,914
68.20	67.98
93.37	97.27
-	-
-	16.22
-	48.58

Table2 Click here to access/download;Table;Table 2.xlsx

Table 2. The characteristic of centromeric regions of the AM-T2T assembly.

Chromosome	Start	End	GC content	Gene number
Chr01	123,245,059	130,176,150	37.0%	57
Chr02	30,456,263	31,781,639	39.7%	11
Chr03	124,400,000	125,180,000	39.3%	0
Chr04	93,780,950	96,286,216	38.7%	18
Chr05	54,322,074	55,622,641	39.3%	10
Chr06	86,261,760	86,613,262	39.2%	1
Chr07	43,829,593	47,989,591	39.8%	38
Chr08	51,840,000	58,080,000	40.1%	34

Repetitive sequence content (%)	Tandem repeats content (%)
84.92	34.07
84.75	51.33
97.94	85.02
92.76	57.10
84.19	49.08
96.62	77.58
84.12	43.91
83.78	37.84

DNA transposons content (%)	LTR content (%)
1.57	77.50
47.81	36.66
72.89	23.65
53.21	34.99
50.17	35.48
65.91	26.60
39.80	44.46
31.06	51.25

Figure1

Click here to access/download;Figure;Figure1.png

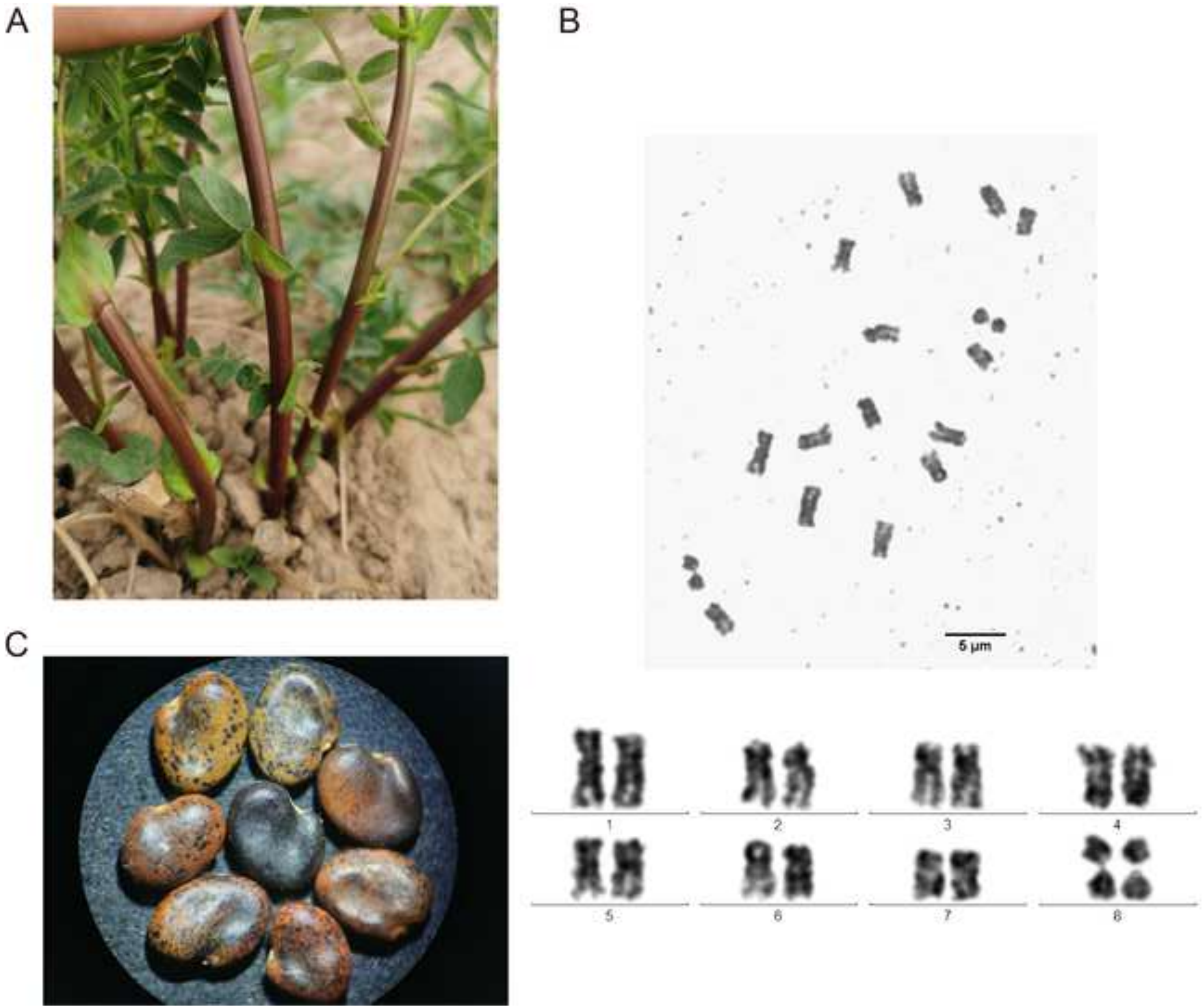

Figure2

Click here to access/download;Figure;Figure2.png

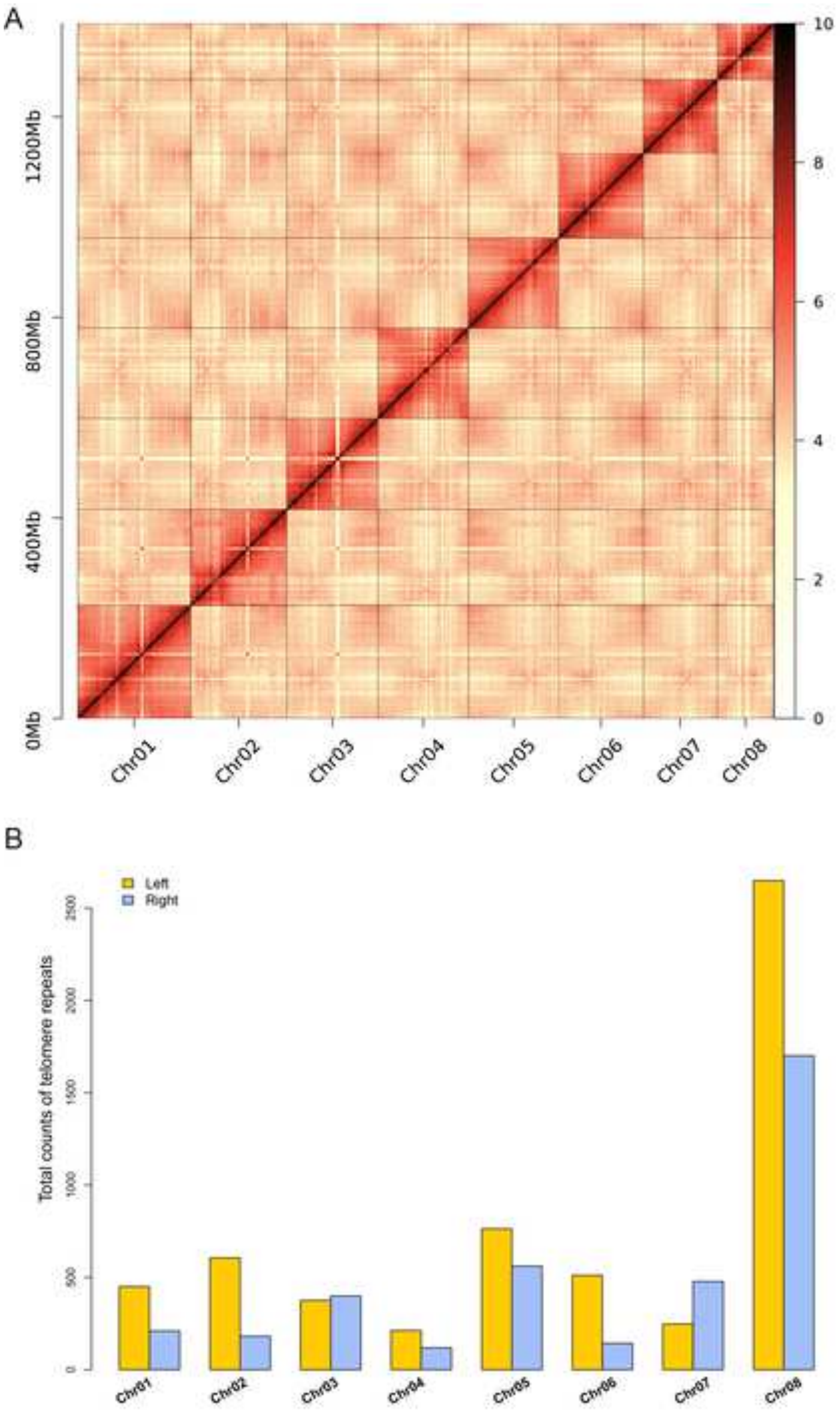

Figure3

Click here to access/download;Figure;Figure3.png

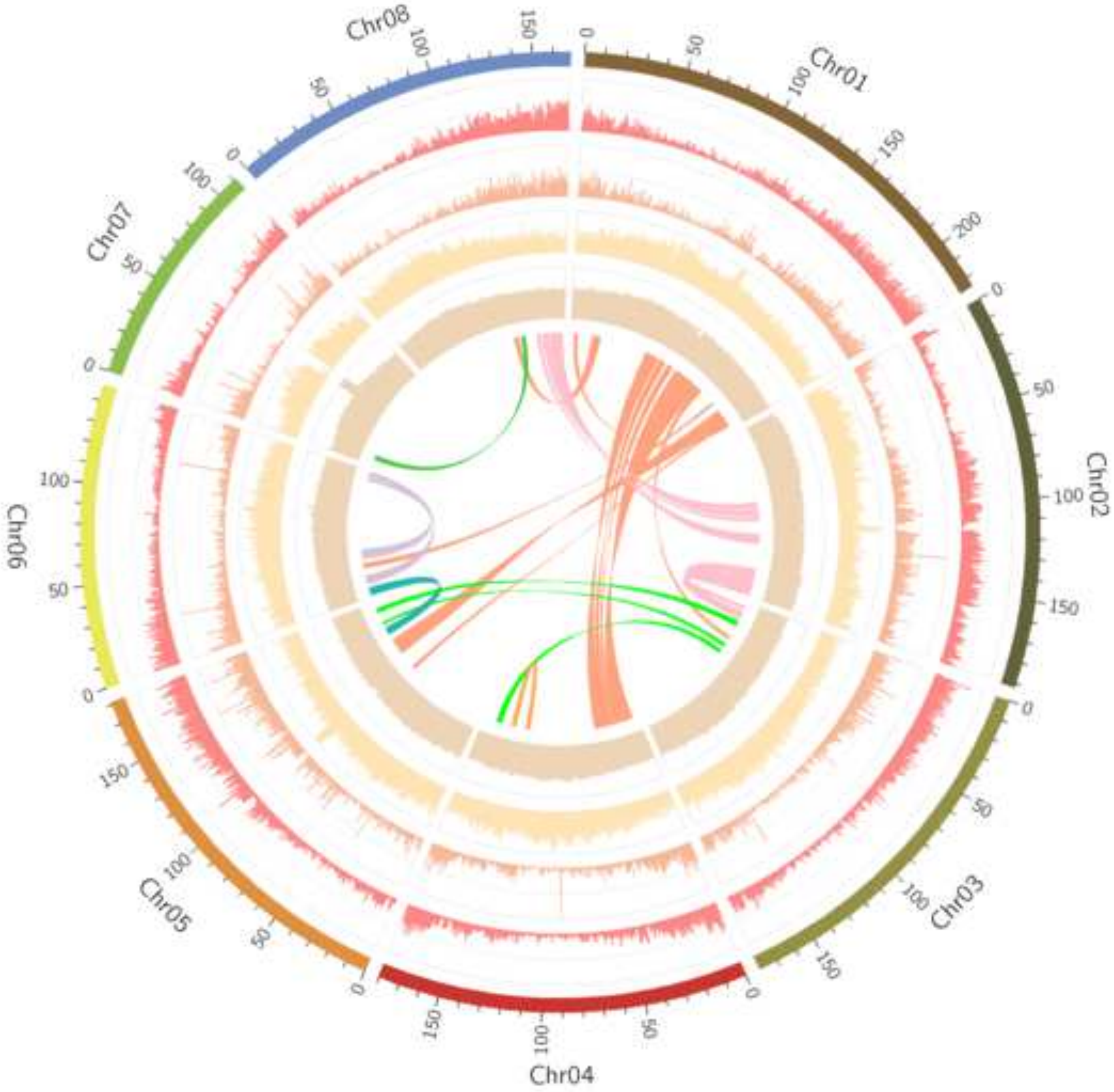

Figure4 Click here to access/download;Figure;Figure4.png

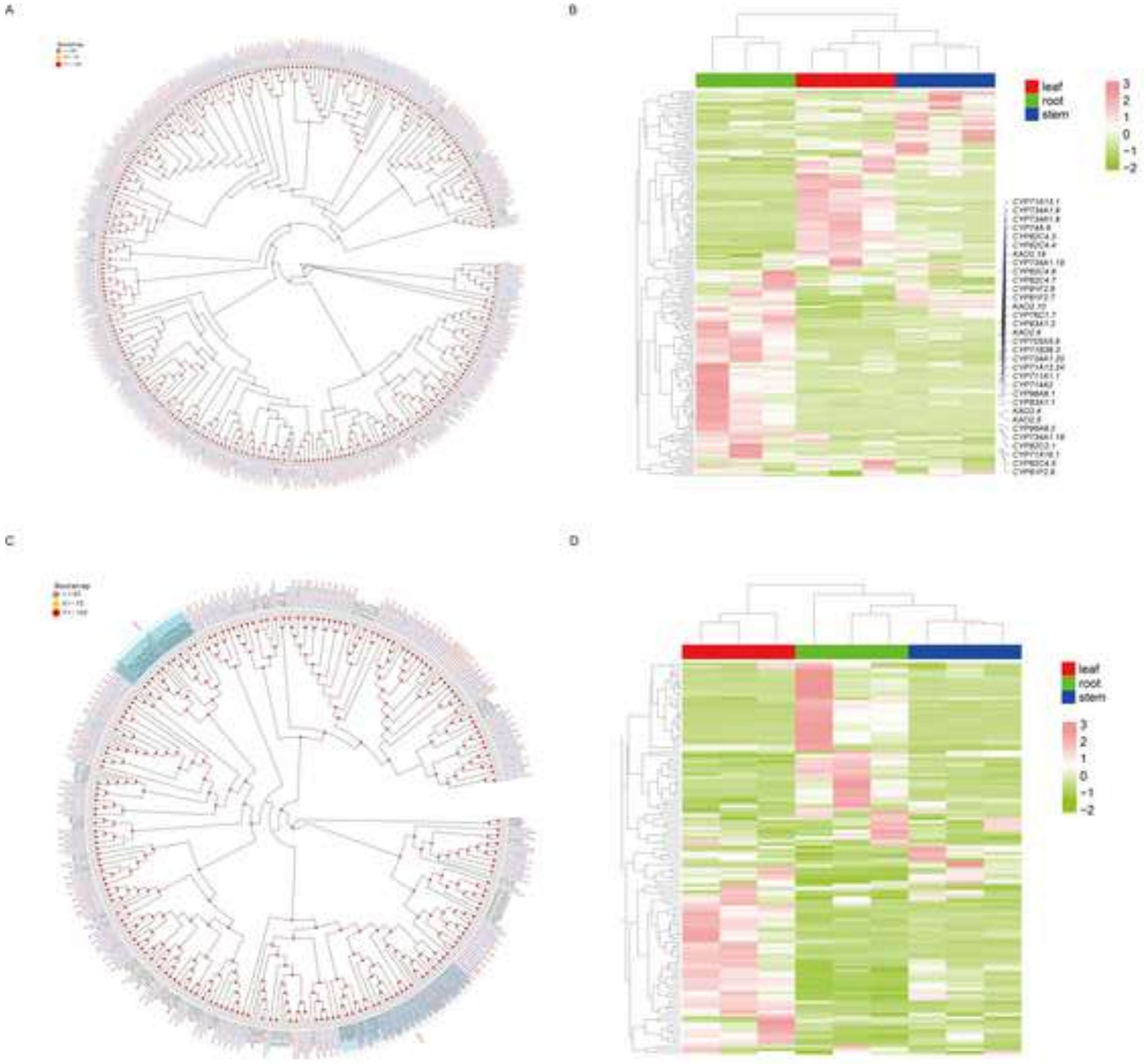

Figure5

Click here to access/download;Figure;Figure5.png

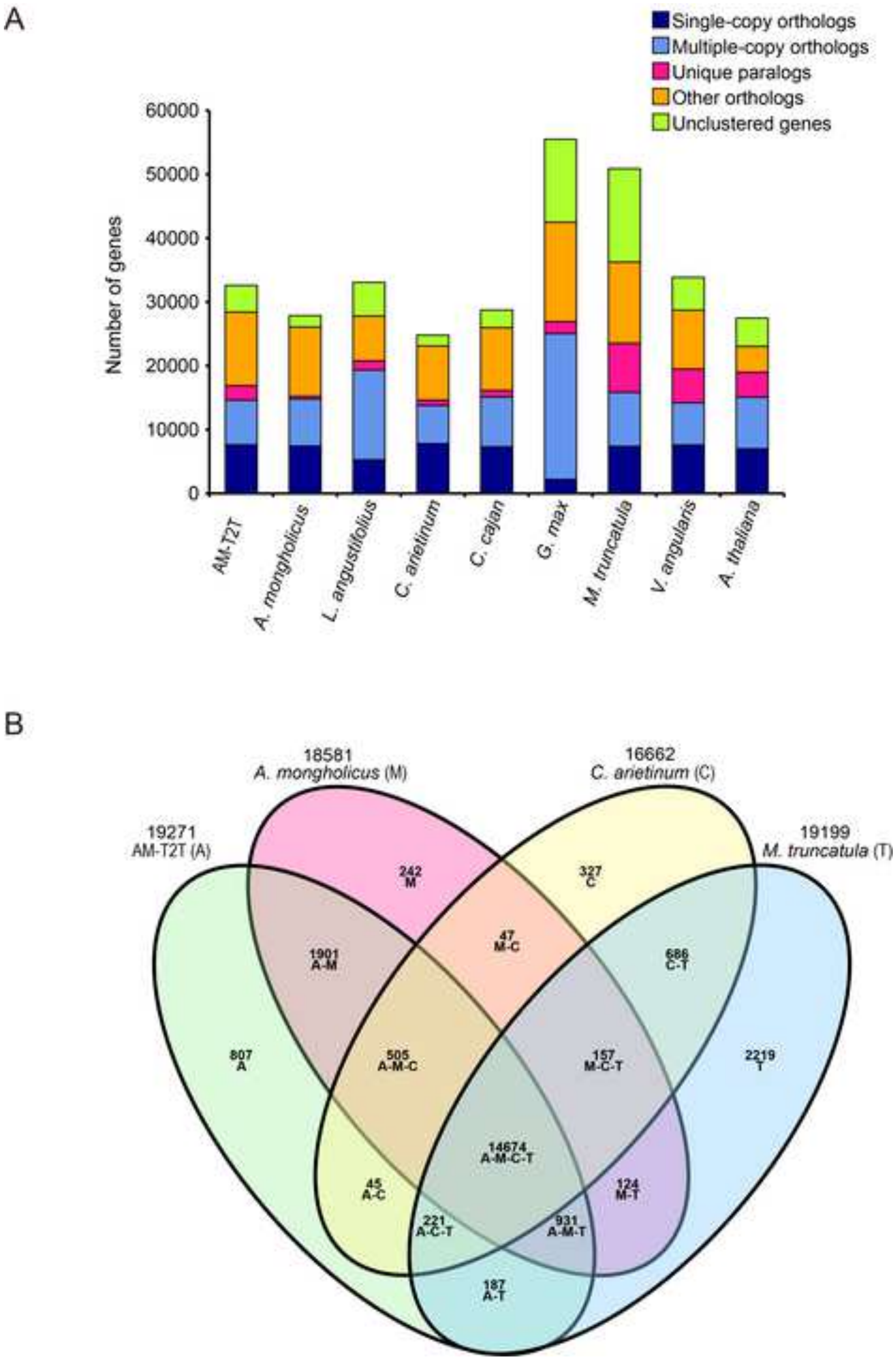

Figure6

Click here to access/download;Figure;Figure6.png

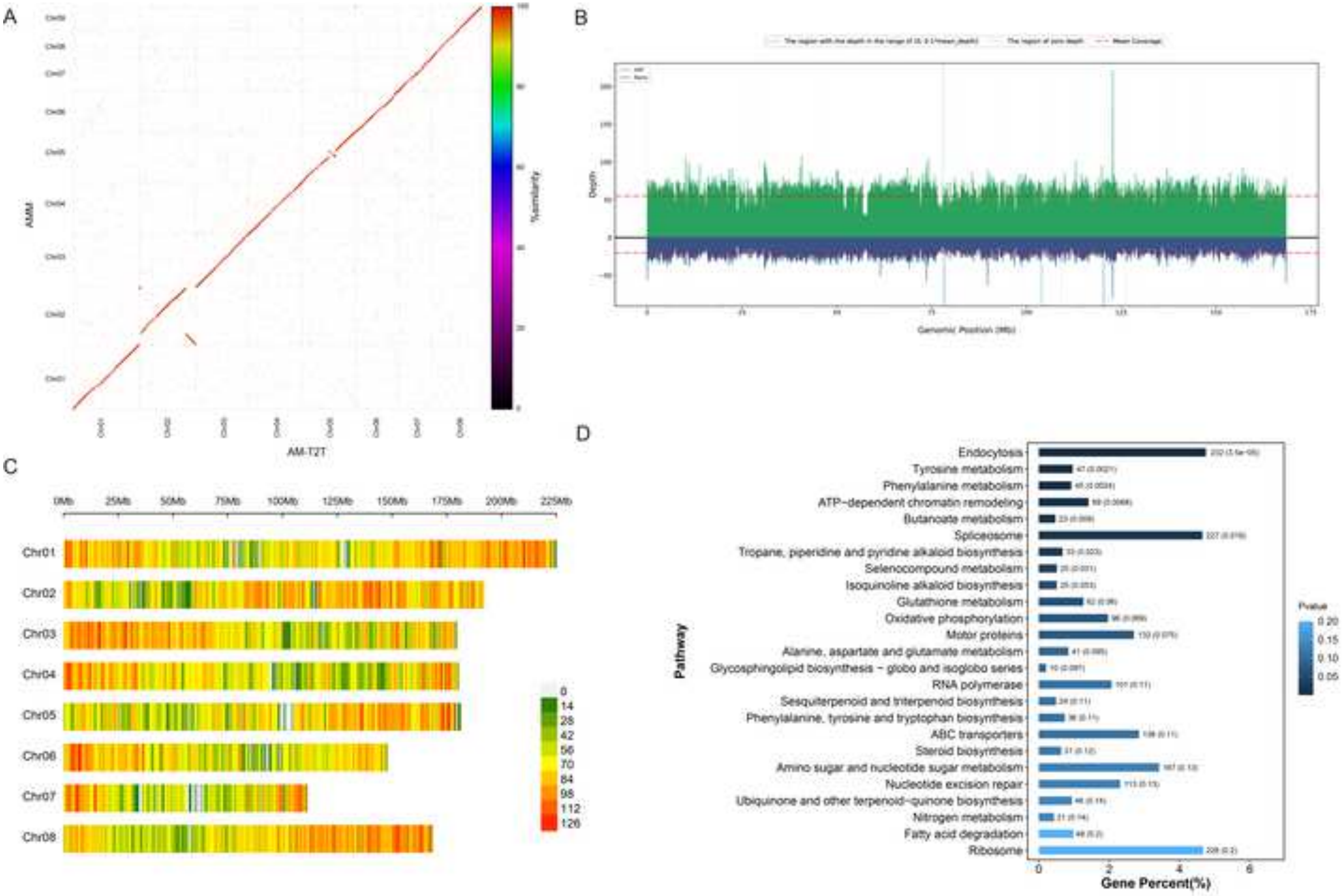

Click here to access/download
Supplementary Material
Revised Supplementary Figure.docx

Click here to access/download
Supplementary Material
Revised Supplementary Table.xlsx

Dear Dr Qin,

Your manuscript "The complete genome assembly of Astragalus membranaceus: enabling more accurate genetic research" (GIGA-D-25-00123) has been assessed by our reviewers. Although it is of interest, we are unable to consider it for publication in its current form. The reviewers have raised a number of points which we believe would improve the manuscript and may allow a revised version to be published in GigaScience.

Their reports, together with any other comments, are below. Please also take a moment to check our website at https://www.editorialmanager.com/giga/ for any additional comments that were saved as attachments.

If you are able to fully address these points, we would encourage you to submit a revised manuscript to GigaScience. Once you have made the necessary corrections, please submit online at:

https://www.editorialmanager.com/giga/

If you have forgotten your username or password please use the "Send Login Details" link to get your login information. For security reasons, your password will be reset.

Please tone down the association between medicinal properties. Please include a point-by-point within the 'Response to Reviewers' box in the submission system. Please ensure you describe additional experiments that were carried out and include a detailed rebuttal of any criticisms or requested revisions that you disagreed with (you do not need to address the biological questions/study points raised by the reviewers.). Please also ensure that your revised manuscript conforms to the journal style, which can be found in the Instructions for Authors on the journal homepage. If the data and code has been modified in the revision process please be sure to update the public versions of this too.

The due date for submitting the revised version of your article is 19 Aug 2025.

I look forward to receiving your revised manuscript soon.

Best wishes,

Hongfang Zhang
GigaScience
www.gigasciencejournal.com

Dear editor,
We sincerely thank you and the reviewers for your valuable comments and instructive advice, which help us to improve and revise our manuscript (Manuscript Number: GIGA-D-25-00123, Title: The complete genome assembly of *Astragalus*

membranaceus: enabling more accurate genetic research). We have made detailed changes accordingly. Our point-by-point responses to the reviewers' comments are provided as follows for your consideration. For ease of reviewing, all the significant changes in the revised manuscript have been highlighted in red. The list of our point-to-point responses is enclosed below with the reviewers' comments reproduced. We hope the revised manuscript can be accepted by the journal of *GigaScience*.
Thank you very much for your help!

With best regards,
Yours sincerely,

Reviewer reports:

Reviewer #1: General comments:
Astragalus membranaceus is a valuable Chinese medicinal plant. Here, Qin et al. reported a T2T genome assembly for A. membranaceus, which serves as an important genomic resource for future research. However, this manuscript is quite descriptive and lacks innovative findings. I have concerns about this manuscript, especially regarding the T2T assembly process, subsequent bioinformatic analysis, and the writing.

Major comments:
1. The authors briefly described the T2T assembly process. As the T2T assembly is the main highlight of this manuscript, I suggest the authors provide a detailed explanation of the process, especially on how the gaps are closed. For instance, they could elaborate on how the Ultra-Long data helped close these gaps, and how they filled in the remaining gaps after Ultra-Long promotion.
Re: Thanks for your valuable suggestions. In our revised manuscript, we added an analysis flowchart for T2T assembly to provide readers with a clearer understanding of the construction process of the T2T genome (see Supplementary Fig. 1). The resulting chromosomal-level assembly exhibits only 8 gaps, facilitated by the genome's high contiguity (Contig N50: 120.48 Mb). This low gap density offered significant advantages for subsequent T2T genome construction. During the gap-filling process, we utilized longer pre-assembled contigs and higher-accuracy HiFi data, consistent with the methods employed in the latest T2T genome assemblies of *Prunus campanulata* (D. Jiang et al., 2025) and *Siniperca roulei* (M. Jiang et al., 2025) published in *GigaScience*.

Reference:
Jiang, D., Li, Y., Zhuge, F., Zhou, Q., Zong, W., Liu, X., & Shen, X. (2025). The telomere-to-telomere genome of flowering cherry (*Prunus campanulata*) reveals genomic evolution of the subgenus Cerasus. ***GigaScience***, 14. https://doi.org/10.1093/gigascience/giaf009
Jiang, M., Zhao, C., Ma, F., Yin, D., Wang, C., Jian, J., & Liu, K. (2025). The

telomere-to-telomere gap-free reference genome and taxonomic reassessment of *Siniperca roulei*. ***GigaScience***, 14. https://doi.org/10.1093/gigascience/giaf068

2. The section of "Whole genome comparative analysis" present shallow description of structure variation between the T2T and the two other published genomes. The authors did not explain why such variations exist. Does it stem from the genetic difference of different accessions of A. membranaceus? Please give more evidence and explanation here, providing biological insights for reader. The comparison with the genome of another species in the genus does not really confirm the structure variation. Is there any species identification error in this study? Is the sequenced individual AMM? In addition, lines 341-348 could be moved to the previous paragraph. To me, it indicates the improvement of the T2T assembly compared to the published ones. Thus, it could be moved to the paragraph showing the quality of the assembly.

Re: Thank you for this comment. As a Data Note, this manuscript focuses on the technical aspects of T2T genome construction rather than biological insights. Its primary objective is to establish a foundational framework for future biological research, analogous to the recently published T2T genome of *Prunus campanulata* in *GigaScience* (D. Jiang et al., 2025). In this study, we provide the T2T genome resource, alongside performing gene annotation, identifying the CYP450 and UGT gene families (newly added), and constructing structural variants—all of which lay the groundwork for subsequent investigations. As for biological insights, we conducted a whole-genome comparative analysis between AM-T2T and AMM, identifying SV-genes in our revised manuscript. Pathway enrichment analysis of these SV-genes indicates their potential involvement in the survival, development, and environmental adaptation of *A. membranaceus*. Please find more details in lines 425–450 of the revised manuscript.

As you suggest, the lines 341-348 have been moved to the previous paragraph.

Reference:

Jiang, D., Li, Y., Zhuge, F., Zhou, Q., Zong, W., Liu, X., & Shen, X. (2025). The telomere-to-telomere genome of flowering cherry (*Prunus campanulata*) reveals genomic evolution of the subgenus Cerasus. ***GigaScience***, 14. https://doi.org/10.1093/gigascience/giaf009

3. What is the biological explanation of the enriched GO terms of the unique genes in A. membranaceus? How does that make sense?

Re: Thanks for your comment. While our study presents broad functional enrichment results for these unique genes, their precise biological roles remain undetermined. We propose that future research should focus on identifying key regulators of saponins and flavonoid biosynthesis pathways, along with associated transcriptional networks.

4. I highly suggest the authors to at least demonstrate an example showing 'how the T2T genome enables addressing any scientific questions'. For example, are the 46 transcription factors, located in previously unassembled regions, involved in regulating the biosynthesis of

any secondary metabolites that you mentioned in the first paragraph? Such a demonstration is a strong example of the value of a T2T genome.

Re: Thanks for your comment. Although this manuscript is structured as a Data Note, a genre that emphasizes analytical methodologies for constructing the T2T genome rather than addressing specific scientific questions, the value of our T2T genome and its relevance to scientific inquiry are highlighted in three key aspects:

1. High-quality genome assembly

   The T2T genome demonstrates exceptional quality, supported by rigorous metrics: 99.63% BUSCO completeness, a LAI of 22.67, a quality value of 57.51, a GCI (Genome Continuity Inspector) score of 36.23, and a relatively high RNA-seq mapping rate. This superior assembly serves as a more comprehensive and accurate reference genome, empowering researchers to conduct in-depth investigations into the molecular mechanisms governing critical agronomic traits.

2. Novel insights into telomeres and centromeres

   Our analysis of telomeres and centromeres establishes a foundation for subsequent cross-species evolutionary studies of centromeres, which could not have been undertaken without the T2T genome.

3. Functional implications of gene annotations

   In the revised manuscript, we present an analysis of the CYP450 gene family. Among the 46 transcription factors located in PUR regions, four were identified as CYP450 genes: *CYP71B38*, *KAO2*, *CYP71A13*, and *CYP86A1*, with *CYP71B38* detected in centromeric regions. Previous studies have suggested that CYP71 subfamily genes in *Panax ginseng* are involved in the biosynthesis of secondary metabolites, aldehydes, and flavonoids (Seitz et al., 2006). Please find more details in lines 381–400 of the revised manuscript.

Reference：

Seitz, C., Eder, C., Deiml, B., Kellner, S., Martens, S., & Forkmann, G. (2006). Cloning, Functional Identification and Sequence Analysis of Flavonoid 3′ -hydroxylase and Flavonoid 3′ ,5′ -hydroxylase cDNAs Reveals Independent Evolution of Flavonoid 3′ ,5′ -hydroxylase in the Asteraceae Family. Plant Molecular Biology, 61(3), 365-381. https://doi.org/10.1007/s11103-006-0012-0

5. The authors did not pay enough attention to the language and the logical flow between paragraphs. Especially, the logic in the introduction is difficult to follow. Please do consider re-organizing paragraphs in the introduction and results section. I have caught quite some improper usage of language and grammar mistakes. I highly require the authors to carefully edit the languages.

Re: Thanks for your reminding. Our revised manuscript has undergone professional editing by a native English speaker to ensure adherence to academic writing conventions, along with enhanced spelling accuracy and grammatical precision. Furthermore, we have refined the structure and content of paragraphs in both the Introduction and Results sections. For instance, we have comprehensively reviewed recent molecular-level research findings on *A. membranaceus* and integrated their key

conclusions into the Introduction to strengthen contextual relevance.

Reviewer #2: The manuscript presents a presents the first high-quality telomere-to-telomere genome assembly of A. membranaceus, and identified 16 telomeres and 8 centromeres distributed across 8 chromosomes. Additionally, 158.58 Mb of PURs, 2,267 unique genes, and 20,652 conserved genes were identified. Genome-wide comparison identified significant structural variations compared with prior assemblies. I read this manuscript with HUGE interests， but still have several concerns need to be clarified.

Re: Thank you for your comments and assistance. We believe that the latest updated version has addressed all the issues you mentioned.

Major:

1、I think the manuscript lacks an analysis linking the medicinal properties of Astragalus membranaceus to its T2T genome. For key genes involved in the synthesis pathways of important compounds like astragaloside and polysaccharides (such as CYP450 and UGT), it remains unclear whether they are located in previously unassembled centromeric regions. We recommend conducting and including an analysis of key genes in the synthesis pathways of important compounds.

Re: Thanks for your valuable suggestions. The CYP450 and UGT gene families have been identified in our revised manuscript. Four CYP450 genes (*CYP71B38*, *KAO2*, *CYP71A13*, and *CYP86A1*) were localized to the PUR region, with *CYP71B38* detected in centromeric regions. No UGT genes localized to either the PUR or centromeric regions. However, phylogenetic analysis revealed significant contraction of the UGT76 gene family and expansion of the UGT73 gene family in the AM genome relative to *A. thaliana*. Please find more details in lines 380–400 of the revised manuscript.

2、The authors mention that "898 genes were annotated within these PURs, including 46 transcription factors." It is unclear whether these TFs represent newly discovered families or whether they exhibit specific expression in medicinal parts such as roots and leaves. We suggest supplementing the analysis with RNA-seq tissue - specific expression heatmaps.

Re: Thanks for your suggestions. First, the expression profiles of all 898 genes within the PUR region have been added to Supplementary Table S13. Second, we have conducted heatmap visualization, tissue-specific expression analysis, and differential expression analysis for the CYP450 and UGT gene families. For further details, please refer to lines 380–400 of the revised manuscript.

3、In Figure 5, part A of the comparative genomics section does not clearly show the differences between AM-T2T and AMM-CLR. We recommend a more refined collinearity analysis, such as illustrating gene presence / absence, inversions, and other variations for each chromosome across the three genomes (AM-T2T, AM-CLR, and AM-ONT).

Re: Thanks for your comment. We have refined the manuscript’s structure and logical flow, with a particular focus on contrasting structural variations (SVs) between the AM-T2T and AMM. The core objective of comparing multiple *A. membranaceus*

genome assemblies is to validate the accuracy and integrity of the T2T genome. To corroborate the precision and comprehensiveness of our T2T assembly, we performed a series of rigorous validation analyses, including BUSCO assessment, quality value (QV) calculation, mapping rate analysis, LAI (LTR Assembly Index) evaluation, GCI (Genome Continuity Inspector), and CRAQ (Clipping information for Revealing Assembly Quality) analysis. Our comparative framework prioritizes contrasts between the AM-T2T and AMM genomes, thereby highlighting the advantages of the T2T genome in SV identification. Moving forward, we aim to identify specific SVs associated with the biosynthesis of astragalosides and flavonoids, which will be visualized for readers via dedicated structural variation diagrams. Please find more details in lines 425–450 of the revised manuscript.

Minor

1、The body paragraphs of the manuscript currently employ a hanging indent at the beginning of some paragraph. Please carefully revise the text to ensure paragraphs are formatted without indentation at the start.

Re: Revised.

2、Regarding lines 166-168: The tools and database names mentioned in this section appear to have formatting inconsistencies. Software tools such as "Next Denovo" ,"Repeat Masker", "Repeat Protein Mask," "Repeat Modeler", and "Repeats Finder" should follow standard naming conventions (e.g., RepeatMasker, RepeatProteinMask, RepeatModeler, and RepeatsFinder, written as single terms without spaces). Additionally, the database "rebase" should be capitalized as RepBase. For clarity and reproducibility, we recommend adding official website links for these tools (e.g., RepeatMasker: http://www.repeatmasker.org).

Re: Revised.

3、In line 176, the phrase "yielded 221,161 transcripts with a N50 size of 1,636" omits the unit for the N50 value. Please add the appropriate unit (e.g., bp) to ensure clarity and adherence to scientific reporting standards (revise to "N50 size of 1,636 bp").

Re: Revised.

4、In lines 196-197, the description of FPKM (Fragments Per Kilobase Million) calculation lacks sufficient detail. Please elaborate on the computational workflow, including the specific software or tools used (e.g., Cufflinks, StringTie, or custom scripts), parameters applied, and normalization steps. This will enhance reproducibility and methodological transparency.

Re: The analysis software and parameters for gene expression levels have been detailed. Please find more details in lines 231–238 of the revised manuscript.

5、In line 201, Arabidopsis thaliana is written in full. Per taxonomic conventions, after the first mention, the genus name should be abbreviated (e.g., A. thaliana). Please revise subsequent instances accordingly.

Re: Revised.

6、In line 208, the sentence "we used quarTeT (RRID:SCR_025258) version 1.1.5 TeloExplorer to identify telomeres" conflates two software tools without clarifying their relationship. To avoid confusion, please rephrase to specify whether quarTeT and TeloExplorer are integrated modules or separate tools used in tandem. For example: "we used quarTeT (RRID:SCR_025258, version 1.1.5) with TeloExplorer to identify telomeres."
Re: Revised.

7、The "Functional Annotation" section lacks critical methodological details. Please specify the databases or tools used for annotation (e.g., InterProScan, EggNOG, KEGG, or GO), the parameters applied, and the criteria for assigning functional categories. This omission hinders reproducibility and clarity.
RE: Thanks for your comment. The detailed method of functional annotation was added. Please find more details in lines 225–230 of the revised manuscript.

8、In lines 218-220, the description of the concatenated phylogenetic tree construction is incomplete. Please clarify the concatenation strategy (e.g., supermatrix approach).
RE: Thanks for your comment. The description of “The alignments were concatenated and converted into a super-gene alignment in Phylip format” have been added. Please find more details in lines 275–276 of the revised manuscript.

9、In lines 230-231, the sentence "Using different data, contigs were constructed with four assembly tools, including Hifiasm, Wtdgb2, Flye, and NextDenovo" belongs to the Methods section. Please avoid reiterating methodological steps in the Results and focus instead on presenting key findings.
Re: Revised. The sentence “Using different data, contigs were constructed with four assembly tools, including Hifiasm, Wtdgb2, Flye, and NextDenovo.” have been move to the methods section. At the same time, the language in this paragraph was polished and modified.

10、In line 310, In line 310, the presentation of results lacks specific data. It is recommended to include a table with the expression data of the genes.
Re: Revised. The gene expression matrix has been provided in Supplementary Table S15.

11、In line 316, the Latin names in "A.mongholicus, C.arietinum, and M.truncatula" lack spacing between the genus abbreviation and the species epithet (e.g., "A. mongholicus" instead of "A.mongholicus"). Ensure all Latin names follow the standard format (Genus abbreviation + space + species*, italicized).
Re: Revised.

12、For the phylogenetic tree in Figure S6: Add branch support values (e.g., bootstrap percentages, posterior probabilities) to key nodes. Highlight focal species or clades of interest using distinct colors or symbols to improve visual clarity.

Re: Revised. Bootstrap values have been added. Please find more details in Figure S8.

13、In lines 317-319, the phrase "out of the 19,271..." lacks context regarding the comparison species. Revise to explicitly state the reference group (e.g., "Compared to [species name], out of the 19,271...").
Re: This study does not include comparisons to the most recent common ancestor for contraction/expansion inference, as the scope is limited to gene family enumeration and phylogenetic placement. If it is an analysis of gene family contraction and expansion, according to your suggestion, "Compared with the most recent common ancestor" should be added.

14、Ensure genus abbreviations in Figure 4 (e.g., "A.mongholicus") include a space after the period (e.g., "A. mongholicus"). The label "AM-T2T" in Figure 4B should not be italicized, as it refers to an assembly name rather than a taxonomic term.
Re: Revised. Due to the addition of analysis of the CYP450 and UGT gene families, the original Figure 4 has been changed to Figure 5 in our revised article.

15、The manuscript lacks a description of the comparative genomic analysis methods and it is recommended to supplement this information.
Re: The method of "Identification of structural variants between AM-T2T and AMM", and "Identification of PUR regions", has been added in our revised manuscript. Please find more details in lines 281–289 of the revised manuscript.
