# Supplementary material for: The complete genome assembly of Astragalus membranaceus: enabling more accurate genetic research
Source: Gigascience. 2025 Oct 1;14:giaf117. doi: 10.1093/gigascience/giaf117 (PMC12486382; doi:10.1093/gigascience/giaf117)
Supplement: giaf117_Authors_Response_To_Reviewer_Comments_Original_Submission [file giaf117_authors_response_to_reviewer_comments_original_submission.pdf]

Author's Response To Reviewer Comments

Dear editor,
We sincerely thank you and the reviewers for your valuable comments and instructive advice, which help us to improve and revise our manuscript (Manuscript Number: GIGA-D-25-00123, Title: The complete genome assembly of Astragalus membranaceus: enabling more accurate genetic research). We have made detailed changes accordingly. Our point-by-point responses to the reviewers' comments are provided as follows for your consideration. For ease of reviewing, all the significant changes in the revised manuscript have been highlighted in red. The list of our point-to-point responses is enclosed below with the reviewers' comments reproduced. We hope the revised manuscript can be accepted by the journal of GigaScience.
Thank you very much for your help!

With best regards,
Yours sincerely,

Reviewer reports:

Reviewer #1: General comments:
Astragalus membranaceus is a valuable Chinese medicinal plant. Here, Qin et al. reported a T2T genome assembly for A. membranaceus, which serves as an important genomic resource for future research. However, this manuscript is quite descriptive and lacks innovative findings. I have concerns about this manuscript, especially regarding the T2T assembly process, subsequent bioinformatic analysis, and the writing.

Major comments:
1. The authors briefly described the T2T assembly process. As the T2T assembly is the main highlight of this manuscript, I suggest the authors provide a detailed explanation of the process, especially on how the gaps are closed. For instance, they could elaborate on how the Ultra-Long data helped close these gaps, and how they filled in the remaining gaps after Ultra-Long promotion.
Re: Thanks for your valuable suggestions. In our revised manuscript, we added an analysis flowchart for T2T assembly to provide readers with a clearer understanding of the construction process of the T2T genome (see Supplementary Fig. 1). The resulting chromosomal-level assembly exhibits only 8 gaps, facilitated by the genome's high contiguity (Contig N50: 120.48 Mb). This low gap density offered significant advantages for subsequent T2T genome construction. During the gap-filling process, we utilized longer pre-assembled contigs and higher-accuracy HiFi data, consistent with the methods employed in the latest T2T genome assemblies of Prunus campanulata (D. Jiang et al., 2025) and Siniperca roulei (M. Jiang et al., 2025) published in GigaScience.

Reference:
Jiang, D., Li, Y., Zhuge, F., Zhou, Q., Zong, W., Liu, X., & Shen, X. (2025). The telomere-to-telomere genome of flowering cherry (Prunus campanulata) reveals genomic evolution of the subgenus Cerasus. GigaScience, 14. https://doi.org/10.1093/gigascience/giaf009
Jiang, M., Zhao, C., Ma, F., Yin, D., Wang, C., Jian, J., & Liu, K. (2025). The telomere-to-telomere gap-free reference genome and taxonomic reassessment of Siniperca roulei. GigaScience, 14. https://doi.org/10.1093/gigascience/giaf068

2. The section of "Whole genome comparative analysis" present shallow description of structure variation between the T2T and the two other published genomes. The authors did not explain why such variations exist. Does it stem from the genetic difference of different accessions of A. membranaceus? Please give more evidence and explanation here, providing biological insights for reader. The comparison with the genome of another species in the genus does not really confirm the structure variation. Is there any species identification error in this study? Is the sequenced individual AMM? In addition, lines 341-348 could be moved to the previous paragraph. To me, it indicates the improvement of the T2T assembly compared to the published ones. Thus, it could be moved to the paragraph showing the quality of the assembly.

Re: Thank you for this comment. As a Data Note, this manuscript focuses on the technical aspects of T2T genome construction rather than biological insights. Its primary objective is to establish a foundational framework for future biological research, analogous to the recently published T2T genome of Prunus campanulata in GigaScience (D. Jiang et al., 2025). In this study, we provide the T2T genome resource, alongside performing gene annotation, identifying the CYP450 and UGT gene families (newly added), and constructing structural variants—all of which lay the groundwork for subsequent investigations. As for biological insights, we conducted a whole-genome comparative analysis between AM-T2T and AMM, identifying SV-genes in our revised manuscript. Pathway enrichment analysis of these SV-genes indicates their potential involvement in the survival, development, and environmental adaptation of A. membranaceus. Please find more details in lines 425–450 of the revised manuscript.
As you suggest, the lines 341-348 have been moved to the previous paragraph.

Reference:
Jiang, D., Li, Y., Zhuge, F., Zhou, Q., Zong, W., Liu, X., & Shen, X. (2025). The telomere-to-telomere genome of flowering cherry (Prunus campanulata) reveals genomic evolution of the subgenus Cerasus. GigaScience, 14. https://doi.org/10.1093/gigascience/giaf009

3. What is the biological explanation of the enriched GO terms of the unique genes in A. membranaceus? How does that make sense?
Re: Thanks for your comment. While our study presents broad functional enrichment results for these unique genes, their precise biological roles remain undetermined. We propose that future research should focus on identifying key regulators of saponins and flavonoid biosynthesis pathways, along with associated transcriptional networks.

4. I highly suggest the authors to at least demonstrate an example showing 'how the T2T genome enables addressing any scientific questions'. For example, are the 46 transcription factors, located in previously unassembled regions, involved in regulating the biosynthesis of any secondary metabolites that you mentioned in the first paragraph? Such a demonstration is a strong example of the value of a T2T genome.
Re: Thanks for your comment. Although this manuscript is structured as a Data Note, a genre that emphasizes analytical methodologies for constructing the T2T genome rather than addressing specific scientific questions, the value of our T2T genome and its relevance to scientific inquiry are highlighted in three key aspects:
1.High-quality genome assembly
The T2T genome demonstrates exceptional quality, supported by rigorous metrics: 99.63% BUSCO completeness, a LAI of 22.67, a quality value of 57.51, a GCI (Genome Continuity Inspector) score of 36.23, and a relatively high RNA-seq mapping rate. This superior assembly serves as a more comprehensive and accurate reference genome, empowering researchers to conduct in-depth investigations into the molecular mechanisms governing critical agronomic traits.
2.Novel insights into telomeres and centromeres
Our analysis of telomeres and centromeres establishes a foundation for subsequent cross-species evolutionary studies of centromeres, which could not have been undertaken without the T2T genome.
3.Functional implications of gene annotations
In the revised manuscript, we present an analysis of the CYP450 gene family. Among the 46 transcription factors located in PUR regions, four were identified as CYP450 genes: CYP71B38, KAO2, CYP71A13, and CYP86A1, with CYP71B38 detected in centromeric regions. Previous studies have suggested that CYP71 subfamily genes in Panax ginseng are involved in the biosynthesis of secondary metabolites, aldehydes, and flavonoids (Seitz et al., 2006). Please find more details in lines 381–400 of the revised manuscript.

Reference：
Seitz, C., Eder, C., Deiml, B., Kellner, S., Martens, S., & Forkmann, G. (2006). Cloning, Functional Identification and Sequence Analysis of Flavonoid 3′-hydroxylase and Flavonoid 3′,5′-hydroxylase cDNAs Reveals Independent Evolution of Flavonoid 3′,5′-hydroxylase in the Asteraceae Family. Plant Molecular Biology, 61(3), 365-381. https://doi.org/10.1007/s11103-006-0012-0

5. The authors did not pay enough attention to the language and the logical flow between paragraphs. Especially, the logic in the introduction is difficult to follow. Please do consider re-organizing paragraphs in the introduction and results section. I have caught quite some improper usage of language and grammar

mistakes. I highly require the authors to carefully edit the languages.
Re: Thanks for your reminding. Our revised manuscript has undergone professional editing by a native English speaker to ensure adherence to academic writing conventions, along with enhanced spelling accuracy and grammatical precision. Furthermore, we have refined the structure and content of paragraphs in both the Introduction and Results sections. For instance, we have comprehensively reviewed recent molecular-level research findings on A. membranaceus and integrated their key conclusions into the Introduction to strengthen contextual relevance.

Reviewer #2: The manuscript presents a presents the first high-quality telomere-to-telomere genome assembly of A. membranaceus, and identified 16 telomeres and 8 centromeres distributed across 8 chromosomes. Additionally, 158.58 Mb of PURs, 2,267 unique genes, and 20,652 conserved genes were identified. Genome-wide comparison identified significant structural variations compared with prior assemblies. I read this manuscript with HUGE interests, but still have several concerns need to be clarified.
Re: Thank you for your comments and assistance. We believe that the latest updated version has addressed all the issues you mentioned.

Major:
1、I think the manuscript lacks an analysis linking the medicinal properties of Astragalus membranaceus to its T2T genome. For key genes involved in the synthesis pathways of important compounds like astragaloside and polysaccharides (such as CYP450 and UGT), it remains unclear whether they are located in previously unassembled centromeric regions. We recommend conducting and including an analysis of key genes in the synthesis pathways of important compounds.
Re: Thanks for your valuable suggestions. The CYP450 and UGT gene families have been identified in our revised manuscript. Four CYP450 genes (CYP71B38, KAO2, CYP71A13, and CYP86A1) were localized to the PUR region, with CYP71B38 detected in centromeric regions. No UGT genes localized to either the PUR or centromeric regions. However, phylogenetic analysis revealed significant contraction of the UGT76 gene family and expansion of the UGT73 gene family in the AM genome relative to A. thaliana. Please find more details in lines 380–400 of the revised manuscript.

2、The authors mention that "898 genes were annotated within these PURs, including 46 transcription factors." It is unclear whether these TFs represent newly discovered families or whether they exhibit specific expression in medicinal parts such as roots and leaves. We suggest supplementing the analysis with RNA-seq tissue - specific expression heatmaps.
Re: Thanks for your suggestions. First, the expression profiles of all 898 genes within the PUR region have been added to Supplementary Table S13. Second, we have conducted heatmap visualization, tissue-specific expression analysis, and differential expression analysis for the CYP450 and UGT gene families. For further details, please refer to lines 380–400 of the revised manuscript.

3、In Figure 5, part A of the comparative genomics section does not clearly show the differences between AM-T2T and AMM-CLR. We recommend a more refined collinearity analysis, such as illustrating gene presence / absence, inversions, and other variations for each chromosome across the three genomes (AM-T2T, AM-CLR, and AM-ONT).
Re: Thanks for your comment. We have refined the manuscript's structure and logical flow, with a particular focus on contrasting structural variations (SVs) between the AM-T2T and AMM. The core objective of comparing multiple A. membranaceus genome assemblies is to validate the accuracy and integrity of the T2T genome. To corroborate the precision and comprehensiveness of our T2T assembly, we performed a series of rigorous validation analyses, including BUSCO assessment, quality value (QV) calculation, mapping rate analysis, LAI (LTR Assembly Index) evaluation, GCI (Genome Continuity Inspector), and CRAQ (Clipping information for Revealing Assembly Quality) analysis. Our comparative framework prioritizes contrasts between the AM-T2T and AMM genomes, thereby highlighting the advantages of the T2T genome in SV identification. Moving forward, we aim to identify specific SVs associated with the biosynthesis of astragalosides and flavonoids, which will be visualized for readers via dedicated structural variation diagrams. Please find more details in lines 425–450 of the revised manuscript.

Minor

1、The body paragraphs of the manuscript currently employ a hanging indent at the beginning of some paragraph. Please carefully revise the text to ensure paragraphs are formatted without indentation at the start.
Re: Revised.

2、Regarding lines 166-168: The tools and database names mentioned in this section appear to have formatting inconsistencies. Software tools such as "Next Denovo" ,"Repeat Masker", "Repeat Protein Mask," "Repeat Modeler", and "Repeats Finder" should follow standard naming conventions (e.g., RepeatMasker, RepeatProteinMask, RepeatModeler, and RepeatsFinder, written as single terms without spaces). Additionally, the database "rebase" should be capitalized as RepBase. For clarity and reproducibility, we recommend adding official website links for these tools (e.g., RepeatMasker: http://www.repeatmasker.org).
Re: Revised.

3、In line 176, the phrase "yielded 221,161 transcripts with a N50 size of 1,636" omits the unit for the N50 value. Please add the appropriate unit (e.g., bp) to ensure clarity and adherence to scientific reporting standards (revise to "N50 size of 1,636 bp").
Re: Revised.

4、In lines 196-197, the description of FPKM (Fragments Per Kilobase Million) calculation lacks sufficient detail. Please elaborate on the computational workflow, including the specific software or tools used (e.g., Cufflinks, StringTie, or custom scripts), parameters applied, and normalization steps. This will enhance reproducibility and methodological transparency.
Re: The analysis software and parameters for gene expression levels have been detailed. Please find more details in lines 231–238 of the revised manuscript.

5、In line 201, Arabidopsis thaliana is written in full. Per taxonomic conventions, after the first mention, the genus name should be abbreviated (e.g., A. thaliana). Please revise subsequent instances accordingly.
Re: Revised.

6、In line 208, the sentence "we used quarTeT (RRID:SCR_025258) version 1.1.5 TeloExplorer to identify telomeres" conflates two software tools without clarifying their relationship. To avoid confusion, please rephrase to specify whether quarTeT and TeloExplorer are integrated modules or separate tools used in tandem. For example: "we used quarTeT (RRID:SCR_025258, version 1.1.5) with TeloExplorer to identify telomeres."
Re: Revised.

7、The "Functional Annotation" section lacks critical methodological details. Please specify the databases or tools used for annotation (e.g., InterProScan, EggNOG, KEGG, or GO), the parameters applied, and the criteria for assigning functional categories. This omission hinders reproducibility and clarity.
RE: Thanks for your comment. The detailed method of functional annotation was added. Please find more details in lines 225–230 of the revised manuscript.

8、In lines 218-220, the description of the concatenated phylogenetic tree construction is incomplete. Please clarify the concatenation strategy (e.g., supermatrix approach).
RE: Thanks for your comment. The description of "The alignments were concatenated and converted into a super-gene alignment in Phylip format" have been added. Please find more details in lines 275–276 of the revised manuscript.

9、In lines 230-231, the sentence "Using different data, contigs were constructed with four assembly tools, including Hifiasm, Wtdgb2, Flye, and NextDenovo" belongs to the Methods section. Please avoid reiterating methodological steps in the Results and focus instead on presenting key findings.
Re: Revised. The sentence "Using different data, contigs were constructed with four assembly tools, including Hifiasm, Wtdgb2, Flye, and NextDenovo." have been move to the methods section. At the same time, the language in this paragraph was polished and modified.

10、In line 310, In line 310, the presentation of results lacks specific data. It is recommended to include a table with the expression data of the genes.
Re: Revised. The gene expression matrix has been provided in Supplementary Table S15.

11、In line 316, the Latin names in "A.mongholicus, C.arietinum, and M.truncatula" lack spacing between the genus abbreviation and the species epithet (e.g., "A. mongholicus" instead of "A.mongholicus"). Ensure all Latin names follow the standard format (Genus abbreviation + space + species*, italicized).
Re: Revised.

12、For the phylogenetic tree in Figure S6: Add branch support values (e.g., bootstrap percentages, posterior probabilities) to key nodes. Highlight focal species or clades of interest using distinct colors or symbols to improve visual clarity.
Re: Revised. Bootstrap values have been added. Please find more details in Figure S8.

13、In lines 317-319, the phrase "out of the 19,271..." lacks context regarding the comparison species. Revise to explicitly state the reference group (e.g., "Compared to [species name], out of the 19,271...").
Re: This study does not include comparisons to the most recent common ancestor for contraction/expansion inference, as the scope is limited to gene family enumeration and phylogenetic placement. If it is an analysis of gene family contraction and expansion, according to your suggestion, "Compared with the most recent common ancestor" should be added.

14、Ensure genus abbreviations in Figure 4 (e.g., "A.mongholicus") include a space after the period (e.g., "A. mongholicus"). The label "AM-T2T" in Figure 4B should not be italicized, as it refers to an assembly name rather than a taxonomic term.
Re: Revised. Due to the addition of analysis of the CYP450 and UGT gene families, the original Figure 4 has been changed to Figure 5 in our revised article.

15、The manuscript lacks a description of the comparative genomic analysis methods and it is recommended to supplement this information.
Re: The method of "Identification of structural variants between AM-T2T and AMM", and "Identification of PUR regions", has been added in our revised manuscript. Please find more details in lines 281–289 of the revised manuscript.

Editorial Manager®

Cookie Preference Center

We use cookies which are necessary to make our site work. We may also use additional cookies to analyze, improve and personalize our content and your digital experience. For more information, see our Cookie Policy.

You may choose not to allow some types of cookies. However, blocking some types may

impact your experience of our site and the services we are able to offer. See the different category headings below to find out more or change your settings.

Allow all

Manage Consent Preferences

Strictly Necessary Cookies

Always active

These cookies are necessary for the website to function and cannot be switched off in our systems. They are usually only set in response to actions made by you which amount to a request for services, such as setting your privacy preferences, logging in or filling in forms. You can set your browser to block or alert you about these cookies, but some parts of the site will not then work.

Functional Cookies

These cookies enable the website to provide enhanced functionality and personalisation. They may be set by us or by third party providers whose services we have added to our pages. If you do not allow these cookies then some or all of these services may not function properly.

Performance Cookies

These cookies allow us to count visits and traffic sources so we can measure and improve the performance of our site. They help us to know which pages are the most and least popular and see how visitors move around the site.

Targeting Cookies

These cookies may be set through our site by our advertising partners. They may be used by those companies to build a profile of your interests and show you relevant adverts on other sites. If you do not allow these cookies, you will experience less targeted advertising.

Cookie List

Clear

Apply Cancel

Consent Leg.Interest

Confirm my choices

Powered by onetrus
