# Supplementary material for: The complete genome assembly of Astragalus membranaceus: enabling more accurate genetic research
Source: Gigascience. 2025 Oct 1;14:giaf117. doi: 10.1093/gigascience/giaf117 (PMC12486382; doi:10.1093/gigascience/giaf117)
Supplement: giaf117_Reviewer_1_Report_Original_Submission — Li Wang -- 5/7/2025 [file giaf117_reviewer_1_report_original_submission.pdf]

Reviewer Report

Title: The complete genome assembly of Astragalus membranaceus: enabling more accurate genetic research

Version: Original Submission **Date:** 5/7/2025

Reviewer name: Li Wang

Reviewer Comments to Author:

General comments:Astragalus membranaceus is a valuable Chinese medicinal plant. Here, Qin et al. reported a T2T genome assembly for A. membranaceus, which serves as an important genomic resource for future research. However, this manuscript is quite descriptive and lacks innovative findings. I have concerns about this manuscript, especially regarding the T2T assembly process, subsequent bioinformatic analysis, and the writing.Major comments:1. The authors briefly described the T2T assembly process. As the T2T assembly is the main highlight of this manuscript, I suggest the authors provide a detailed explanation of the process, especially on how the gaps are closed. For instance, they could elaborate on how the Ultra-Long data helped close these gaps, and how they filled in the remaining gaps after Ultra-Long promotion.2. The section of "Whole genome comparative analysis" present shallow description of structure variation between the T2T and the two other published genomes. The authors did not explain why such variations exist. Does it stem from the genetic difference of different accessions of A. membranaceus? Please give more evidence and explanation here, providing biological insights for reader. The comparison with the genome of another species in the genus does not really confirm the structure variation. Is there any species identification error in this study? Is the sequenced individual AMM? In addition, lines 341-348 could be moved to the previous paragraph. To me, it indicates the improvement of the T2T assembly compared to the published ones. Thus, it could be moved to the paragraph showing the quality of the assembly.3. What is the biological explanation of the enriched GO terms of the unique genes in A. membranaceus? How does that make sense?4. I highly suggest the authors to at least demonstrate an example showing 'how the T2T genome enables addressing any scientific questions'. For example, are the 46 transcription factors, located in previously unassembled regions, involved in regulating the biosynthesis of any secondary metabolites that you mentioned in the first paragraph? Such a demonstration is a strong example of the value of a T2T genome.5. The authors did not pay enough attention to the language and the logical flow between paragraphs. Especially, the logic in the introduction is difficult to follow. Please do consider re-organizing paragraphs in the introduction and results section. I have caught quite some improper usage of language and grammar mistakes. I highly require the authors to carefully edit the languages.

Level of Interest

Please indicate how interesting you found the manuscript: Choose an item.

Quality of Written English

Please indicate the quality of language in the manuscript: Choose an item.

Declaration of Competing Interests

Please complete a declaration of competing interests, considering the following questions:

- Have you in the past five years received reimbursements, fees, funding, or salary from an organisation that may in any way gain or lose financially from the publication of this manuscript, either now or in the future?
- Do you hold any stocks or shares in an organisation that may in any way gain or lose financially from the publication of this manuscript, either now or in the future?
- Do you hold or are you currently applying for any patents relating to the content of the manuscript?
- Have you received reimbursements, fees, funding, or salary from an organization that holds or has applied for patents relating to the content of the manuscript?
- Do you have any other financial competing interests?
- Do you have any non-financial competing interests in relation to this paper?

If you can answer no to all of the above, write 'I declare that I have no competing interests' below. If your reply is yes to any, please give details below.

I declare that I have no competing interests

I agree to the open peer review policy of the journal. I understand that my name will be included on my report to the authors and, if the manuscript is accepted for publication, my named report including any attachments I upload will be posted on the website along with the authors' responses. I agree for my report to be made available under an Open Access Creative Commons CC-BY license (http://creativecommons.org/licenses/by/4.0/). I understand that any comments which I do not wish to be included in my named report can be included as confidential comments to the editors, which will not be published.

Choose an item.

To further support our reviewers, we have joined with Publons, where you can gain additional credit to further highlight your hard work (see: https://publons.com/journal/530/gigascience). On publication of this paper, your review will be automatically added to Publons, you can then choose whether or not to claim your Publons credit. I understand this statement.

Yes Choose an item.
