# Supplementary material for: The complete genome assembly of Astragalus membranaceus: enabling more accurate genetic research
Source: Gigascience. 2025 Oct 1;14:giaf117. doi: 10.1093/gigascience/giaf117 (PMC12486382; doi:10.1093/gigascience/giaf117)
Supplement: giaf117_Reviewer_2_Report_Original_Submission — Wei Sun -- 5/15/2025 [file giaf117_reviewer_2_report_original_submission.pdf]

Reviewer Report

Title: The complete genome assembly of Astragalus membranaceus: enabling more accurate genetic research

Version: Original Submission **Date:** 5/15/2025

Reviewer name: Wei Sun

Reviewer Comments to Author:

The manuscript presents a presents the first high-quality telomere-to-telomere genome assembly of A. membranaceus, and identified 16 telomeres and 8 centromeres distributed across 8 chromosomes. Additionally, 158.58 Mb of PURs, 2,267 unique genes, and 20,652 conserved genes were identified. Genome-wide comparison identified significant structural variations compared with prior assemblies. I read this manuscript with HUGE interestsï¼Œbut still have several concerns need to be clarified.Major:1ã€�I think the manuscript lacks an analysis linking the medicinal properties of Astragalus membranaceus to its T2T genome. For key genes involved in the synthesis pathways of important compounds like astragaloside and polysaccharides (such as CYP450 and UGT), it remains unclear whether they are located in previously unassembled centromeric regions. We recommend conducting and including an analysis of key genes in the synthesis pathways of important compounds.2ã€�The authors mention that "898 genes were annotated within these PURs, including 46 transcription factors." It is unclear whether these TFs represent newly discovered families or whether they exhibit specific expression in medicinal parts such as roots and leaves. We suggest supplementing the analysis with RNA-seq tissue - specific expression heatmaps.3ã€�In Figure 5, part A of the comparative genomics section does not clearly show the differences between AM-T2T and AMM-CLR. We recommend a more refined collinearity analysis, such as illustrating gene presence / absence, inversions, and other variations for each chromosome across the three genomes (AM-T2T, AM-CLR, and AM-ONT).Minor1ã€�The body paragraphs of the manuscript currently employ a hanging indent at the beginning of some paragraph. Please carefully revise the text to ensure paragraphs are formatted without indentation at the start.2ã€�Regarding lines 166-168: The tools and database names mentioned in this section appear to have formatting inconsistencies. Software tools such as "Next Denovo" ,"Repeat Masker", "Repeat Protein Mask," "Repeat Modeler", and "Repeats Finder" should follow standard naming conventions (e.g., RepeatMasker, RepeatProteinMask, RepeatModeler, and RepeatsFinder, written as single terms without spaces). Additionally, the database "rebase" should be capitalized as RepBase. For clarity and reproducibility, we recommend adding official website links for these tools (e.g., RepeatMasker: http://www.repeatmasker.org).3ã€�In line 176, the phrase "yielded 221,161 transcripts with a N50 size of 1,636" omits the unit for the N50 value. Please add the appropriate unit (e.g., bp) to ensure clarity and adherence to scientific reporting standards (revise to "N50 size of 1,636 bp").4ã€�In lines 196-197, the description of FPKM (Fragments Per Kilobase Million) calculation lacks sufficient detail. Please elaborate on the computational workflow, including the specific software or tools used (e.g., Cufflinks, StringTie, or custom scripts), parameters applied, and normalization steps. This will enhance reproducibility and methodological transparency.5ã€�In line 201,

Arabidopsis thaliana is written in full. Per taxonomic conventions, after the first mention, the genus name should be abbreviated (e.g., A. thaliana). Please revise subsequent instances accordingly.6ã€�In line 208, the sentence "we used quarTeT (RRID:SCR_025258) version 1.1.5 TeloExplorer to identify telomeres" conflates two software tools without clarifying their relationship. To avoid confusion, please rephrase to specify whether quarTeT and TeloExplorer are integrated modules or separate tools used in tandem. For example: "we used quarTeT (RRID:SCR_025258, version 1.1.5) with TeloExplorer to identify telomeres."7ã€�The "Functional Annotation" section lacks critical methodological details. Please specify the databases or tools used for annotation (e.g., InterProScan, EggNOG, KEGG, or GO), the parameters applied, and the criteria for assigning functional categories. This omission hinders reproducibility and clarity.8ã€�In lines 218-220, the description of the concatenated phylogenetic tree construction is incomplete. Please clarify the concatenation strategy (e.g., supermatrix approach).9ã€�In lines 230-231, the sentence "Using different data, contigs were constructed with four assembly tools, including Hifiasm, Wtdgb2, Flye, and NextDenovo" belongs to the Methods section. Please avoid reiterating methodological steps in the Results and focus instead on presenting key findings.10ã€�In line 310, In line 310, the presentation of results lacks specific data. It is recommended to include a table with the expression data of the genes.11ã€�In line 316, the Latin names in "A.mongholicus, C.arietinum, and M.truncatula" lack spacing between the genus abbreviation and the species epithet (e.g., "A. mongholicus" instead of "A.mongholicus"). Ensure all Latin names follow the standard format (Genus abbreviation + space + species*, italicized).12ã€�For the phylogenetic tree in Figure S6: Add branch support values (e.g., bootstrap percentages, posterior probabilities) to key nodes. Highlight focal species or clades of interest using distinct colors or symbols to improve visual clarity.13ã€�In lines 317-319, the phrase "out of the 19,271..." lacks context regarding the comparison species. Revise to explicitly state the reference group (e.g., "Compared to [species name], out of the 19,271...").14ã€�Ensure genus abbreviations in Figure 4 (e.g., "A.mongholicus") include a space after the period (e.g., "A. mongholicus"). The label "AM-T2T" in Figure 4B should not be italicized, as it refers to an assembly name rather than a taxonomic term.15ã€�The manuscript lacks a description of the comparative genomic analysis methods and it is recommended to supplement this information.

Level of Interest

Please indicate how interesting you found the manuscript: Choose an item.

Quality of Written English

Please indicate the quality of language in the manuscript: Choose an item.

Declaration of Competing Interests

Please complete a declaration of competing interests, considering the following questions:

- Have you in the past five years received reimbursements, fees, funding, or salary from an organisation that may in any way gain or lose financially from the publication of this manuscript, either now or in the future?
- Do you hold any stocks or shares in an organisation that may in any way gain or lose financially from the publication of this manuscript, either now or in the future?
- Do you hold or are you currently applying for any patents relating to the content of the manuscript?
- Have you received reimbursements, fees, funding, or salary from an organization that holds or has applied for patents relating to the content of the manuscript?
- Do you have any other financial competing interests?
- Do you have any non-financial competing interests in relation to this paper?

If you can answer no to all of the above, write 'I declare that I have no competing interests' below. If your reply is yes to any, please give details below.

I declare that I have no competing interests

I agree to the open peer review policy of the journal. I understand that my name will be included on my report to the authors and, if the manuscript is accepted for publication, my named report including any attachments I upload will be posted on the website along with the authors' responses. I agree for my report to be made available under an Open Access Creative Commons CC-BY license (http://creativecommons.org/licenses/by/4.0/). I understand that any comments which I do not wish to be included in my named report can be included as confidential comments to the editors, which will not be published.

Choose an item.

To further support our reviewers, we have joined with Publons, where you can gain additional credit to further highlight your hard work (see: https://publons.com/journal/530/gigascience). On publication of this paper, your review will be automatically added to Publons, you can then choose whether or not to claim your Publons credit. I understand this statement.

Yes Choose an item.
